# Humilisin E: Strategy for the Synthesis and Access to the Functionalized Bicyclic Core

**DOI:** 10.1021/acs.joc.4c00358

**Published:** 2024-04-26

**Authors:** Prachi Verma, Rajanish R. Pallerla, Aino Rolig, Petri M. Pihko

**Affiliations:** Department of Chemistry and NanoScience Center, University of Jyväskylä, P.O. Box 35, FI-40014 Jyväskylä, Finland

## Abstract

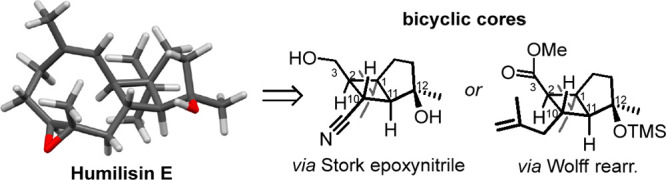

Humilisin E is a diterpenoid possessing a rare epoxidized cyclononene *trans*-fused with a bicyclo[3.2.0]heptane core. We have identified the *P* atropisomer of the corresponding cyclononadiene as a potential biosynthetic/synthetic precursor to humilisin E and reported two different strategies for the stereocontrolled synthesis of the appropriately functionalized bicyclic cores of humilisin E. The first route involves a Stork epoxynitrile cyclization via a Mg alkoxide, and the second, more stereoselective approach utilizes the Wolff rearrangement as the key step.

Humilisins E and F [**1a** and **1b**, respectively (Figure 1)] are novel terpenoids isolated from the South China Sea soft coral *Sinularia humilis*. They possess a highly substituted cyclobutane ring fused with cyclopentane and cyclononene ring systems.^1^ Their structures also include an epoxide ring fused with the cyclononene ring (at C7–C8) and a hydroxy/hydroperoxide group at C12. These functionalities may be introduced in late-stage oxidation processes from the terpene precursor during the biosynthesis of **1a** and **1b**.

**Figure 1:**
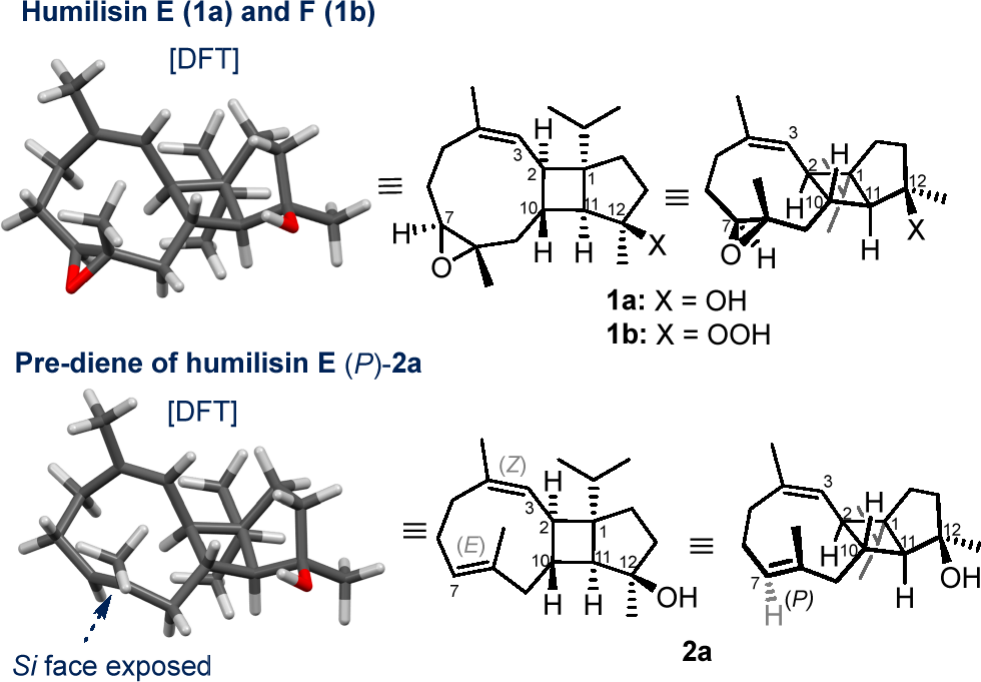
Minimized [DFT (see the SI for details)] structures of humilisin E (**1a**), humilisin F (**1b**), and the potential prediene (*P*)-**2a**.

From both synthetic and biosynthetic points of view, potential precursors might be the corresponding (3*Z*,7*E*)-dienes **2a** and **2b** (Figure 1). The *P* atropisomer of **2a** possesses a configuration that leads to **1a** via the epoxidation of the 7*E* alkenyl group. In (*P*)-**2a**, the *si* face of the (7*E*)-alkene is exposed, while the approach to the (3*Z*)-alkene is sterically hindered by the *i*-Pr group at C1 (Figure 1). This analysis suggests that late-stage regio- and stereoselective epoxidation of **2a** or **2b** to give humilisin E or F (**1a** or **1b)**, respectively, should be possible. Epoxidation of **2a** and **2b** represents a potential biosynthetic route to **1a** and **1b**, respectively. Preliminary density functional theory (DFT) calculations suggest that (*P*)-**2a** is thermodynamically more stable than (*M*)-**2a** by 14 kJ/mol (see the Supporting Information).

Synthetic access to (*P*)-**2a** requires the construction of a fused cyclononadiene. Synthesis of nine-membered rings is generally challenging, and in spite of their importance in natural products and medicinally important compounds,^2^ their synthesis often requires special strategies such as conformational control to help the cyclization step.^3,4^ We propose that the conformational rigidity of the cyclobutane ring and the *trans*-disposed substituents at C2 and C10 of the cyclobutane could assist in the closure of the cyclononadiene by restricting the conformational freedom (Scheme 1). To test this hypothesis, we required access to the bicyclic cyclobutane–cyclopentane core of **1a** and **2a** [e.g., via **3** (Scheme 1)]. Herein, we report the successful stereoselective synthesis of the functionalized bicyclo[3.2.0]heptane^5−7^ subunit of humilisin E via two different approaches.

**Scheme 1:**
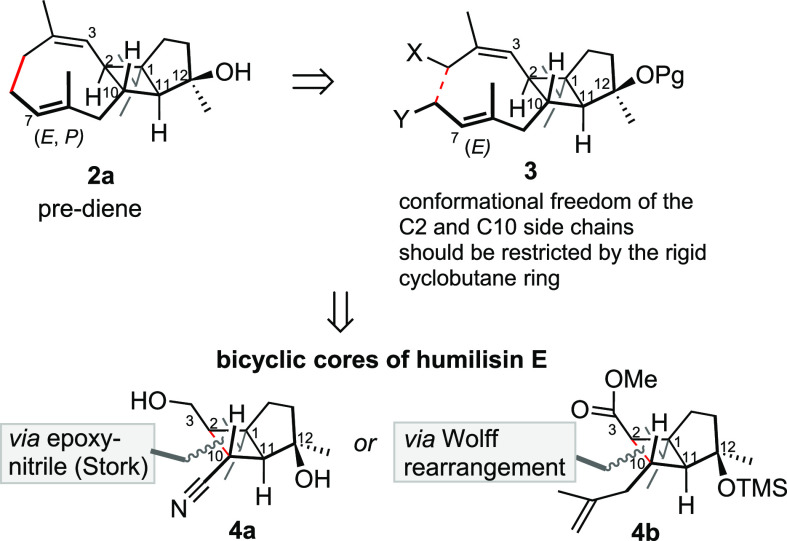
Retrosynthetic Analysis of **2** via Conformationally Restricted Intermediate **3** and Key Core Fragments **4a** and **4b** X and Y are functional groups for the cyclization reaction.

## Results and Discussion

Our first approach involved the epoxynitrile cyclization reported by Stork, which provides a unique way to synthesize functionally substituted cyclobutane rings in a sterically challenging environment.^8^ The plan toward (±)**-4a** involved the construction of a heavily functionalized cyclopentane via sequential conjugate addition and alkylation sequence. The route commenced with β-substituted enone (**6**), available from 1,3-diketone (**5**) via enol ether formation followed by Grignard addition.^4,9^ A Cu-catalyzed conjugate addition of vinyl magnesium bromide to enone **6** in the presence of TMSCl led to the isolation of silyl enol ether (±)**-7** in quantitative yield. The introduction of the nitrile functionality via alkylation of (±)**-7** with bromoacetonitrile^11−13^ via *in situ* generation of the enolate with KO*t*Bu or CsF initially failed, resulting in either recovery of (±)**-7** (CsF) or the corresponding ketone (KO*t*Bu). Lewis acids [Yb(OTf)_3_, InCl_3_, and BF_3_] also failed to promote the alkylation. However, the lithium enolate was readily generated by MeLi at 0 °C, and by subsequent alkylation with bromoacetonitrile at −50 to −30 °C, the desired alkylation product (±)**-8** was obtained in 30% yield and 9:1 dr. Unfortunately, even after extensive changes in the variables, (temperatures, solvents, reaction times, and concentrations), the yield of the alkylation reaction could not be improved, Nevertheless, the addition of MeMgBr to ketone (±)**-8** furnished tertiary alcohol (±)**-9** with excellent diastereoselectivity (>20:1 dr).

**Scheme 2:**
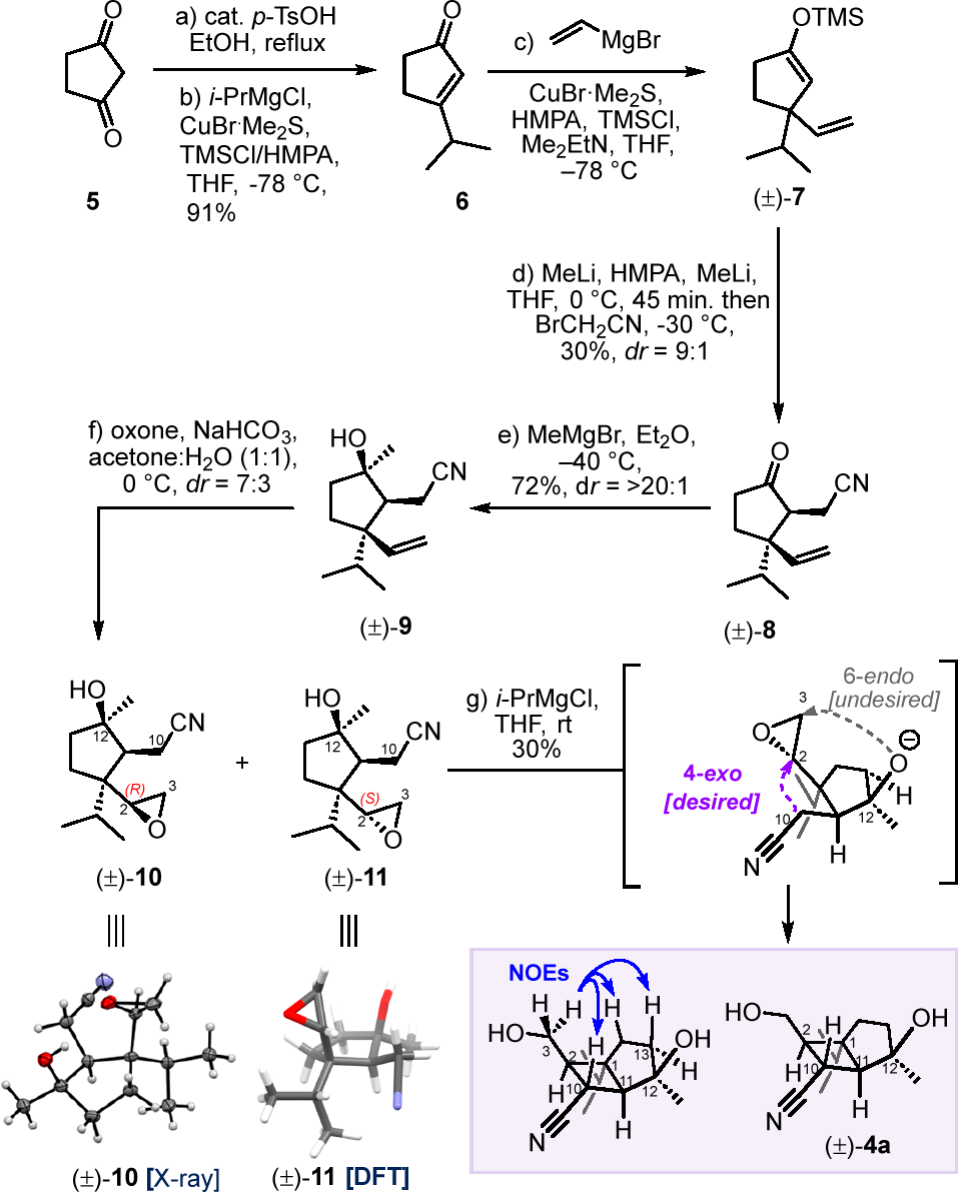
First-Generation Route to the Bicyclo[3.2.0]heptane Core of Humilisin E [(±)**-4a**]

To set the stage for the Stork epoxynitrile cyclization, oxone oxidation of (±)**-9** successfully delivered epoxide as a separable mixture of diastereomers (±)-**10** (43%) and (±)**-11** (27%) (crude 7:3 dr). Unfortunately, (±)-**10**, possessing the undesired relative configuration at C2 (R*, as shown), was the major product. Attempts to improve the diastereoselection by alternative reagents (*m*-CPBA or TBHP) resulted in no reaction, again highlighting the hindered nature of isopropyl-substituted cyclopentane. The relative stereochemistry of (±)**-10** was unambiguously assigned by scXRD (see Scheme 2; CCDC 2310461).

**Figure 2:**
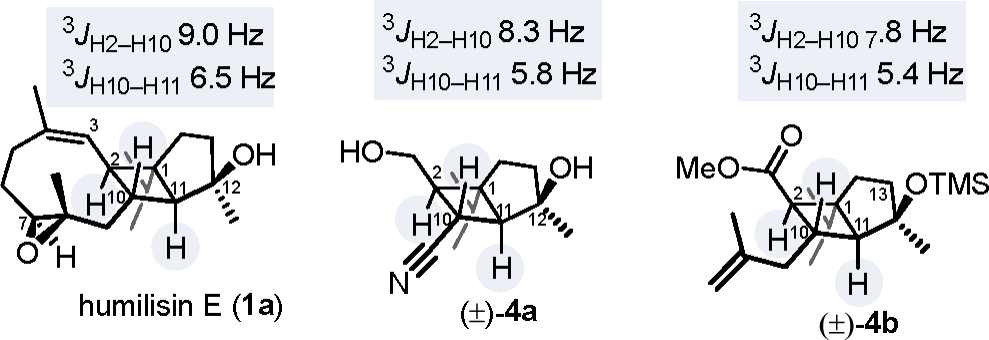
Comparison of ^3^*J*_H–H_ spin–spin coupling constants observed in the ^1^H NMR spectra of the cyclobutane ring of humilisin E (**1a**) and the corresponding bicyclo[3.2.0]heptane cores of **1a**, (±)-**4a** and (±)-**4b**.

Computational analysis of the desired isomer, (±)-**11**, revealed a potential risk in the synthesis plan, which was not anticipated earlier. The epoxy nitrile cyclization with a base might also rapidly deprotonate the tertiary alcohol, resulting in the undesired 6-*endo* (or 5-*exo*) cyclization with the epoxide forming bicyclic ether, as the C12 OH and the epoxide were both pseudoaxially disposed in (±)**-10**. To guard against this liability, attempts were made to protect the tertiary alcohol with a TMS group, but without success. Therefore, to avoid the unwanted cyclization or alkylation of the C12 tertiary alcohol, we followed a literature precedent by Fleming and co-workers, who had used Grignard reagents to metalate nitriles and simultaneously form Mg alkoxides from tertiary alcohols.^14,15^ We hypothesized that the formation of Mg alkoxide would deactivate the tertiary alcohol. Indeed, epoxynitrile (±)**-11** was successfully cyclized to afford cyclobutane (±)-**4a** upon treatment with *i*-PrMgCl in 30% yield. The relative stereochemistry of (±)-**4a** was confirmed by nuclear Overhauser effect (NOE) experiments (Scheme 2) as well as by comparison of the ^1^H NMR coupling constants (see Figure 2). Again, attempts to improve the yield of this step were unsuccessful; with a larger excess of *i*-PrMgCl (6 equiv), addition to the nitrile was also observed, resulting in the formation of the corresponding isopropyl ketone in <25% yield. Decreasing the temperature did not improve the chemoselectivity; no reaction was observed at 0 °C. Prolonged reaction times resulted in decomposition.

**Scheme 3:**
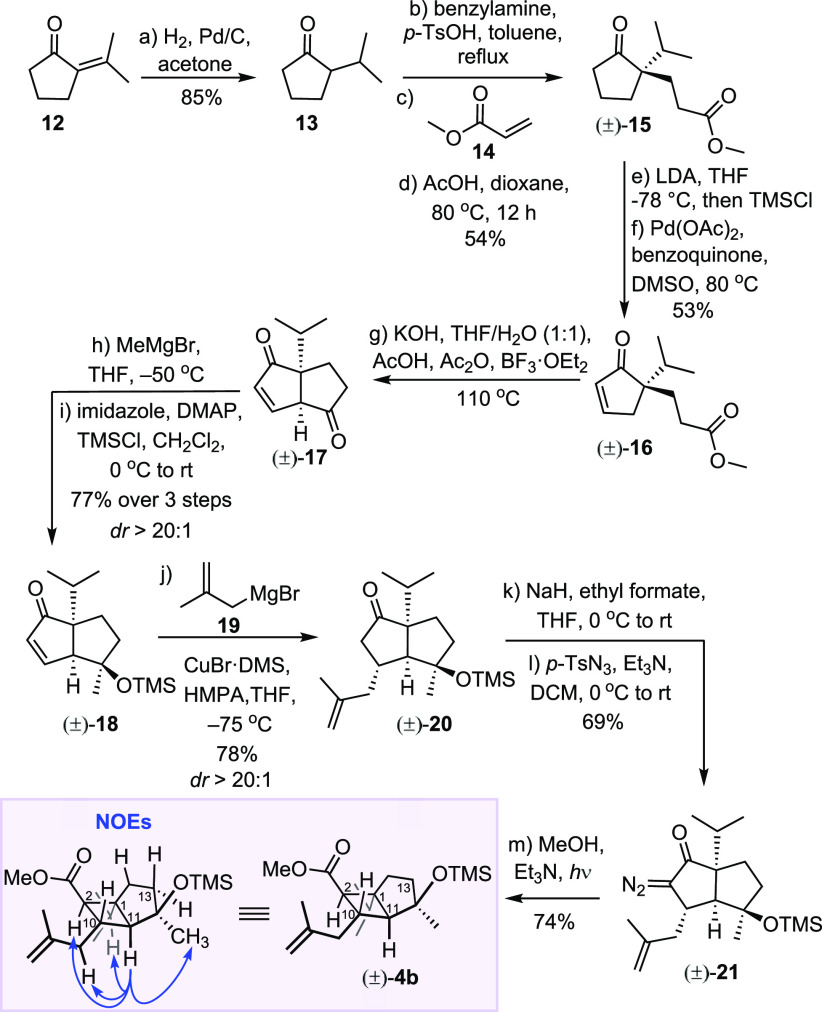
Second-Generation Approach to the Functionalized Bicyclo[3.2.0]heptane Core [(±)**-4b**] of Humilisin E

Although the first-generation synthesis of the bicyclic core delivered products with the desired relative stereochemistry, the route suffered from a number of low-yielding steps and poor stereocontrol. We therefore started over and outlined a second strategy involving Wolff ring contraction to construct an appropriately functionalized cyclobutane ring (Scheme 3). The second strategy was designed to avoid stereocontrol issues by constructing the bicyclic system earlier in the route, followed by ring contraction of the bicyclo[3.3.0]octane ring system to the desired bicyclo[3.2.0]heptane.

The route started with ketone **13**, readily accessible from **12** on a decagram scale.^16^ To set up the quaternary center at C1, we opted for the Pfau–d’Angelo method,^17,18^ and (±)-**15** was obtained in reasonable 54% yield from acrylate **14** and ketone **13** using achiral benzylamine. Alternatively, (−)**-15** was obtained in 64% yield and 98:2 er using (*S*)-(−)-α-methylbenzylamine, enabling the enantioselective synthesis, as well. The route, however, continued with racemic (±)-**15**. Among the conditions screened for the dehydrogenation of ketone (±)-**15** to enone (±)-**16**, including bromination and dehydrobromination,^19,20^ IBX-mediated single-electron transfer oxidation,^21^ or Pd(II)-catalyzed direct dehydrogenation of carbonyl compounds,^22−25^ only Saegusa oxidation^26^ of the silyl enol ether derived from ketone (±)**-15** gave the best results, affording (±)-**16** in 53% yield. Following the precedent set by Tice and Heathcock^27^ for the Lewis acid-catalyzed vinylogous Claisen condensation, cyclization of enone ester (±)**-16** with BF_3_ proceeded readily, affording crude enedione (±)**-17**, which was subjected directly to the diastereoselective addition of MeMgBr (>20:1 dr). After TMS protection, enone (±)**-18** was obtained in a 77% yield over three steps. Setting up the C10 stereocenter involved a Cu-catalyzed conjugate addition of 2-methylallylmagnesium bromide (**19**) to give (±)**-20** in 78% yield (>20:1 dr).^10^ From (±)**-20**, Regitz formyl diazo transfer gave α-diazoketone (±)**-21**.^28,29^ Finally, photolysis of (±)**-21** in anhydrous methanol and Et_3_N led to the formation of cyclobutane (±)**-4b** in 74% yield.^30^

The stereochemistry of (±)**-4b** was confirmed by one-dimensional NOE experiments (Scheme 3; see the Supporting Information for details) and comparison of ^3^*J*_HH_ coupling constants with those of humilisin E and (±)**-4a** (Figure 2). The invariance of the coupling constants among **1a**, (±)**-4a**, and (±)**-4b** suggests that humilisin E and the newly synthesized core fragments (±)**-4a** and (±)**-4b** share the same relative configuration at C2, C10, and C11 and also similar conformational preferences. This might be of assistance in the projected total synthesis of **1a** via closure of the nine-membered ring (cf. Scheme 1).

## Conclusion

In conclusion, we have developed two alternative routes to the functionalized bicyclo[3.2.0]heptane core of humilisin E via either the Stork nitrile epoxide method or Wolff rearrangement.^31^ The asymmetric version of the second route and progress in the total synthesis of **1a** will be reported in due course.

## Data Availability

The data underlying this study are available in the published article and its Supporting Information.

## Supporting Information Available

XYZ coordinates of computed structures **1a**, **2a**, and **11** (ZIP)
FAIR data, including the primary NMR FID files, for compounds **4a**, **4b**, **6–13**, (±)-**15**, (−)-**15**, (±)-**16**, (−)-**16**, **18**, **20**, and **21** (ZIP)
Experimental procedures, characterization data, crystallographic details, and computational details (PDF) Copies of NMR spectra (PDF)
