# Supplementary material for: Humilisin E: Strategy for the Synthesis and Access to the Functionalized Bicyclic Core
Source: J Org Chem. 2024 Apr 26;89(10):6987–90. doi: 10.1021/acs.joc.4c00358 (PMC11110058; doi:10.1021/acs.joc.4c00358)
Supplement: Supplementary file 2 — jo4c00358_si_002.zip [file jo4c00358_si_002.zip › FID for publication/Information_on_data_acquisition.docx]

**Name of the manufacturer of the spectrometer used to collect the data:**

The ^1^H NMR and 13C{1H} NMR spectra were recorded in CDCl_3_, CD_2_Cl_2_ or C_6_D_6_ on Bruker Avance 500 or 300 spectrometers. The chemical shifts are reported in ppm relative to CHCl_3_ (δ 7.26), CDHCl_2_ (δ 5.32) or C_6_D_5_H (7.16) for ^1^H NMR. For the ^13^C NMR spectra, CDCl_3_ (δ 77.16), CD_2_Cl_2_ (δ 53.84) or C_6_D_6_ (128.06) were used as the internal standards*.*

**Acquisition software:** Bruker TopSpin 3.5 pl 7 on 500 MHz and Bruker TopSpin 3.5 pl 6 on NMR 300 MHz.

**Processing programs used to analyse the data:** MestReNova 14.3.0 and Topspin 3.6.2

**The field strength used to measure each nucleus:**

**S2**: **^1^H NMR** 300 MHz, **^13^C NMR** 75 MHz

**6**: **^1^H NMR** 300 MHz; **^13^C NMR** 75 MHz

**7**: **^1^H NMR** 300 MHz; **^13^C NMR** 75 MHz

**8**: **^1^H NMR** 500 MHz; **^13^C NMR** 125 MHz

**9**: **^1^H NMR** 500 MHz; **^13^C NMR** 125 MHz

**10**: **^1^H NMR** 500 MHz; **^13^C NMR** 125 MHz

**11**: **^1^H NMR** 500 MHz; **^13^C NMR** 125 MHz

**4a**: **^1^H NMR** 500 MHz; **^13^C NMR** 125 MHz

**13**: **^1^H NMR** 300 MHz; **^13^C NMR** 75 MHz

**15**: **^1^H NMR** 300 MHz; **^13^C NMR** 75 MHz

**16**: **^1^H NMR** 300 MHz; **^13^C NMR** 75 MHz

**18**: **^1^H NMR** 300 MHz; **^13^C NMR** 75 MHz

**20**: **^1^H NMR** 300 MHz; **^13^C NMR** 75 MHz

**21**: **^1^H NMR** 300 MHz; **^13^C NMR** 75 MHz

**4b**: **^1^H NMR** 300 MHz; **^13^C NMR** 75 MHz
