# Supplementary figures and images for: Humilisin E: Strategy for the Synthesis and Access to the Functionalized Bicyclic Core
Source: J Org Chem. 2024 Apr 26;89(10):6987–90. doi: 10.1021/acs.joc.4c00358 (PMC11110058; doi:10.1021/acs.joc.4c00358)

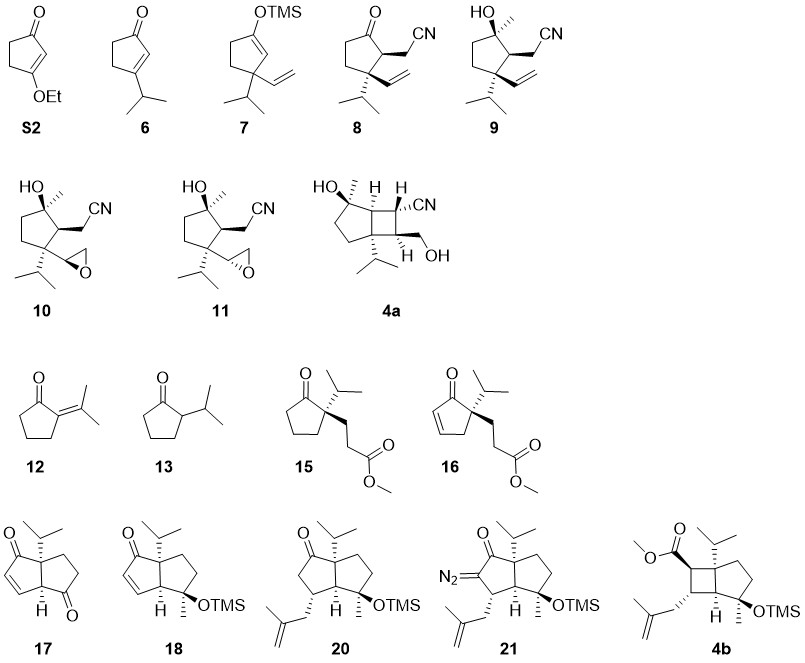

Supplement: Supplementary file 2 — jo4c00358_si_002.zip [file jo4c00358_si_002.zip › FID for publication/HumilisinE_manuscript_metadata_structures.png]
