# Supplementary material for: Humilisin E: Strategy for the Synthesis and Access to the Functionalized Bicyclic Core
Source: J Org Chem. 2024 Apr 26;89(10):6987–90. doi: 10.1021/acs.joc.4c00358 (PMC11110058; doi:10.1021/acs.joc.4c00358)
Supplement: Supplementary file 3 — jo4c00358_si_003.pdf [file jo4c00358_si_003.pdf]

Supporting Information

Humilisin E: Strategy for Synthesis and Access to the Functionalized Bicyclic Core

Prachi Verma,[‡] Rajanish Pallerla,[‡] Aino Rolig, Petri M. Pihko*
Department of Chemistry and Nanoscience Center, University of Jyväskylä, P.O.B. 35, FI-40014 JYU, Jyväskylä, FINLAND
Petri.Pihko@jyu.fi

Contents

Humilisin E: Strategy for Synthesis and Access to the Functionalized Bicyclic Core 1

1 General Information 4

2 Experimental 5

2.1 First approach to the bicyclo[3.2.0]heptane core (±)-4a 5

2.1.1 Synthesis route to 3-isopropylcyclopent-2-en-1-one (**6**) 5

2.1.2 ((3-isopropyl-3-vinylcyclopent-1-en-1-yl)oxy)trimethylsilane ((±)-**7**) 7

2.1.3 2-((1S,2S)-2-isopropyl-5-oxo-2-vinylcyclopentyl)acetonitrile ((±)-**8**) 8

2.1.4 2-((1S,2R,5S)-2-hydroxy-5-isopropyl-2-methyl-5-vinylcyclopentyl)acetonitrile ((±)-**9**) 8

2.1.5 Epoxides (±)-**10** & (±)-**11** 9

2.1.6 (1S,4R,5S,6R,7R)-4-hydroxy-7-(hydroxymethyl)-1-isopropyl-4-methylbicyclo[3.2.0]heptane-6-carbonitrile ((±)**-4a**).................... 10

2.2 Second route to the bicyclo[3.2.0]heptane core (±)-4b 11

2.2.1 2-isopropylcyclopentan-1-one (**13**) 11

2.2.2 Methyl 3-(1-isopropyl-2-oxocyclopentyl)propanoate ((±)-**15**).................... 11

2.2.3 Methyl 3-(1-isopropyl-2-oxocyclopentyl)propanoate ((–)-**15**).................... 12

2.2.4 Methyl 3-(1-isopropyl-2-oxocyclopent-3-en-1-yl)propanoate ((±)-**16**).................... 12

2.2.5 Methyl 3-(1-isopropyl-2-oxocyclopent-3-en-1-yl)propanoate ((–)-**16**).................... 13

2.2.6 6a-isopropyl-4-methyl-4-((trimethylsilyl)oxy)-4,5,6,6a-tetrahydropentalen-1(3aH)-one ((±)-**18**) 14

2.2.7 6a-isopropyl-4-methyl-3-(2-methylallyl)-4-((trimethylsilyl)oxy)hexahydropentalen-1(2H)-one ((±)-**20**).................... 15

2.2.8 2-diazo-6a-isopropyl-4-methyl-3-(2-methylallyl)-4-((trimethylsilyl)oxy)hexahydropentalen-1(2H)-one ((±)-**21**).................... 16

2.2.9 5-isopropyl-2-methyl-7-(2-methylallyl)-2-((trimethylsilyl)oxy)bicyclo[3.2.0]heptane-6-carboxylate ((±)-**4b**) 17

2.3 X-ray data for (±)-10 17

2.4 Comparison of ^{1}H spin-spin coupling constants in (±)-4a and (±)-4b 19

2.5 1-D NOE experiment 20
2.5.1 NOE interactions in (±)-**4a** 20
2.5.2 NOE interactions in (±)-**4b** 21
3 Computational details...23
3.1 Computational procedures 23
3.2 Computational Results 24
3.3 Total energy data 25
3.4 Atomic coordinates of the computed structures..25
4 Copies of NMR spectra and GC chromatograms..26

1 General Information

All reactions were carried out under an argon atmosphere in oven-dried glassware, unless otherwise noted. When needed, nonaqueous reagents were transferred under argon *via* syringe or cannula and dried prior to use. THF, Et_2O, toluene, acetonitrile and DCM were obtained by passing deoxygenated solvents through activated alumina columns (MBraun SPS-800 Series solvent purification system). Vinyl magnesium chloride, iPrMgCl, *n*-BuLi and MeLi were titrated before each use (CAUTION: *n*-BuLi and MeLi are pyrophoric, and their handling requires proper air and moisture sensitive techniques). Other solvents and reagents were used as obtained from supplier, unless otherwise noted. Analytical TLC was performed using Merck silica gel F254 (230-400 mesh) plates and analyzed by UV light or by staining upon heating with $KMnO_4$ solution (1 g $KMnO_4$, 6.7 g K_2CO_3, 1.7 mL 1M NaOH, 100 mL H_2O), vanillin solution (6 g vanillin, 5 mL conc. H_2SO_4, 3 mL glacial acetic acid, 250 mL EtOH). For silica gel chromatography, the flash chromatography technique with Merck silica gel 60 (230-400 mesh) and CombiFlash R_f 200 with RediSep Gold or Silver columns (20–40 µm spherical silica, 400–632 mesh) were used and p.a. grade solvents unless otherwise noted. All solvents were HPLC grade unless otherwise mentioned. All the heating reactions were carried on oil baths.

The ^1H NMR and ^{13}C NMR spectra were recorded in either $CDCl_3$, CD_2Cl_2 or C_6D_6 on Bruker Avance 500 or 300 MHz spectrometers. The chemical shifts are reported in ppm relative to $CHCl_3$ (δ 7.26), $CDHCl_2$ (δ 5.32) or C_6D_5H (7.16) for ^1H NMR. For the ^{13}C NMR spectra, the residual $CDCl_3$ (δ 77.16), CD_2Cl_2 (δ 53.84) or C_6D_6 (δ 128.06) were used as the internal standards. Melting points (mp) were determined in open capillaries using Stuart melting point apparatus. IR spectra were recorded on a Bruker Tensor 27 FT-IR spectrometer. High resolution mass spectrometric data were measured using Agilent Technologies 6560 Ion Mobility ESI-Q-TOF LC/MS. The enantiomeric ratios were determined by gas chromatography using Agilent Technologies gas chromatograph 7820A with Supelco Astec Chiraldex b-DM column (1.8805 mL/min He flow rate).

2 Experimental

2.1 First approach to the bicyclo[3.2.0]heptane core (±)-4a

2.1.1 Synthesis route to 3-isopropylcyclopent-2-en-1-one (**6**)

2.1.1.1 Using intramolecular aldol condensation

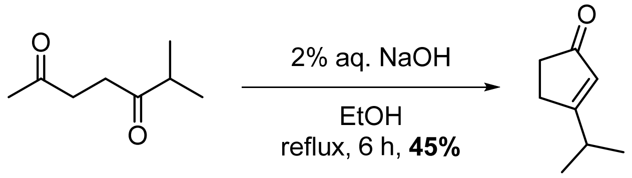

S1 **6**

To a solution of 2% aq. NaOH (5 mL) and ethanol (5 mL) at room temperature, diketone **S1** (0.12 g, 0.86 mmol, 1.00 equiv.) was added and the resulting mixture was heated to reflux for 6 h. The reaction mixture was cooled to rt and diluted with water (10 mL) and Et_2O (10 mL). The phases were separated, and the aqueous phase was extracted with Et_2O (2 x 10 mL). The combined organic layers were washed with brine (20 mL), dried (Na_2SO_4), filtered and concentrated. The residue was purified by flash chromatography (Et_2O – Pentane 40:60) to give 3-isopropylcyclopent-2-en-1-one (**6**) as a yellow oil (0.05 g, 0.38 mmol, 45%). Spectral data matches those reported previously in the literature[1].

R_f (40% EtOAc/hexanes) = 0.50; IR (film, cm^{-1}): 2964, 2931, 1706, 1608, 1175; ^1H NMR (300 MHz, $CDCl_3$): δ 5.88 (d, J = 1.2 Hz, 1H), 2.64 – 2.55 (m, 3H), 2.37 – 2.34 (m, 2H), 1.14 (d, J = 7.0 Hz, 6H); $^{13}C\{^1H\}$ NMR (75 MHz, $CDCl_3$): δ 210.2, 188.5, 127.9, 35.3, 32.2, 29.5, 20.9.

Using Cu-catalyzed conjugate addition

The scheme below (Scheme S1) illustrates our approach towards the synthesis of 3-isopropylcyclopent-2-en-1-one **6** starting from commercially available cyclopentane-1,3-dione **5**.

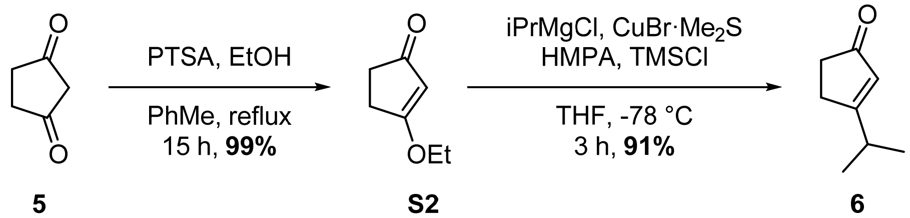

5 **S2** **6**

[1] Fanta, W. I. ja Erman, W. F., Total Synthesis of *dl*-Sabinene, *dl*-trans-Sabinene Hydrate, and Related Monoterpenes, *Journal of Organic Chemistry*, **1968**, *33*, 1656–1658.

Scheme S1: Preparation of 3-isopropylcyclopent-2-en-1-one **6** from cyclopentane-1,3-dione **5.**

2.1.1.2 3-ethoxycyclopent-2-en-1-one (**S2**)

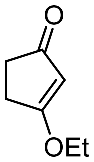

S2

To a stirred slurry of cyclopentane-1,3-dione (**5**) (5.00 g, 51.0 mmol, 1.00 equiv.) in 70 mL toluene at room temperature was added *p*-TsOH (0.49 g, 2.55 mmol, 0.05 equiv.) and ethanol (22.6 mL, 387 mmol, 7.60 equiv.). The resulting mixture was heated to reflux for 15 h. The reaction mixture was cooled to rt and concentrated to remove the volatiles. The residue was purified by flash chromatography (DCM – EtOAc 75:25) to give 3-ethoxycyclopent-2-en-1-one (**S2**) as a colorless oil (6.35 g, 50.3 mmol, 99%). Spectral data matches those reported previously in the literature[2].

R_f (25% EtOAc/DCM) = 0.30; IR (film, cm^{-1}): 2978, 1670, 1560, 1440, 1160; ^{1}H NMR (300 MHz, $CDCl_3$): δ 5.23 (s, 1H), 4.00 (q, *J* = 6.9 Hz, 2H), 2.57 – 2.53 (m, 2H), 2.39 – 2.35 (m, 2H), 1.35 (t, *J* = 7.0 Hz, 3H); ^{13}C{^{1}H} NMR (75 MHz, $CDCl_3$): δ 205.9, 190.1, 104.6, 67.6, 34.0, 28.5, 14.1.

2.1.1.3 3-isopropylcyclopent-2-en-1-one (**6**)

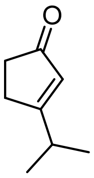

6

To a stirred slurry of CuBr·Me_2S (1.17 g, 5.70 mmol, 0.05 equiv.) in dry THF (50 mL) at -78 °C was added a solution of isopropylmagnesium chloride in THF (2.0 M, 80.0 mL, 159 mmol, 1.40 equiv.) over 20 minutes, followed by HMPA (47.6 mL, 273 mmol, 2.40 equiv.) over 10 minutes. The resulting mixture was then allowed to stir at -78 °C for 15 minutes. A solution containing 3-ethoxycyclopent-2-en-1-one (**S2**) (14.4 g, 114 mmol, 1.00 equiv.), TMSCl (29 mL, 228 mmol, 2.00 equiv.) and dry THF (20 mL) was added to the reaction mixture over 20 minutes. The reaction mixture was allowed to stir at -78 °C for 3 h. Sat. aq. NH_4Cl (20 mL) and Et_2O (20 mL) were added, the phases were separated, and the aqueous phase was extracted with Et_2O (2 x 10 mL). The combined organic layers were washed with brine (10 mL), dried (Na_2SO_4), filtered

[2] Wang, H.; Li, H.; Wang, J. ja Wu, Y., Protecting-group-free synthesis of haterumadienone- and puupehenone-type marine natural products, *Green Chemistry*, **2017**, *19*, 2140–2144.

and concentrated. The residue was purified by flash chromatography (Et_2O – Pentane 40:60) to give 3-isopropylcyclopent-2-en-1-one (**6**) as a yellow oil (12.9 g, 104 mmol, 91%). Spectral data matches those reported previously in the literature[1].

R_f (40% EtOAc/hexanes) = 0.50; IR (film, cm^{-1}): 2964, 2931, 1706, 1608, 1175; ^1H NMR (300 MHz, $CDCl_3$): δ 5.89 (d, *J* = 1.2 Hz, 1H), 2.64 – 2.55 (m, 3H), 2.37 – 2.34 (m, 2H), 1.14 (d, *J* = 7.0 Hz, 6H); $^{13}C\{^1H\}$ NMR (75 MHz, $CDCl_3$): δ 210.2, 188.5, 127.9, 35.3, 32.2, 29.5, 20.9.

2.1.2 ((3-isopropyl-3-vinylcyclopent-1-en-1-yl)oxy)trimethylsilane ((±)-**7**)

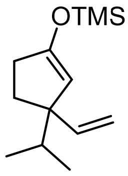

(±)-**7**

To a stirred slurry of CuBr·Me_2S (0.83 g, 4.03 mmol, 0.10 equiv.) in dry THF (20 mL) at -78 °C was added a solution of vinylmagnesium bromide in THF (1 M, 73.0 mL, 72.5 mmol, 1.80 equiv.) over 20 minutes, followed by HMPA (25.0 mL, 145 mmol, 3.60 equiv.) over 10 minutes. The resulting mixture was then allowed to stir at -78 °C for 15 minutes. A solution containing 3-isopropylcyclopent-2-en-1-one (**6**) (5.00 g, 40.3 mmol, 1.00 equiv.), TMSCl (15.3 mL, 121 mmol, 3.00 equiv.) and dry THF (10 mL) was added to the reaction mixture over 20 minutes. The reaction mixture was allowed to stir at -78 °C for 3 h. Me_2EtN (13.1 mL, 121 mmol, 3.00 equiv.) was added and diluted with pentane (100 mL). Resulting mixture was filtered through celite and washed with sat. aq. $NaHCO_3$ (75 mL). The phases were separated, and the organic layer was washed with brine (50 mL), dried (Na_2SO_4), filtered and concentrated. The residue was purified by flash chromatography using pentane as eluent to give enolsilane (±)-**7** as a yellow oil (9.00 g, 40.1 mmol, >99%). Enolsilane (±)-**7** degraded during storage and was used immediately. **NOTE:** (±)-**7** can also be used directly in the next step, after concentration of the extracts.

R_f (10% EtOAc/hexanes) = 0.90; IR (film, cm^{-1}): 2957, 2926, 1249, 1091, 867, 839, 755; ^1H NMR (300 MHz, $CDCl_3$): δ 5.88 (dd, J_1 = 9.9 Hz, J_2 = 17.9 Hz, 1H), 4.95 (dd, J_1 = 1.9 Hz, J_2 = 6.0 Hz, 1H), 4.91 (s, 1H), 4.58 (t, *J* = 1.6 Hz, 1H), 2.34 – 2.13 (m, 2H), 1.85 – 1.57 (m, 3H), 0.84 (d, *J* = 6.8 Hz, 3H), 0.83 (d, *J* = 6.8 Hz, 3H); $^{13}C\{^1H\}$ NMR (75 MHz, $CDCl_3$): δ 154.8, 145.5, 111.1, 107.0, 55.7, 36.8, 33.0, 31.8, 18.4, 18.1, 0.2.

2.1.3 2-((1S,2S)-2-isopropyl-5-oxo-2-vinylcyclopentyl)acetonitrile ((±)-**8**)

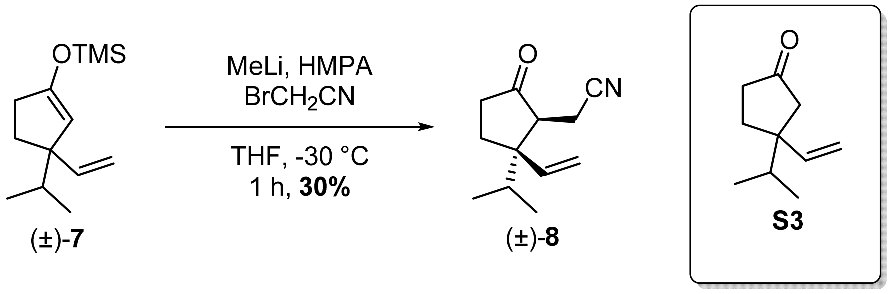

To a stirred solution of enolsilane (±)-**7** (4.00 g, 17.8 mmol, 1.00 equiv.) in dry THF (20 mL) at 0 °C was added a solution of methyl lithium in Et_2O (1.6 M, 15.6 mL, 24.9 mmol, 1.40 equiv.) over 10 minutes. The resulting mixture was then allowed to stir at 0 °C for 45 minutes. The reaction mixture was cooled to -30 °C, HMPA (6.20 mL, 35.6 mmol, 2.00 equiv.) and bromoacetonitrile (2.50 mL, 35.6 mmol, 2.00 equiv.) were added at once. The resulting mixture was let to stir from -30 °C for 1 h. Sat. aq. NH_4Cl (20 mL) and Et_2O (20 mL) were added, the phases were separated, and the aqueous phase was extracted with Et_2O (2 x 20 mL). The combined organic layers were washed with brine (40 mL), dried (Na_2SO_4), filtered and concentrated. The residue was purified by flash chromatography (Et_2O – Pentane 40:60) to give ketonitrile (±)-**8** as a yellow oil (1.0 g, 5.23 mmol, 30%). Ketonitrile (±)-**8** was obtained as a 88:12 mixture of diastereomers with the desired diastereomer as the major product. **NOTE:** The low yield of the reaction was due to the formation of ketone **S3**.

R_f (30% EtOAc/hexanes) = 0.50; IR (film, cm^{-1}): 2305, 1743, 1420, 1264, 732, 703; ^1H NMR (500 MHz, $CDCl_3$): δ 5.57 (ddd, J_1 = 0.9 Hz, J_2 = 11.0 Hz, J_3 = 17.6 Hz, 1H), 5.27 (d, J = 11.0 Hz, 1H), 5.04 (d, J = 17.6 Hz, 1H), 2.71 – 2.66 (m, 2H), 2.42 – 2.35 (m, 1H), 2.26 – 1.99 (m, 5H), 1.90 – 1.83 (m, 1H), 1.04 (d, J = 6.85 Hz, 3H), 0.91 (d, J = 6.85 Hz, 3H); $^{13}C\{^1H\}$ NMR (125 MHz, $CDCl_3$): δ 214.7, 137.9, 118.7, 117.1, 52.4, 51.8, 34.1, 32.0, 23.2, 17.8, 17.0, 12.8; HRMS (ESI^+) m/z: $[M+Na]^+$ calcd for $C_{12}H_{17}NONa^+$ 214.1202; found 214.1203, Δ = 0.5 ppm.

2.1.4 2-((1S,2R,5S)-2-hydroxy-5-isopropyl-2-methyl-5-vinylcyclopentyl)acetonitrile ((±)-**9**)

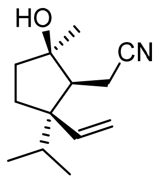

(±)-**9**

To a stirred solution of ketonitrile (±)-**8** (1.00 g, 5.23 mmol, 1.00 equiv.) in dry Et_2O (25 mL) at –40 °C was added a solution of methylmagnesium bromide in Et_2O (3.0 M, 3.50 mL, 10.5 mmol,

2.00 equiv.) over 10 minutes. The resulting mixture was then allowed to stir at –40 °C for 4 h. Sat. aq. $NaHCO_3$ (20 mL) and Et_2O (20 mL) were added, the phases were separated, and the aqueous phase was extracted with Et_2O (2 x 10 mL). The combined organic layers were washed with brine (10 mL), dried (Na_2SO_4), filtered and concentrated. The residue was purified by flash chromatography (Et_2O – Pentane 50:50) to give cyanoalcohol (±)-**9** as a yellow oil (0.78 g, 3.76 mmol, 72%).

R_f (40% EtOAc/hexanes) = 0.50; IR (film, cm^{-1}): 3054, 2967, 2306, 1764, 1381, 1264, 732; ^1H NMR (500 MHz, C_6D_6): δ 5.47 (dd, J_1 = 10.9 Hz, J_2 = 17.6 Hz, 1H), 4.93 (d, J = 10.9 Hz, 1H), 4.85 (d, J = 17.6 Hz, 1H), 2.10 (dd, J_1 = 7.9 Hz, J_2 = 17.0 Hz, 1H), 1.86 (dd, J_1 = 6.4 Hz, J_2 = 17.0 Hz, 1H), 1.68 (t, J = 7.2 Hz, 1H), 1.59 – 1.34 (m, 5H), 1.10 (s, 3H), 0.68 (d, J = 6.8 Hz, 3H), 0.66 (d, J = 6.8 Hz, 3H); $^{13}C\{^1H\}$ NMR (125 MHz, C_6D_6): δ 141.8, 120.2, 114.9, 79.9, 54.1, 51.6, 40.5, 33.3, 28.1, 27.2, 18.4, 17.4, 13.1; HRMS (ESI^+) m/z: $[M+H]^+$ calcd for $C_{13}H_{22}NO^+$ 208.1696; found 208.1690, Δ = -2.9 ppm.

2.1.5 Epoxides (±)-**10** & (±)-**11**

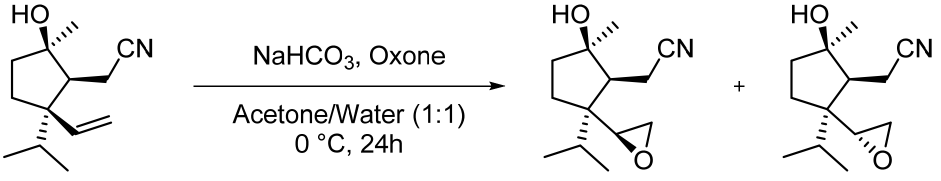

(±)-**9** (±)-**10 (43%)** (±)-**11 (27%)**

To a stirred solution of cyanoalcohol (±)-**9** (0.25 g, 1.23 mmol, 1.00 equiv.) in acetone/water (1:1, 10 mL) at 0 °C was added $NaHCO_3$ (0.50 g, 5.90 mmol, 4.80 equiv.) followed by oxone (0.76 g, 1.23 mmol, 1.00 equiv.). The resulting mixture was then allowed to stir at 0 °C for 24 h. Sat. aq. $Na_2S_2O_3$ (10 mL) and Et_2O (10 mL) were added, the phases were separated, and the aqueous phase was extracted with Et_2O (2 x 10 mL). The combined organic layers were washed with brine (10 mL), dried (Na_2SO_4), filtered and concentrated. The residue was purified by flash chromatography (Et_2O – Pentane 50:50) to give two separable diastereomers with the undesired epoxide (±)-**10** as a white solid (0.12 g, 0.53 mmol, 43%) and the desired epoxide (±)-**11** as a colorless oil (0.07 mg, 0.34 mmol, 27%). mp 100.5 – 101.2 °C;

Undesired Epoxide (±)-**10**:
R_f (30% EtOAc/hexanes) = 0.30; IR (film, cm^{-1}): 3475, 2967, 2303, 1765, 1471, 1091, 952, 708; ^1H NMR (500 MHz, C_6D_6): δ 3.05 (br s, 1H), 2.59 (t, J = 3.5 Hz, 1H), 2.28 (dd, J_1 = 9.9 Hz, J_2 = 17.4 Hz, 1H), 2.15 (dd, J_1 = 5.4 Hz, J_2 = 17.4 Hz, 1H), 2.12 (t, J = 4.2 Hz, 1H), 1.99 (t, J = 3.8 Hz, 1H), 1.82 – 1.75 (m, 2H), 1.44 (dd, J_1 = 7.7 Hz, J_2 = 12.8 Hz, 1H), 1.11 (s, 3H), 1.07 (dd, J_1 = 4.3 Hz, J_2 = 12.8 Hz, 1H), 0.75 (dd, J_1 = 7.9 Hz, J_2 = 13.8 Hz, 1H), 0.57 (d, J = 6.8 Hz, 3H), 0.53 (dd, J_1

= 7.6 Hz, J_2 = 13.6 Hz, 1H), 0.50 (d, J = 6.8 Hz, 3H); $^{13}C\{^1H\}$ NMR (125 MHz, C_6D_6): δ 120.2, 78.7, 54.2, 51.3, 50.0, 46.3, 39.9, 34.1, 24.3, 20.5, 18.6, 17.6, 12.9; HRMS (ESI$^+$) m/z: [M+H]$^+$ calcd for $C_{13}H_{22}NO_2^+$ 224.1645; found 224.1643, Δ = -0.9 ppm.

Desired Epoxide (±)-**11**:
R_f (30% EtOAc/hexanes) = 0.15; IR (film, cm^{-1}): 3490, 2965, 2879, 2303, 1749, 952, 707, 677; ^1H NMR (500 MHz, CD_2Cl_2): δ 2.87 (dd, J_1 = 3.0 Hz, J_2 = 4.0 Hz, 1H), 2.71 (dd, J_1 = 2.9 Hz, J_2 = 4.7 Hz, 1H), 2.63 (t, J = 4.2 Hz, 1H), 2.55 (dd, J_1 = 7.7 Hz, J_2 = 17.1 Hz, 1H), 2.48 (dd, J_1 = 7.2 Hz, J_2 = 17.1 Hz, 1H), 2.00 (t, J = 7.4 Hz, 1H), 1.84 (h, J = 6.8 Hz, 1H), 1.61 – 1.58 (m, 2H), 1.45 (s, 1H), 1.38 (s, 3H), 1.31 – 1.17 (m, 2H), 1.02 (d, J = 6.8 Hz, 3H), 0.96 (d, J = 6.8 Hz, 3H); $^{13}C\{^1H\}$ NMR (125 MHz, CD_2Cl_2): δ 120.7, 80.4, 53.7, 51.0, 50.2, 43.6, 41.0, 37.3, 27.1, 24.0, 18.4, 18.3, 13.1; HRMS (ESI$^-$) m/z: [M-H]$^-$ calcd for $C_{13}H_{20}NO_2^-$ 222.1499, found 222.1486, Δ = -5.8 ppm.

2.1.6 (1S,4R,5S,6R,7R)-4-hydroxy-7-(hydroxymethyl)-1-isopropyl-4-methylbicyclo[3.2.0]heptane-6-carbonitrile ((±)-**4a**)

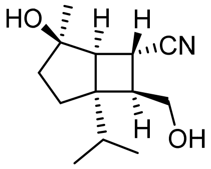

(±)-**4a**

To a stirred solution of desired epoxide (±)-**11** (0.05 g, 0.22 mmol, 1.00 equiv.) in dry THF (5 mL) at 0 °C was added a solution of isopropylmagnesium chloride in THF (2.0 M, 0.35 mL, 0.67 mmol, 3.00 equiv.). Ice bath was removed, and the resulting mixture was then allowed to stir at rt for 3 h. Sat. aq. $NaHCO_3$ (5 mL) and Et_2O (10 mL) were added, the phases were separated, and the aqueous phase was extracted with Et_2O (2 x 10 mL). The combined organic layers were washed with brine (10 mL), dried (Na_2SO_4), filtered and concentrated. The residue was purified by flash chromatography (Et_2O – Pentane 50:50) to give cyanodiol (±)-**4a** as a yellow oil (0.15 g, 0.67 mmol, 30%).

R_f (50% EtOAc/hexanes) = 0.30; IR (film, cm^{-1}): 3400, 2959, 2874, 2235, 1466, 1387, 952, 708, 677; ^1H NMR (500 MHz, CD_2Cl_2): δ 3.74 – 3.66 (m, 2H), 2.83 (dd, J_1 = 5.9 Hz, J_2 = 8.4 Hz, 1H), 2.59 (dd, J_1 = 7.7 Hz, J_2 = 15.2 Hz, 1H), 2.35 (d, J = 5.9 Hz, 1H), 1.94 – 1.87 (m, 1H), 1.75 – 1.65 (m, 3H), 1.43 (br s, 1H), 1.41 – 1.36 (m, 1H), 1.28 (s, 3H), 0.96 (d, J = 6.8 Hz, 3H), 0.83 (d, J = 6.8 Hz, 3H); $^{13}C\{^1H\}$ NMR (125 MHz, CD_2Cl_2): δ 123.0, 78.3, 62.6, 53.7, 52.9, 44.1, 39.8, 37.5, 28.6, 27.7, 20.9, 18.2, 17.3; HRMS (ESI$^+$) m/z: [M+Na]$^+$ calcd for $C_{13}H_{21}O_2NNa^+$ 246.1465; found 246.1459, Δ = -2.4 ppm.

2.2 Second route to the bicyclo[3.2.0]heptane core (±)-4b

2.2.1 2-isopropylcyclopentan-1-one (**13**)

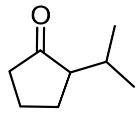

13

2-(propan-2-ylidene)cyclopentan-1-one (**12**)[3] (10.0 g, 80.53 mmol, 1.00 equiv.) was dissolved in acetone (50 mL) and Pd/C (0.50 g, 5 wt% Pd/C) was added to it. The reaction was stirred for 12 h under hydrogen atmosphere at balloon pressure. The completion of the reaction was monitored by TLC. After completion the reaction mixture was filtered through a celite pad, concentrated under reduced pressure to obtain **13** as a colourless oil (8.17 g, 68.94 mmol, 85%). The compound was used in the next step without further purification.

R_f (10% EtOAc/hexane) = 0.5; IR (film, cm^{-1}): 2959, 2338, 1467, 1151, 826; ^{1}H NMR (300 MHz, C_6D_6): δ 2.31 – 2.01 (m, 1H), 2.01 – 1.87 (m, 1H), 1.76 – 1.37 (m, 4H), 1.20 (dt, J_1 = 8.8, J_2 = 5.9 Hz, 2H), 0.94 – 0.84 (d, J = 6.9, 3H), 0.81 – 0.70 (d J = 6.8, 3H); ^{13}C{^{1}H} NMR (75 MHz, C_6D_6): δ 217.5, 54.4, 38.4, 27.3, 24.5, 20.7, 20.3, 18.4.

2.2.2 Methyl 3-(1-isopropyl-2-oxocyclopentyl)propanoate ((±)-**15**)

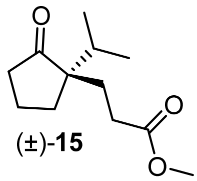

(±)-**15**

A solution of 2-isopropylcyclopentan-1-one (**13**) (1.00 g, 7.92 mmol, 1.00 equiv.), benzylamine (0.87 mL, 7.92 mmol, 1.00 equiv.) and *p*-toluenesulfonic acid (13.6 mg, 0.08 mmol, 0.01 equiv.) in toluene (20 mL) was refluxed with a Dean-Stark apparatus for 7 h. The solvent was then removed under reduced pressure and methyl acrylate (10.77 mL, 118 mmol, 15.0 equiv.) was added. The reaction mixture was further stirred at room temperature for 12 h. The completion of the reaction was monitored by TLC. A 10% aqueous acetic acid solution (60 mL) and 20 mL of 1,4-dioxane was added, and the solution was heated at 80 °C for 12 h. The reaction mixture was extracted with Et_2O (3 × 150 mL), the organic extracts were combined, washed with saturated $NaHCO_3$ (2 × 100 mL), dried (Na_2SO_4), filtered and concentrated under reduced

[3] Berthelot, P.; Vaccher, C.; Devergnies, M.; Flouquet, N. ja Debaert, M., Synthesis and studies of piperidinone, the major metabolite of a new antidepressant drug S 3344, *J. Heterocyclic Chem.*, **1988**, *25*, 1525-1529.

pressure. The crude product was purified by flash chromatography on silica gel (EtOAc – hexanes 20:80) to obtain (±)-**15** as a pale-yellow oil (0.92 g, 4.33 mmol, 54%).

R_f (20% EtOAc/hexane) = 0.6; IR (film, cm^{-1}): 2959, 1728, 1370, 1169, 997; ^{1}H NMR (300 MHz, C_6D_6): δ 3.35 (s, 3H), 2.37 (ddd, J_1 = 16.3, J_2 = 9.9, J_3 = 6.4 Hz, 1H), 2.18 (ddd, J_1 = 16.3, J_2 = 9.6, J_3 = 6.8 Hz, 1H), 2.02 – 1.78 (m, 2H), 1.77 – 1.62 (m, 3H), 1.52 (dd, J_1 = 12.8, J_2 = 8.2 Hz, 1H), 1.42 – 1.27 (m, 2H), 1.22 – 1.05 (m, 1H), 0.62 (d, J = 6.8 Hz, 6H); ^{13}C{^{1}H} NMR (75 MHz, C_6D_6): δ 220.2, 173.0, 53.3, 50.7, 38.6, 29.6, 29.5, 28.9, 28.3, 18.4, 17.8, 16.6; HRMS (ESI$^+$) m/z: [M+Na]$^+$ calcd for $C_{12}H_{20}O_3Na^+$ 235.1305; found 235.1305, Δ = 0.8 ppm.

2.2.3 Methyl 3-(1-isopropyl-2-oxocyclopentyl)propanoate ((–)-**15**)

A solution of 2-isopropylcyclopentan-1-one (**13**) (3.00 g, 23.77 mmol, 1.00 equiv.), ((*S*)-(–)-α-Methylbenzylamine (3.68 mL, 28.53 mmol, 1.20 equiv.) and *p*-toluenesulfonic acid (41 mg, 0.23 mmol, 0.01 equiv.) in toluene (20 mL) was refluxed with a Dean-Stark apparatus for 7 h. The solvent was then removed under reduced pressure and methyl acrylate (32.31 mL, 356 mmol, 15.0 equiv.) was added. The reaction mixture was further stirred at room temperature for 12 h. The completion of the reaction was monitored by GC. A 10% aqueous acetic acid solution (80 mL) and 20 mL of 1,4-dioxane was added, and the solution was heated at 80 °C for 12 h. The reaction mixture was extracted with Et_2O (3 × 150 mL), the organic extracts were combined, washed with saturated $NaHCO_3$ (2 × 100 mL), dried (Na_2SO_4), filtered and concentrated under reduced pressure. The crude product was purified by flash chromatography on silica gel (EtOAc – hexanes 20:80) to obtain (–)-**15** as a pale-yellow oil (3.22 g, 15.17 mmol, 64%).

R_f (20% EtOAc/hexane) = 0.6; $[\alpha]_D$ = –49.0 (*c* 1.00, MeOH, 98:2 *er*)); ^{1}H NMR (300 MHz, $CDCl_3$) δ 3.65 (s, 3H), δ 2.45 – 2.31 (m, 1H), 2.31 – 2.21 (m, 2H), 2.19 – 2.00 (m, 2H), 1.97 – 1.83 (m, 3H), 1.79 (dt, J = 11.0, 5.5 Hz, 2H), 1.62 (dt, J = 12.4, 5.8 Hz, 1H), 0.85 (d, J = 6.9 Hz, 3H), 0.79 (d, J = 6.7 Hz, 3H). The enantiomeric ratio of (–)-**15** was determined after oxidation to the corresponding enone (–)-**16** (see below).

2.2.4 Methyl 3-(1-isopropyl-2-oxocyclopent-3-en-1-yl)propanoate ((±)-**16**)

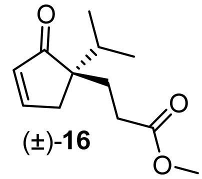

(±)-**16**

To a stirred solution of (±)-**15** (2.00 g, 9.42 mmol, 1.00 equiv.) in THF (25 mL) was added TMSCl (1.79 mL, 14.13 mmol, 1.50 equiv.) under argon atmosphere. The reaction mixture was cooled

to -78 °C and LDA (11.31 mL, 1.00 M, 1.20 equiv.) was added dropwise. The reaction was allowed to stir at -78 °C for 1 h. The completion of reaction was monitored by TLC. After completion the reaction was quenched with sat. aq. $NaHCO_3$, extracted with Et_2O (2 × 20 mL), the organic extracts were combined, dried (Na_2SO_4), filtered, and concentrated under reduced pressure to give corresponding silyl-enol ether as a yellow oil.

The crude was added to the homogeneous solution of $Pd(OAc)_2$ (0.43 g, 1.88 mmol, 0.20 equiv.) and benzoquinone (0.20 g, 1.88 mmol, 0.20 equiv.) in DMSO (20 mL) and heated at 80 °C for 6 h. After completion the reaction mixture was diluted with Et_2O (50 mL) and filtered off through a pad of celite. The organic layer was washed with cold water (2 × 50 mL), dried (Na_2SO_4), filtered, and concentrated under reduced pressure. The crude product was purified by flash chromatography on silica gel (EtOAc – hexanes 20:80) to obtain (±)-**16** as a pale-yellow oil (1.05 g, 4.99 mmol, 53%).

R_f (20% EtOAc/hexane) = 0.6; IR (film, cm^{-1}): 2960, 1735, 1696, 1198, 815; ^1H NMR (300 MHz, $CDCl_3$): δ 7.69 (dt, J_1 = 5.6, J_2 = 2.7 Hz, 1H), 6.14 (dt, J_1 = 5.8, J_2 =2.2 Hz, 1H), 3.64 (s, 3H), 2.64 (dt, J_1 = 19.7, J_2 = 2.5 Hz, 1H), 2.33 – 2.09 (m, 3H), 2.05 – 1.77 (m, 3H), 0.92 (d, J = 6.9 Hz, 3H), 0.72 (d, J = 6.8 Hz, 3H); $^{13}C\{^1H\}$ NMR (75 MHz, $CDCl_3$): δ 214.0, 173.7, 163.5, 134.7, 52.4, 51.5, 36.9, 32.3, 30.8, 28.6, 17.4, 17.3; HRMS (ESI^+) m/z: $[M+Na]^+$ calcd for $C_{12}H_{18}O_3Na^+$ 233.1148; found 233.1146, Δ = –0.8 ppm.

2.2.5 Methyl 3-(1-isopropyl-2-oxocyclopent-3-en-1-yl)propanoate ((–)-**16**)

To a suspension of $Pd(OAc)_2$ (21.0 mg, 0.09 mmol, 0.2 equiv.) in dry DMSO (4,7 ml) was added trifluoroacetic acid (36.0 µl, 0.47 mmol, 1 equiv.) at rt. The resulting suspension was bubbled with oxygen for one minute. Then, methyl 3-(1-isopropyl-2-oxocyclopentyl)propanoate ((–)-**15**) (0.1 g, 0.47 mmol, 1 equiv.) was added. The resulting mixture was left to stir under oxygen at 80 ℃ for 2 days. The reaction mixture was then filtered through a pad of celite with diethyl ether (2x10 ml). The organic phase was washed with cold water (2x15 ml), dried (Na_2SO_4) and concentrated under reduced pressure. The crude product was purified by flash chromatography on silica gel (EtOAc – hexanes 20:80) to obtain (–)-**16** as a pale-yellow oil (23 mg, 0.11 mmol, 23%, *er* 98:2). The enantiomeric ratio was determined by GC. GC (SUPELCO Astec CHIRALDEX B-DM column, 120 ℃ isothermic) t_r = 27.9 min (minor, (*R*)-enantiomer), t_r = 28.5 min (major, (*S*)-enantiomer). The assignment of absolute stereochemistry was done as an analogy based on previously reported research.[4,5] Using Pfau-d'Angelo's[6] method for a similar

[4] Schäfer, C., Michel M., and Miesch, L. *Org. Biomol. Chem.*, **2012**, *10*, 3253-3257.
[5] Horinouchi, R., Kamei, K., Watanabe, R., Hieda, N., Tatsumi, N., Nakano, K., Ichikawa, Y., and Kotsuki H. *Eur. J. Org. Chem.* **2015**, 4457–4463

substrate as **13** and ((*S*)-(−)-α-methylbenzylamine, they report the absolute stereochemistry for the product as R. Based on this, the absolute stereochemistry of (–)-**15** is R and therefore the absolute stereochemistry of (–)-**16** must be S.

R_f (20% EtOAc/hexane) = 0.6; $[\alpha]_D$ = –32.0 (*c* 0.41, MeOH, 98:2 *er*); ^{1}H NMR (300 MHz, $CDCl_3$): δ 7.70 (dt, J_1 = 5.7, J_2 = 2.5 Hz, 1H), 6.14 (m, 1H), 3.64, (s, 3H), 2.65 (dt, J_1 = 19.9, J_2 = 2.0 Hz, 1H), 2.26 (dt, J_1 = 19.8, J_2 = 2.3 Hz, 1H), 2.20-2.15 (m, 1H), 2.00-1.84 (m, 2H), 0.92 (d, *J* = 6.9 Hz, 3H), 0.71 (d, *J* = 6.7 Hz, 3H)

2.2.6 6a-isopropyl-4-methyl-4-((trimethylsilyl)oxy)-4,5,6,6a-tetrahydropentalen-1(3aH)-one ((±)-**18**)

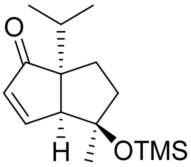

(±)-**18**

To a stirred solution of (±)-**16** (2.00 g, 9.51 mmol, 1.00 equiv.) in THF:H_2O (1:1) (20 mL) was added KOH (0.64 g, 11.41 mmol, 1.20 equiv.) and the reaction was heated at 60 °C for 3 h. The reaction was acidified with 2M HCl solution until the pH was 3. The aqueous layer was extracted with EtOAc (2 × 25 mL) washed with cold water (2 × 10 mL), dried (Na_2SO_4), filtered and concentrated under reduced pressure to obtain the corresponding acid.

The crude was further dissolved in 10 mL acetic acid and acetic anhydride (1.80 mL, 19.02 mmol, 2.00 equiv.) was added under argon atmosphere. The reaction mixture was refluxed for 30 min. followed by the addition of boron trifluoride etherate (1.76 mL, 14.27 mmol, 1.50 equiv.). Refluxing was continued for another 1 h, after which the reaction mixture was poured into 100 mL of cold sat. aq. $NaHCO_3$ and allowed to stir for 1 h. The solution was extracted with Et_2O (2 × 100 mL), and the combined organic extracts were washed with brine (20 mL) dried (Na_2SO_4), filtered and concentrated under reduced pressure. The crude was used in the next step without further purification.

Methyl magnesium bromide (3.0 M, 3.50 mL, 10.49 mmol, 1.10 equiv.) was added dropwise to a stirring solution of diketone (±)-**17** (1.70 g, 9.54 mmol, 1.00 equiv.) in 25 mL of dry THF at -50 °C. The reaction mixture was stirred for 1 h. The completion of the reaction was monitored by TLC. The reaction mixture was then diluted with Et_2O (20 mL) and sat. aq. NH_4Cl (15 mL) was added to it. The residue was extracted with Et_2O (2 × 25 mL), whereupon the organic layer

[6] Pfau, M., Revial, G., Guingant, A. and d'Angelo, J. *J. Am. Chem. Soc.* **1985**, *107*, 214-216.

was washed with brine (20 mL), dried (Na_2SO_4), filtered and evaporated leaving 1.75 g of crude oil. The crude was used in the next step without further purification.
The crude product obtained above (1.75 g, 9.01 mmol, 1.00 equiv.) was dissolved in dry DCM (25 mL) at 0°C. Imidazole (0.61 g, 9.01 mmol, 1.00 equiv.), DMAP (0.01 g, 0.09 mmol, 0.01 equiv.) and TMSCl (1.71 mL, 13.51 mmol, 1.50 equiv.) were added. After 1 h, sat. aq. Na_2CO_3 (20 mL) was added, and the reaction mixture was allowed to stir for 5 minutes. The aqueous residue was extracted with DCM (2 × 20 mL), dried (Na_2SO_4), filtered and concentrated under reduced pressure. Column chromatography on silica gel (Et_2O – pentane 5:95) gave compound (±)-**18** as a colourless oil (1.96 g, 7.32 mmol, 77% over three steps).

R_f (5% EtOAc/hexane) = 0.6; IR (film, cm^{-1}): 2958, 1704, 1250, 1026, 859; ^1H NMR (300 MHz, C_6D_6): δ 7.36 (dd, J_1 = 5.8, J_2 = 3.1 Hz, 1H), 6.04 (dd, J_1 = 5.8, J_2 =1 .7 Hz, 1H), 2.45 (s, 1H), 1.97 – 1.86 (m, 1H), 1.66 (p, *J* = 6.8 Hz, 1H), 1.52 – 1.41 (m, 1H), 1.36 – 1.22 (m, 2H), 1.11 (s, 3H), 1.01 (d, *J* = 6.8 Hz, 3H), 0.76 (d, *J* = 6.9 Hz, 3H), 0.05 (s, 9H); $^{13}C\{^1H\}$ NMR (75 MHz, C_6D_6): δ 213.0, 164.9, 134.8, 81.4, 61.1, 59.4, 38.0, 34.5, 30.7, 29.1, 18.7, 17.7, 2.2; HRMS (ESI^+) m/z: $[M+Na]^+$ calcd for $C_{15}H_{26}O_2SiNa^+$ 289.1594, found 289.1610; Δ = 5.5 ppm.

2.2.7 6a-isopropyl-4-methyl-3-(2-methylallyl)-4-((trimethylsilyl)oxy)hexahydropentalen-1(2H)-one ((±)-**20**)

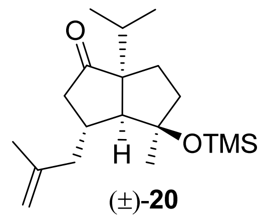

(±)-**20**

To a two-neck round bottom flask flushed with argon, CuBr·Me_2S (0.15 g, 0.75 mmol, 0.20 equiv.) was added and dried under high vacuum. Dry THF (25 mL) was added and the resulting suspension was cooled to -78 °C. 2-Methylallylmagnesium bromide (11.26 mL, 0.5 M, 5.63 mmol, 1.10 equiv.) was added dropwise and stirred for 15 minutes at -78 °C. Enone (±)-**18** (1.00 g, 3.75 mmol, 1.00 equiv.) and HMPA (1.03 mL, 5.63 mmol, 1.50 equiv.) in 10 mL THF were added dropwise to the reaction mixture at -78 °C and the reaction was allowed to stir for 1 h. The completion of reaction was monitored by TLC. After completion the reaction mixture was brought to room temperature, diluted with pentane (10 mL) and sat. aq. NH_4Cl (15 mL) was added to it. The aqueous residue was extracted with Et_2O (2 × 20 mL), dried (Na_2SO_4), filtered and concentrated under reduced pressure. Column chromatography on silica gel (Et_2O – pentane 5:95) gave ketone (±)-**20** as a colourless oil (0.95 g, 2.95 mmol, 78%).

R_f (5% EtOAc/hexane) = 0.6; IR (film, cm^{-1}): 2959, 1732, 1249, 895; ^1H NMR (300 MHz, C_6D_6): δ 4.77 (d, *J* = 17.9 Hz, 2H), 2.47 (dd, J_1 = 17.8, J_2 = 8.9 Hz, 1H), 2.35 – 2.22 (m, 1H), 2.22 – 2.07 (m, 2H), 2.07 – 1.91 (m, 2H), 1.78 (d, *J* = 2.8 Hz, 1H), 1.65 (s, 3H), 1.64 – 1.53 (m, 2H), 1.51 – 1.35 (m, 1H), 1.34 – 1.17 (m, 1H), 1.11 (s, 3H), 0.91 (d, *J* = 6.8, 3H), 0.84 (d, *J* = 6.6 Hz, 3H), 0.09 (s, 9H); $^{13}C\{^1H\}$ NMR (75 MHz, C_6D_6): δ 218.7, 144.5, 112.2, 84.1, 63.5, 60.6, 46.8, 44.8, 41.0, 32.7, 30.0, 27.1, 26.1, 22.1, 18.7, 17.6, 2.2; HRMS (ESI^+) m/z: $[M+Na]^+$ calcd for $C_{19}H_{34}O_2SiNa^+$ 345.2220; found 345.2233, Δ = 3.7 ppm.

2.2.8 2-diazo-6a-isopropyl-4-methyl-3-(2-methylallyl)-4-((trimethylsilyl)oxy)hexahydropentalen-1(2H)-one ((±)-**21**)

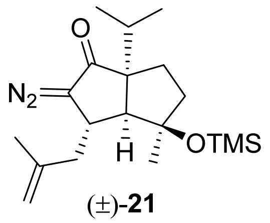

(±)-**21**

A solution of ketone ((±)-**20**) (0.95 g, 2.95 mmol, 1.00 equiv.) in 15 mL dry Et_2O was added dropwise to a stirring solution of sodium hydride (0.17 g, 4.42 mmol, 1.50 equiv.) in dry Et_2O (5 mL) at 0 °C. After 30 minutes freshly distilled ethyl formate (0.36 mL, 4.42 mmol, 1.50 equiv.) was added, and the reaction was allowed to stir for 3 h. The completion of the reaction was monitored by NMR. After completion the reaction mixture was diluted with pentane (10 mL) and sat. aq. NH_4Cl solution (15 mL) was added. The aqueous residue was extracted with Et_2O (2 × 20 mL), dried (Na_2SO_4), filtered and concentrated under reduced pressure leaving 1.00 g of crude oil. The crude was used in the next step without further purification.

Obtained crude oil (1.00 g, 2.85 mmol, 1.00 equiv.) was dissolved in dry DCM (25 mL) at 0 °C. Then, *p*-toluenesulfonyl azide (0.62 g, 3.14 mmol, 1.10 equiv.) and triethyl amine (0.80 mL, 5.70 mmol, 2.00 equiv.) were added. After 1 h, the reaction mixture was concentrated under reduced pressure. Column chromatography on silica gel (Et_2O – pentane 10:90) gave diaza ketone (±)-**21** as a yellow oil (0.69 g, 1.98 mmol, 69% over two steps).

R_f (5% EtOAc/hexane) = 0.6; IR (film, cm^{-1}): 2959, 2070, 0665, 1296, 838; ^1H NMR (300 MHz, C_6D_6): δ 4.78 (d, *J* = 5.4 Hz, 2H), 3.45 (td, J_1 = 8.0, J_2 = 1.8 Hz, 1H), 2.30 – 2.08 (m, 3H), 1.78 (p, *J* = 6.8 Hz, 1H), 1.70 (d, *J* = 1.8 Hz, 1H), 1.63 (s, 3H), 1.59 – 1.44 (m, 2H), 1.28 – 1.19 (m, 1H), 1.08 (s, 3H), 1.03 (d, *J* = 6.8 Hz, 3H), 0.90 (d, *J* = 6.8 Hz, 3H), 0.07 (s, 9H); $^{13}C\{^1H\}$ NMR (75 MHz, C_6D_6): δ 203.1, 143.1, 113.8, 83.2, 64.8, 58.2, 44.8, 40.9, 34.6, 33.6, 29.72, 26.9, 22.0, 18.9, 18.4, 2.1; HRMS (ESI^+) m/z: $[M+Na]^+$ calcd for $C_{19}H_{32}N_2O_2SiNa^+$ 371.2125; found 371.2128, Δ = 0.8 ppm.

2.2.9 5-isopropyl-2-methyl-7-(2-methylallyl)-2-((trimethylsilyl)oxy)bicyclo[3.2.0]heptane-6-carboxylate ((±)-**4b**)

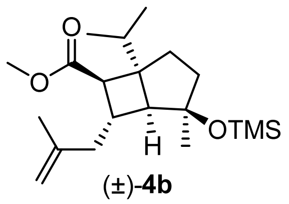

(±)-**4b**

A solution of α-diazoketone (±)-**21** (0.10 g, 0.28 mmol, 1.00 equiv.) in 3 mL dry MeOH was taken in a quartz vessel and triethyl amine (41.0 µL, 0.28 mmol, 1.00 equiv.) was added to it. The reaction mixture was degassed and irradiated at 254 nm for 24 h. The completion of the reaction was monitored by TLC. After completion the solvent was removed under reduced pressure and the crude obtained was purified by column chromatography on silica gel (Et_2O – pentane 5:95) gave compound (±)-**4b** as a colourless oil (0.07 g, 0.21 mmol, 74%).

R_f (5% EtOAc/hexane) = 0.7; IR (film, cm^{-1}): 2961, 1730, 1260, 869; ^1H NMR (300 MHz, C_6D_6): δ 4.84 (d, *J* = 19.9 Hz, 2H), 3.34 (s, 3H), 3.05 (ddd, J_1 = 13.2, J_2 = 10.6, J_3 = 5.7 Hz, 1H), 2.65 (d, *J* = 7.8 Hz, 1H), 2.48 (td, J_1 = 12.6, J_2 = 7.6 Hz, 1H), 2.32 (dd, J_1 = 13.6, J_2 = 5.8 Hz, 1H), 2.06 (dd, J_1 = 13.6, J_2 = 10.3 Hz, 1H), 1.83 (s, 3H), 1.74 (d, *J* = 5.3 Hz, 1H), 1.57 (tt, J_1 = 13.2, J_2= 6.5 Hz, 3H), 1.25 (td, J_1 = 13.6, J_2 = 7.4 Hz, 1H), 1.16 (s, 3H), 0.97 (d, *J* = 6.7 Hz, 3H), 0.84 (d, *J* = 6.8 Hz, 3H), 0.22 (s, 9H); $^{13}C\{^1H\}$ NMR (75 MHz, C_6D_6): δ 173.5, 144.1, 111.7, 81.9, 55.8, 51.6, 50.8, 47.2, 45.4, 39.7, 38.07, 30.1, 29.7, 28.9, 22.4, 18.7, 17.3, 2.5; HRMS (ESI^+) m/z: $[M+K]^+$ calcd for $C_{20}H_{36}O_3SiK^+$ 391.2065; found 391.2066, Δ = 0.2 ppm.

2.3 X-ray data for (±)-10

The X-ray structure of epoxide (±)-**10** was determined using the instrument Rigaku XtaLAB Synergy R diffractometer equipped with Hypix-Arc 100 HPC detector using monochromatized CuKα (λ = 1.54184 Å) radiation. Crystals were obtained by slow evaporation of a solution of (±)-**10** in Et_2O-pentane. The crystals were suspended in protective oil and were mounted on MiTeGen loop for measurement. The data reduction and absorption corrections were made by program CrysAlisPro.[7] The structures were solved by using SHELXT[8] in OleX 2-1.5[9] and refined with SHELXL.[10] All non-hydrogen atoms were refined anisotropically. The hydrogen atoms were calculated to their idealised positions as riding atoms with isotropic thermal parameters

[7] CrysAlisPro 1.171.42.80a, **2023**, Rigaku Oxford Diffraction.
[8] Sheldrick, G.M. (**2015**). *Acta Cryst.* A71, 3-8.
[9] Dolomanov, O.V., Bourhis, L.J., Gildea, R.J, Howard, J.A.K. & Puschmann, H. (**2009**), *J. Appl. Cryst.* 42, 339-341.
[10] Sheldrick, G.M. (**2015**). *Acta Cryst.* C71, 3-8.

as 1.2 x C for CH_2, CH and 1.5 x C for CH_3 and 1.5 x O for O atom. The structures were drawn with Mercury.[11] Crystallographic data was deposited with the accession number **2310461** and can be obtained free of charge from the Cambridge Crystallographic Data Centre via www.ccdc.cam.ac.uk/structures.

**Compound name**	**10**
CCDC deposition number	2310461
Empirical formula	$C_{13}H_{21}NO_2$
Formula weight	223.31
Temperature (K)	120.00(10)
Crystal system, space group	Orthorhombic, Pbca
a, b, c (Å)	11.7976(3), 9.0605(2), 22.6373(5)
α, β, γ (°)	90, 90, 90
Volume (Å^3)	2419.75(10)
Z	8
ρ_{calc} (g/cm^3)	1.226
μ (mm^{-1})	0.649
F(000)	976.0
Crystal size (mm^3)	0.275 x 0.19 x 0.129
**Data collection**	
Diffractometer	Rigaku XtaLAB Synergy R
Radiation	CuKα (λ = 1.54184 Å)
	Gaussian, CrysAlisPro 1.171.42.80a (Rigaku Oxford Diffraction, 2023)
Absorption correction	Empirical absorption correction using spherical harmonics, implemented in SCALE3 ABSPACK scaling algorithm.
T_{min}, T_{max}	0.581, 1.000
2Θ range for data collection (°)	7.81 to 133.498

[11] Mercury 2022.3.0 (Build 364735). C. F. Macrae, I. Sovago, S. J. Cottrell, P. T. A. Galek, P. McCabe, E. Pidcock, M. Platings, G. P. Shields, J. S. Stevens, M. Towler and P. A. Wood, *J. Appl. Cryst.,* **2020**, *53*, 226-235, 2020.

Index ranges	$-13 \leq h \leq 14$, $-10 \leq k \leq 10$, $-25 \leq l \leq 26$
Reflections collected	14560
Independent reflections	2146 [R_{int} = 0.0538, R_{sigma} = 0.0360]
**Refinement**	
Data/restraints/parameters	2146/0/149
Goodness-of-fit on F^2	1.093
Final R indexes [I>=2σ (I)]	R_1 = 0.0806, wR_2 = 0.2372
Final R indexes [all data]	R_1 = 0.0851, wR_2 = 0.2410
Largest diff. peak/hole (eÅ^{-3})	0.62/-0.33

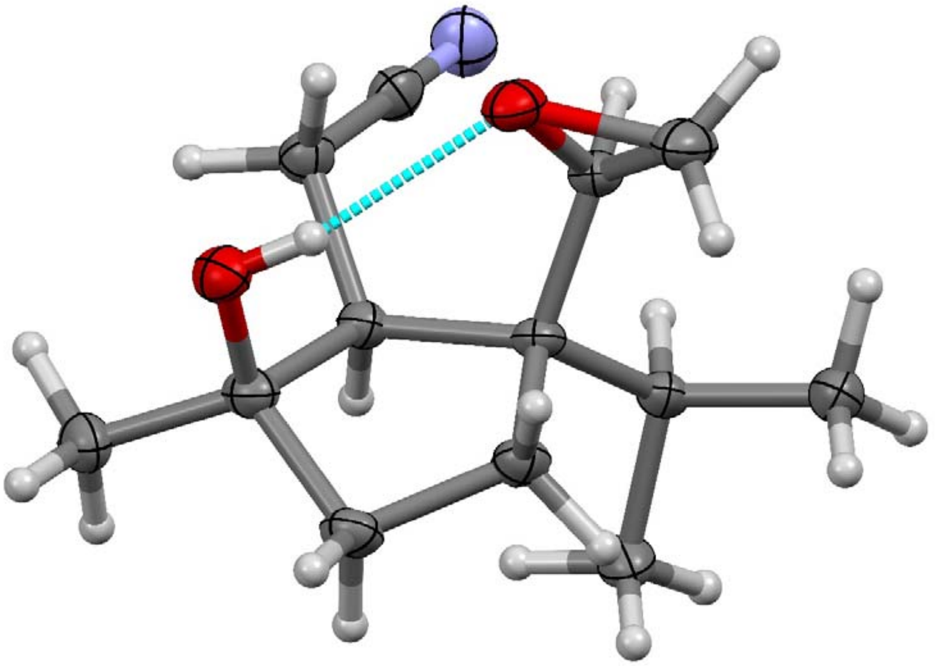

Figure S1. ORTEP plot of 10. The thermal displacement parameters are shown at 50% probability level.

2.4 Comparison of ^{1}H spin-spin coupling constants in (±)-4a and (±)-4b

The ^{1}H NMR spin-spin coupling constants of the synthesized bicyclo[3.2.0]heptane intermediates (±)-**4a** and (±)-**4b** were obtained from the ^{1}H NMR spectra of (±)-**4a** and (±)-**4b**,

and compared to the reported values for humilisin E (**1a**).[12] The vicinal coupling constants corresponding to H11-H10 ($^3J_{H11\text{-}H10}$) for (±)-**4a** and (±)-**4b** were found to be 5.8 and 5.4 Hz, respectively, while the reported H11-H10 coupling constant ($^3J_{H11\text{-}H10}$) for humilisin E was 6.5 Hz. Similarly, the vicinal H10-H2 coupling constants ($^3J_{H10\text{-}H2}$) for (±)-**4a** and (±)-**4b** were 8.3 Hz and 7.8 Hz respectively, compared to the reported value of 9.0 Hz for humilisin E (**1a**). Overall, the coupling constants are in agreement with the assigned stereochemistry for (±)-**4a** and (±)-**4b**.

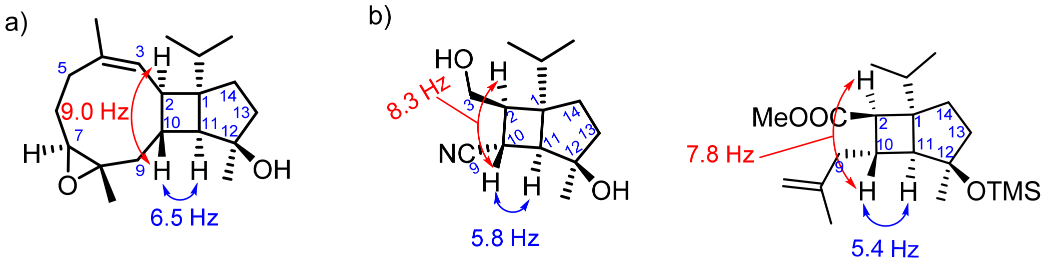

Humilisin E **(1a)** (±)-**4a** (±)-**4b**

Figure S2. Comparison of $^3J_{HH}$ spin-spin coupling constants observed in the ^{1}H NMR spectra of the cyclobutane ring of a) humilisin E and b) synthesized bicyclo[3.2.0]heptane cores of humilisin E, (±)-**4a** and (±)-**4b.**

2.5 1-D NOE experiment

2.5.1 NOE interactions in (±)-4a

The relative stereochemistry of (±)-**4a** was further confirmed based on 1H 1D NOE NMR (DPGFSE-NOE) experiments. Selectively irradiating the peak at δ = 3.70 ppm (H3) revealed interactions with the peaks at δ = 2.83, 1.91 and 1.70 ppm (H10, H13a and H14a) determining the relative stereochemistry of the cyclized product (±)-**4a**.

[12] Sun, L. L.; Li, W. S.; Li, J.; Zhang, H. Y.; Yao, L. G.; Luo, H.; Guo, Y. W.; Li, X. W. *J. Org. Chem.* **2021**, *86* (4), 3367–3376.

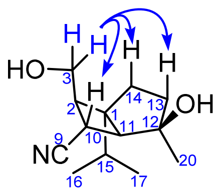

(±)-**4a**

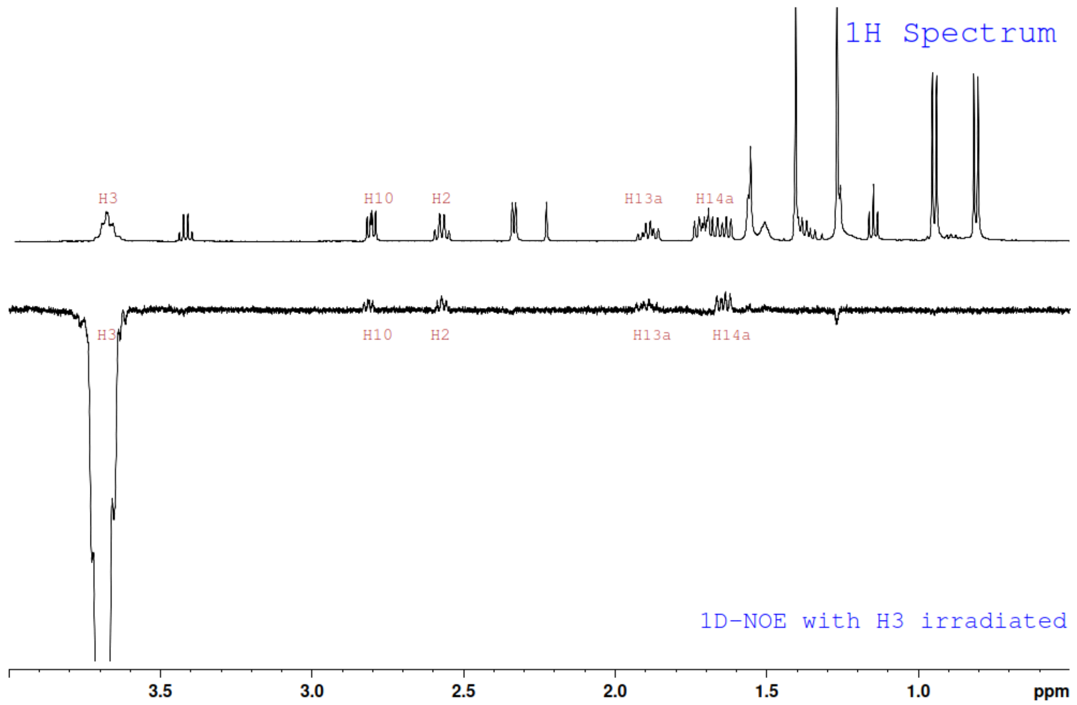

2.5.2 NOE interactions in (±)-**4b**

The relative stereochemistry was determined by selective 1D NOE (DPGFSE-NOE) experiments. Selectively irradiating the peak at δ = 1.74 ppm assigned as H11 revealed interactions with H2 (2.65 ppm), H9a/b (2.31, 2.06 ppm), H15 (1.56 ppm) and H20 (1.15 ppm). Irradiation at δ = 2.65 ppm assigned as H2 shows prominent interactions with H9a/b (2.31, 2.06 ppm), H11 (1.73 ppm), H15 (1.56 ppm). These interactions aided in concluding the relative stereochemistry of the cyclized product (±)-**4b** which was found to be desirable as reported in the natural product.

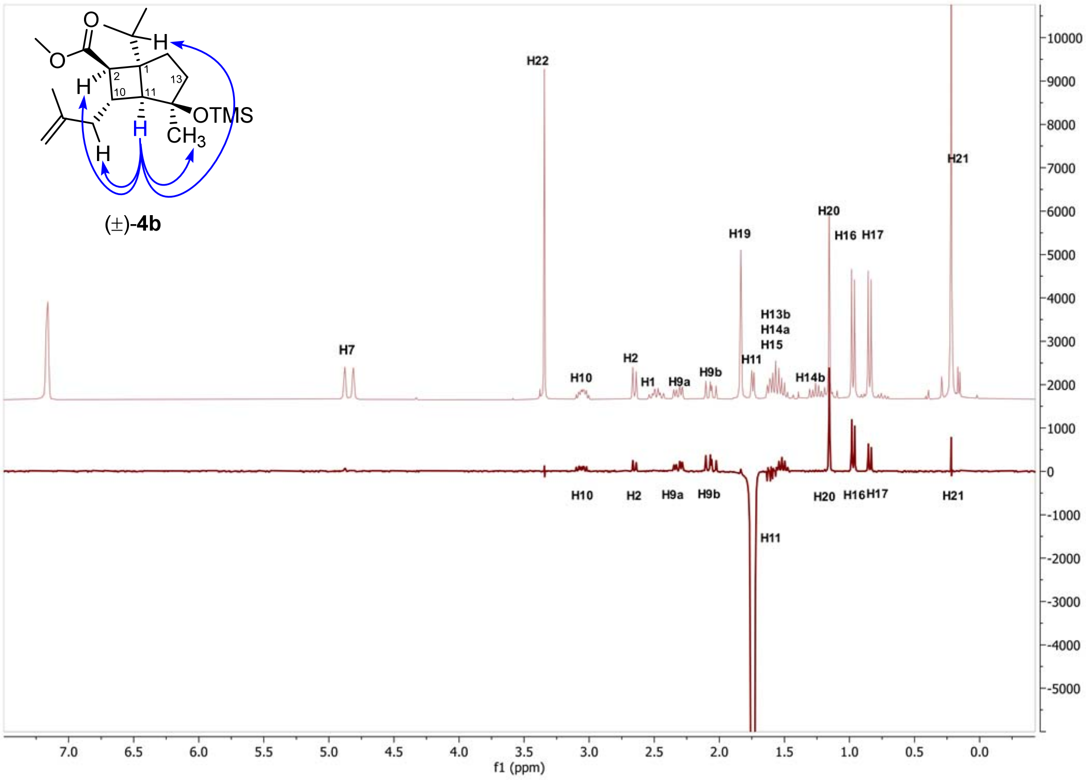

(±)-4b
H22
H7
H10
H2
H1
H9a
H9b
H19
H11
H13b
H14a
H15
H14b
H20
H16 H17
H21
f1 (ppm)

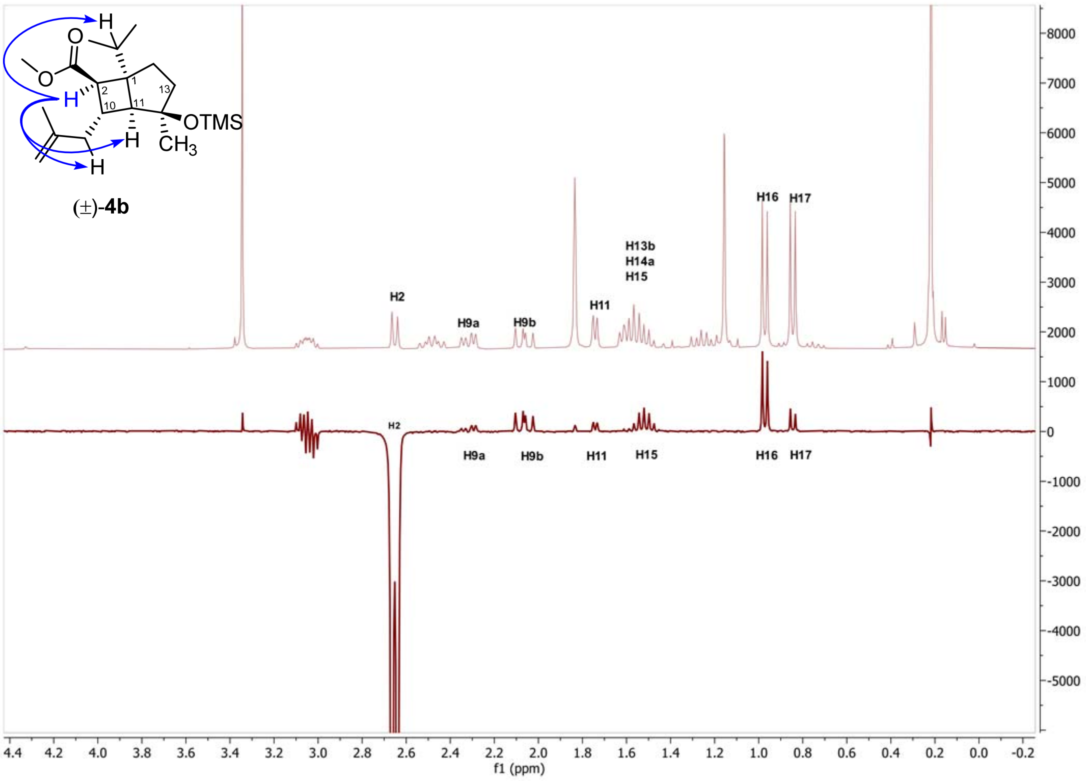

(±)-4b
H2
H9a
H9b
H11
H13b
H14a
H15
H16
H17
f1 (ppm)

3 Computational details

3.1 Computational procedures

The DFT-minimized structures of **1a**, **2a** and (±)-**11** were obtained as follows:

1. **Conformational analysis** of the structures was first explored via Monte Carlo sampling using the OPLS4 force field. The conformational analysis was carried out using the *MacroModel* software embedded within the Schrödinger Maestro suite of software.[13]
2. -**Geometry optimizations**. The most favored conformers obtained in the Monte Carlo conformational search were subject to DFT calculations. The geometry optimizations were carried out at the ωB97X-D3/6-311G(d,p) level of theory.[14]
3. **Single-point calculations and solvent effects**. For the most stable conformers, single-point calculations (DFT) were carried out at the ωB97X-D3/CC-PVDZ[15] level of theory. The solvent effects were calculated using the SM8 continuum model,[16] corresponding to a temperature of 298 K and a standard-state concentration of 1 M in $CHCl_3$. Steps 2 and 3 were carried out using the Jaguar software embedded within the Schrödinger Maestro.[17]

   Alternative DFT functionals, such as the M06/2X functional and other solvation models (PBF) were also briefly explored, but these changes had only minimal effects on the relative energies of the conformers/rotamers. Only the ωB97X-D3/CC-PVDZ / SM8 gas phase energies and solution energies are reported herein.

---

[13] Schrödinger Release 2023-3, Macromodel and Jaguar, Schrödinger, LLC, New York, NY, 2023.
[14] J.-D. Chai and M. Head-Gordon, "Long-range corrected hybrid density functionals with damped atom-atom dispersion corrections," *Phys. Chem. Chem. Phys.*, **10** (2008) 6615-20.
[15] Dunning, T. H., Jr., Gaussian basis sets for use in correlated molecular calculations. I. The atoms boron through neon and hydrogen. *J. Chem. Phys.* **1989**, *90*, 1007.
[16] Marenich, A. V.; Olson, R. M.; Kelly, C. P.; Cramer, C. J.; Truhlar, D. G. Self-consistent reaction field model for aqueous and nonaqueous solutions based on accurate polarized partial charges, *J. Chem. Theory Comput.* **2007**, *3*, 2011.
[17] Bochevarov, A.D.; Harder, E.; Hughes, T.F.; Greenwood, J.R.; Braden, D.A.; Philipp, D.M.; Rinaldo, D.; Halls, M.D.; Zhang, J.; Friesner, R.A., "Jaguar: A high-performance quantum chemistry software program with strengths in life and materials sciences," *Int. J. Quantum Chem.*, **2013**, 113, 2110-2142. See also ref 4.

3.2 Computational Results

The most stable structures for compounds **1a, 2a** and (±)-**11** are depicted in Figure SS3.

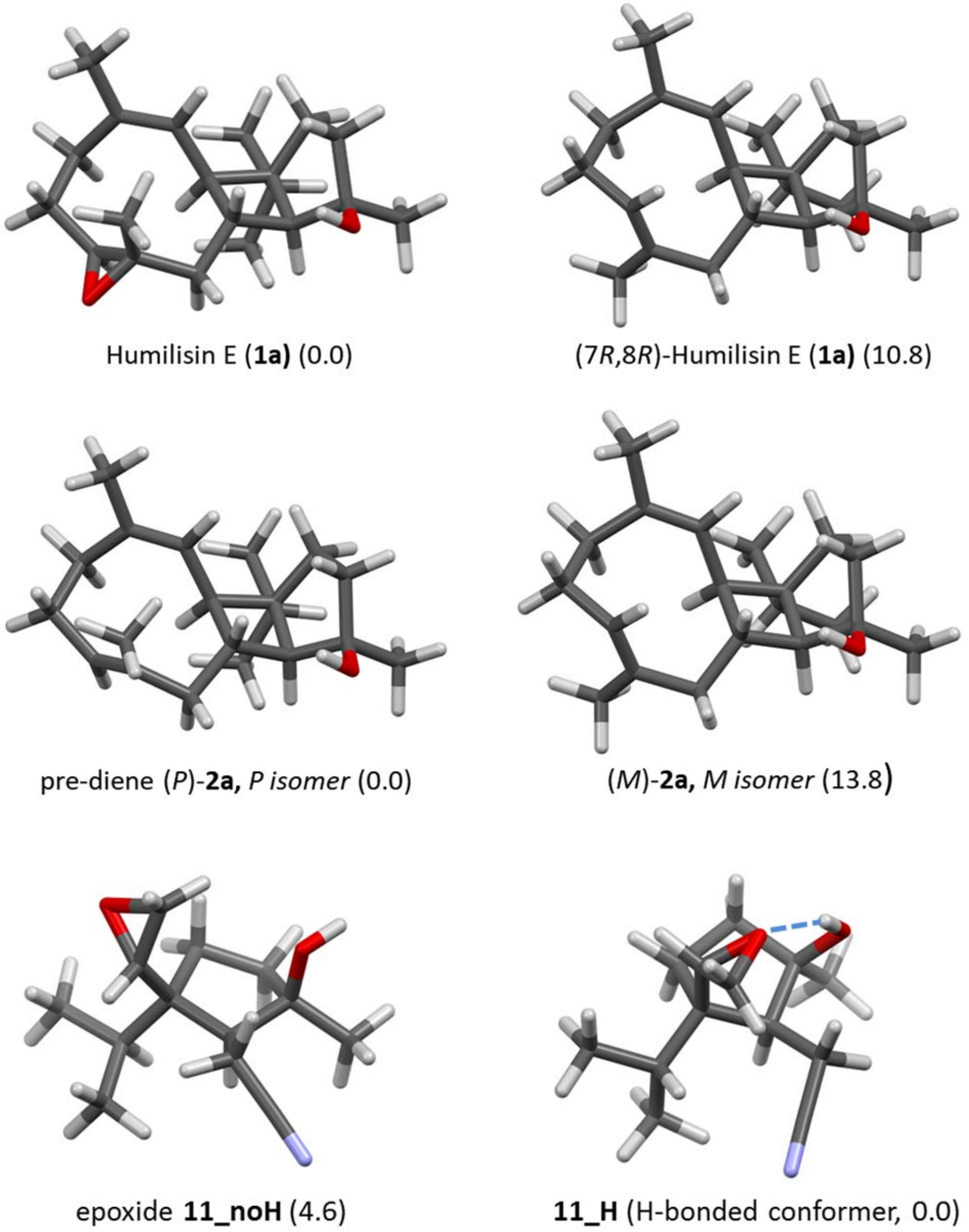

Figure S3. The most stable conformers of pre-diene **2a** (*P* and *M* isomers), humilisin E (**1a**) and its (7*R*,8*R*)-diastereomer, and epoxide (±)-**11**. Relative stabilities are given in parenthesis (in kJ/mol). Internal hydrogen bonds are indicated by blue dotted lines.

3.3 Total energy data

The energy data computed for the wB97X-D3/CC-PVDZ optimized geometries of the structures discussed in the manuscript are listed in Table S1.

Table S1. Energy data (in a.u.) computed for ωB97X-D3/CC-PVDZ optimized structures.[a]

structures	E_0	E_0'	E_0sol'
**1a**	-931,822076	-931.615776	-931.633527
**(7*R*,8*R*)-1a**	-931.811994	--931.611717	-931.629396
**(*P*)-2a**	-856,600596	-856,415755	-856,431356
**(*M*)-2a**	-856,595482	-856,410462	-856,426086
**11_H**	-713.196826	-713.032339	--713.050096
**11_noH**	-713.195356	-713.029853	-713.048349

[a] Notation: E_o and E_o' refer to electronic energies computed at wB97X-D3/6-311G(d,p) and wB97X-D3/CC-PVDZ level of DFT, and E0sol' refer to the solution phase energies obtained with the SM8 solvation model. The relative stabilities discussed in the manuscript and in the SI are obtained from these final, solvation values.

3.4 Atomic coordinates of the computed structures

The atomic coordinates of the structures shown in Table S1 are provided in electronic format as xyz coordinate files.

4. Copies of NMR spectra and GC chromatograms

4.1 ^{1}H NMR Spectrum of S2

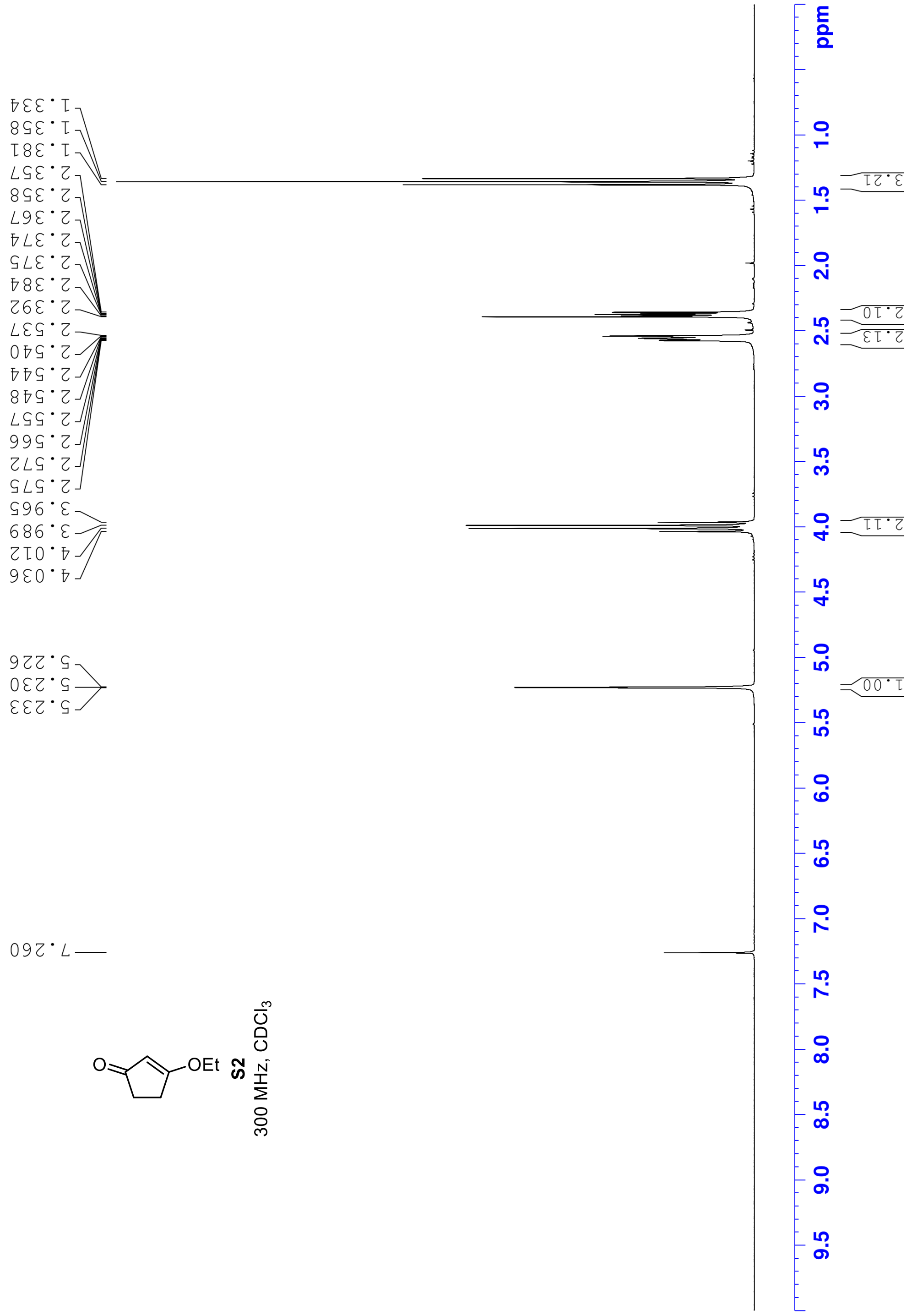

4.1 ^{1}H NMR Spectrum of S2 (Expansion)

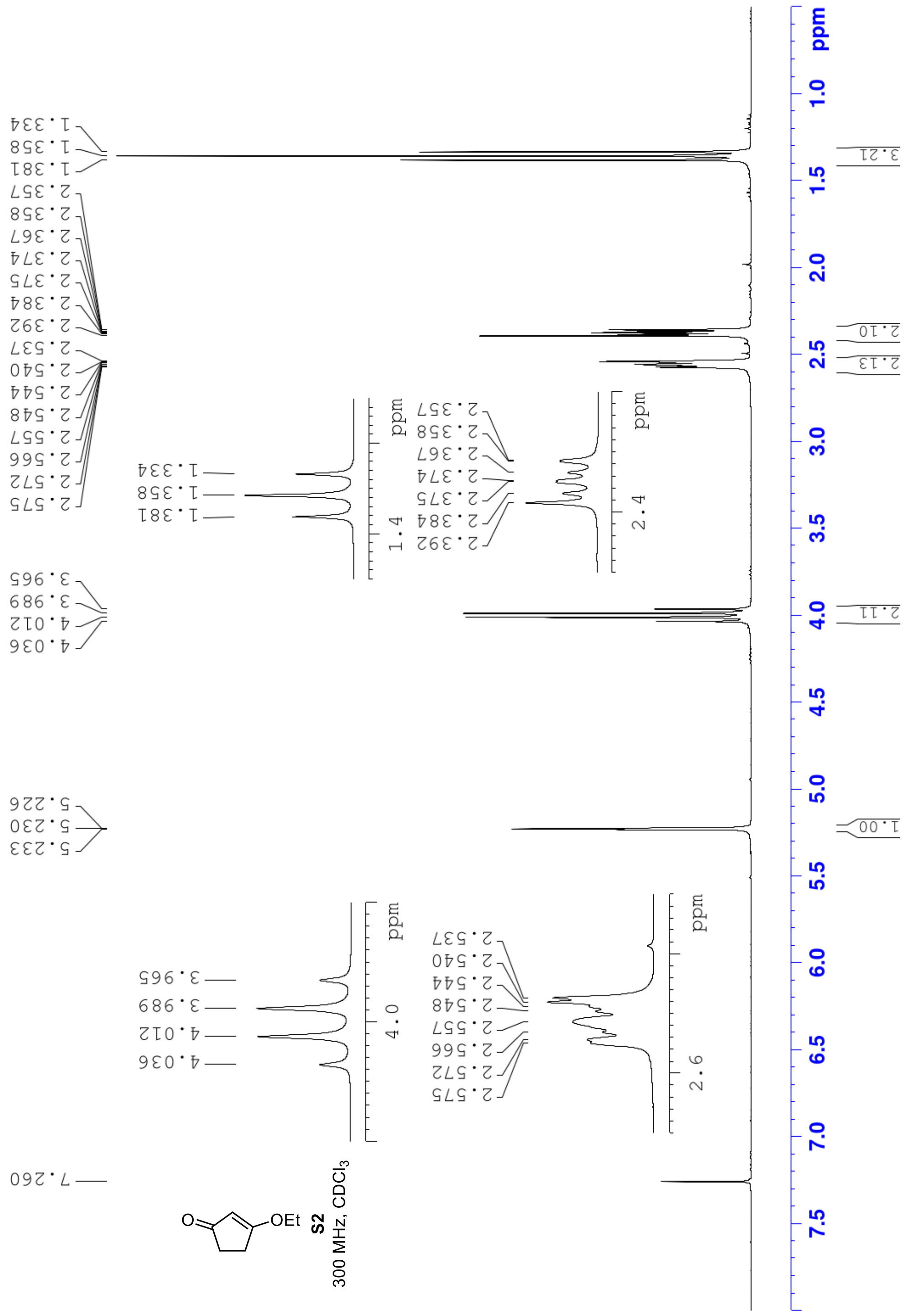

4.2 $^{13}C\{^1H\}$ NMR Spectrum of S2

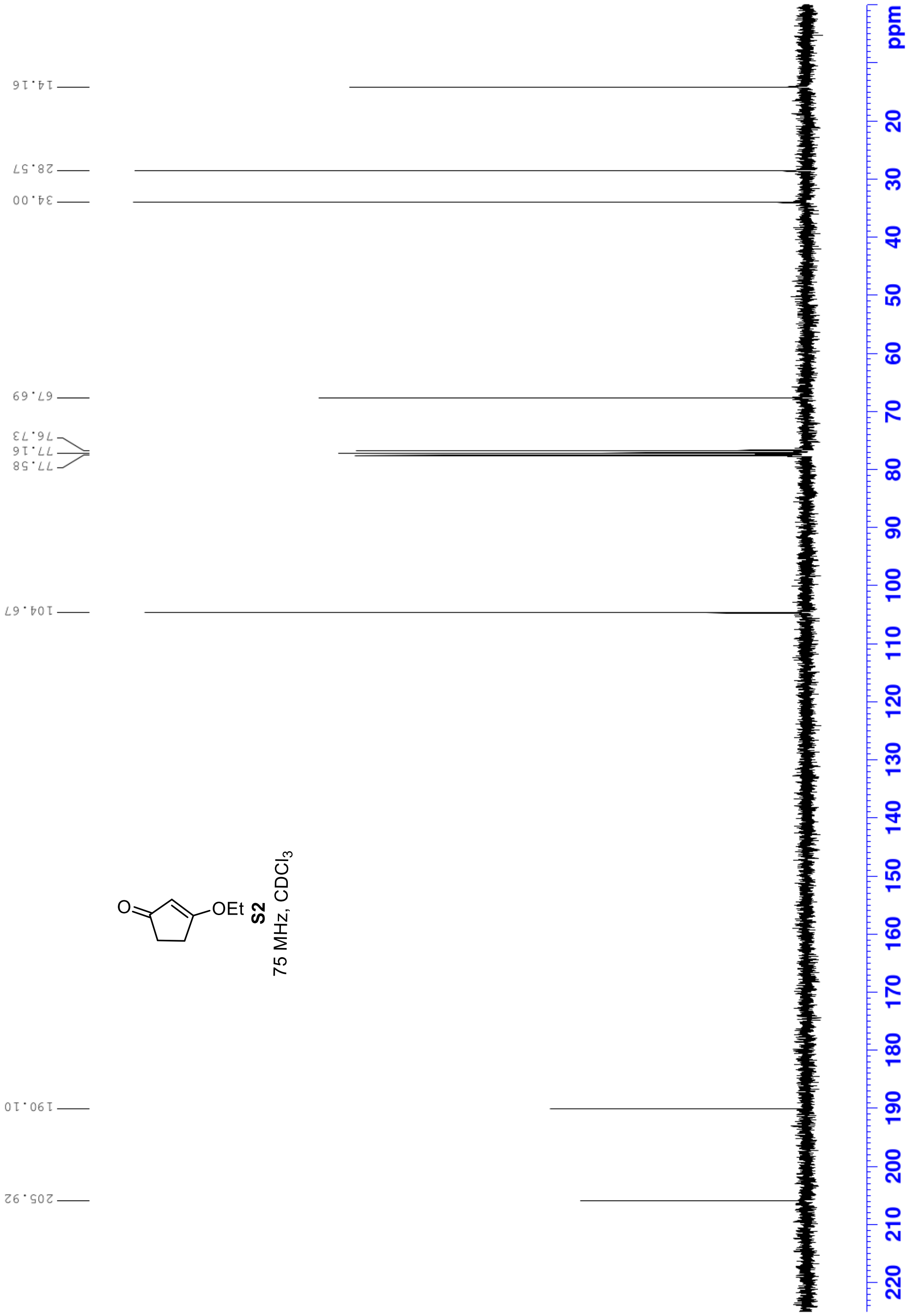

4.3 ^{1}H NMR Spectrum of 6

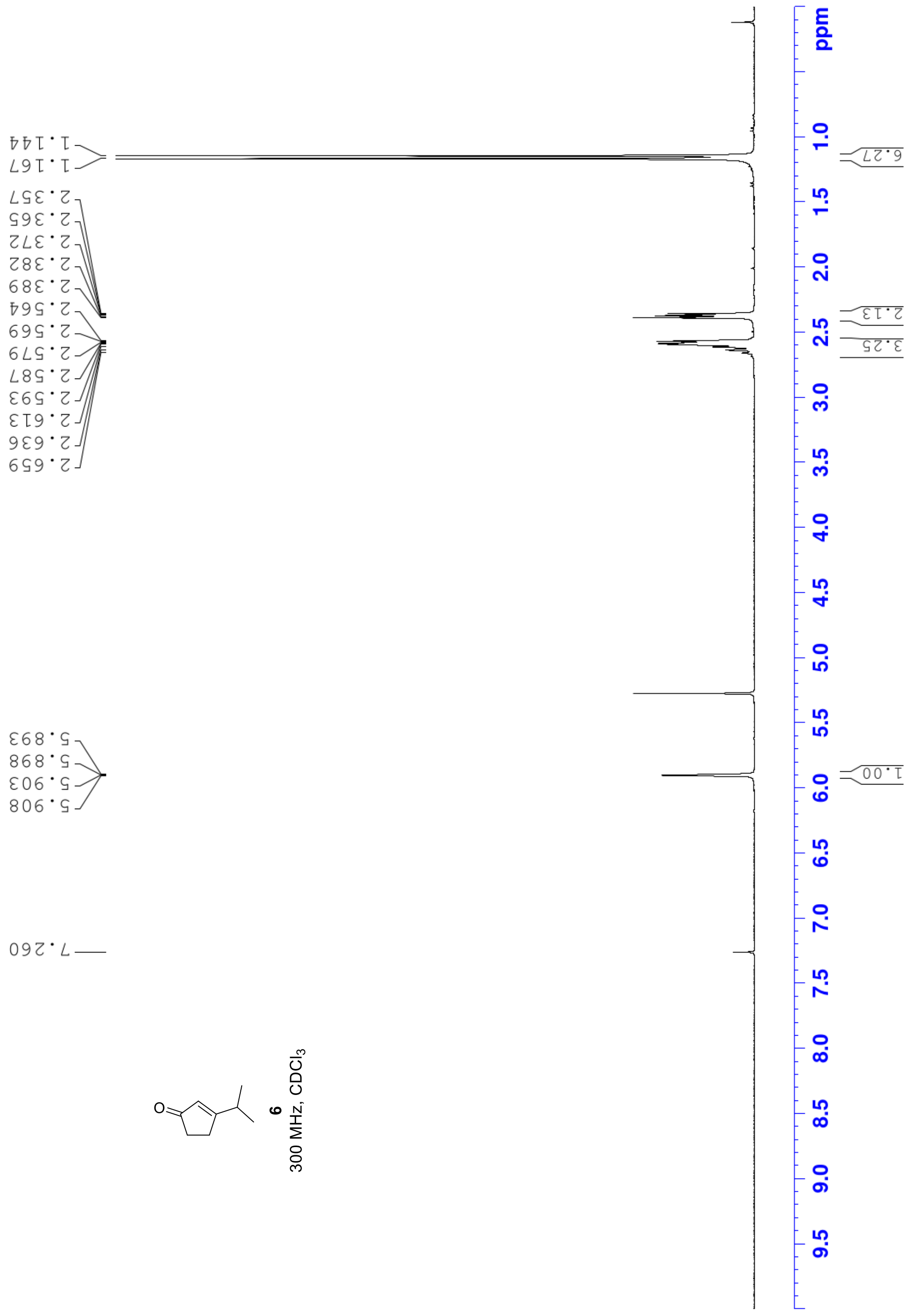

4.4 ¹H NMR Spectrum of 6 (Expansion)

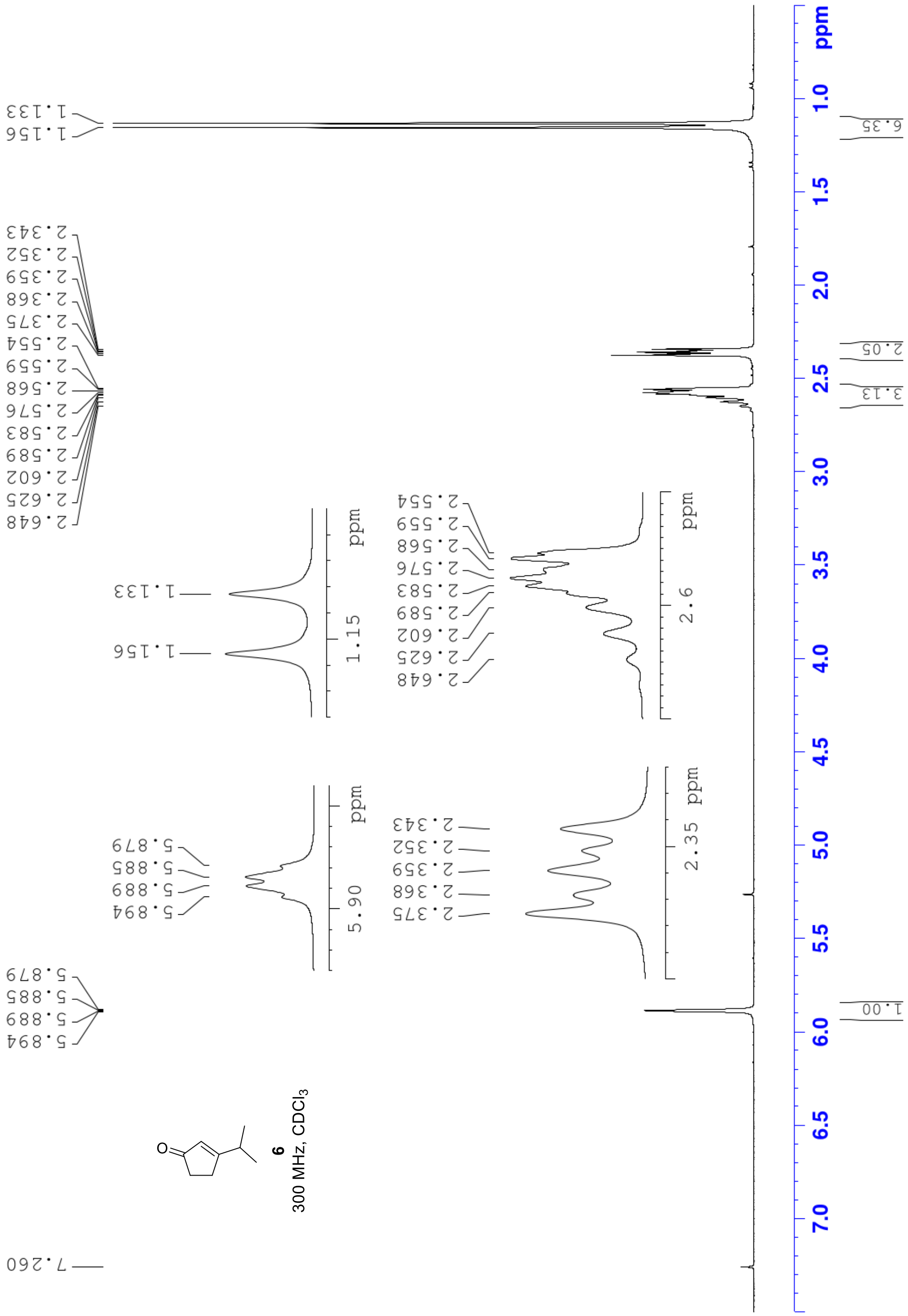

4.5 $^{13}C\{^1H\}$ NMR Spectrum of 6

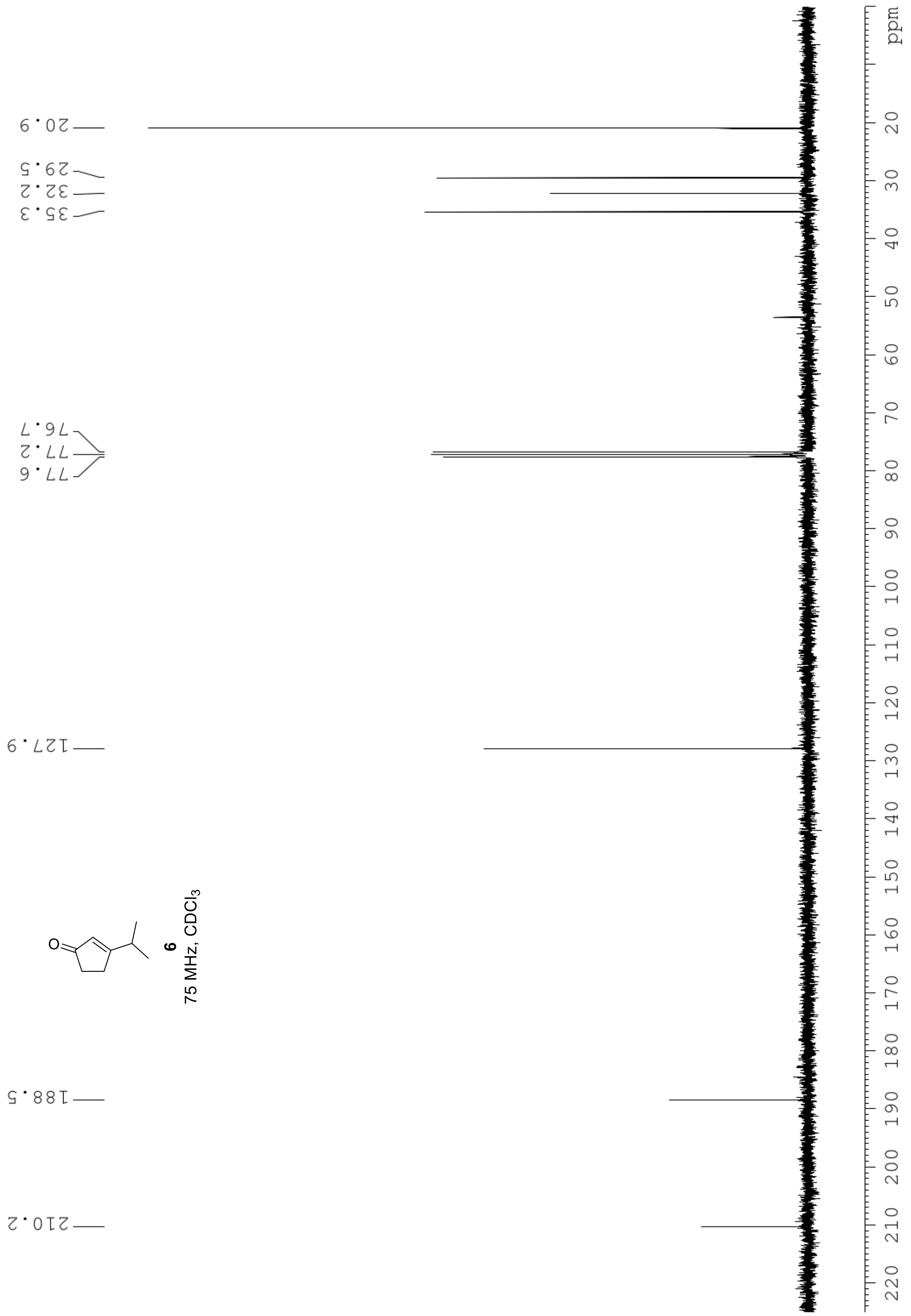

4.6 ^{1}H NMR Spectrum of (±)-7

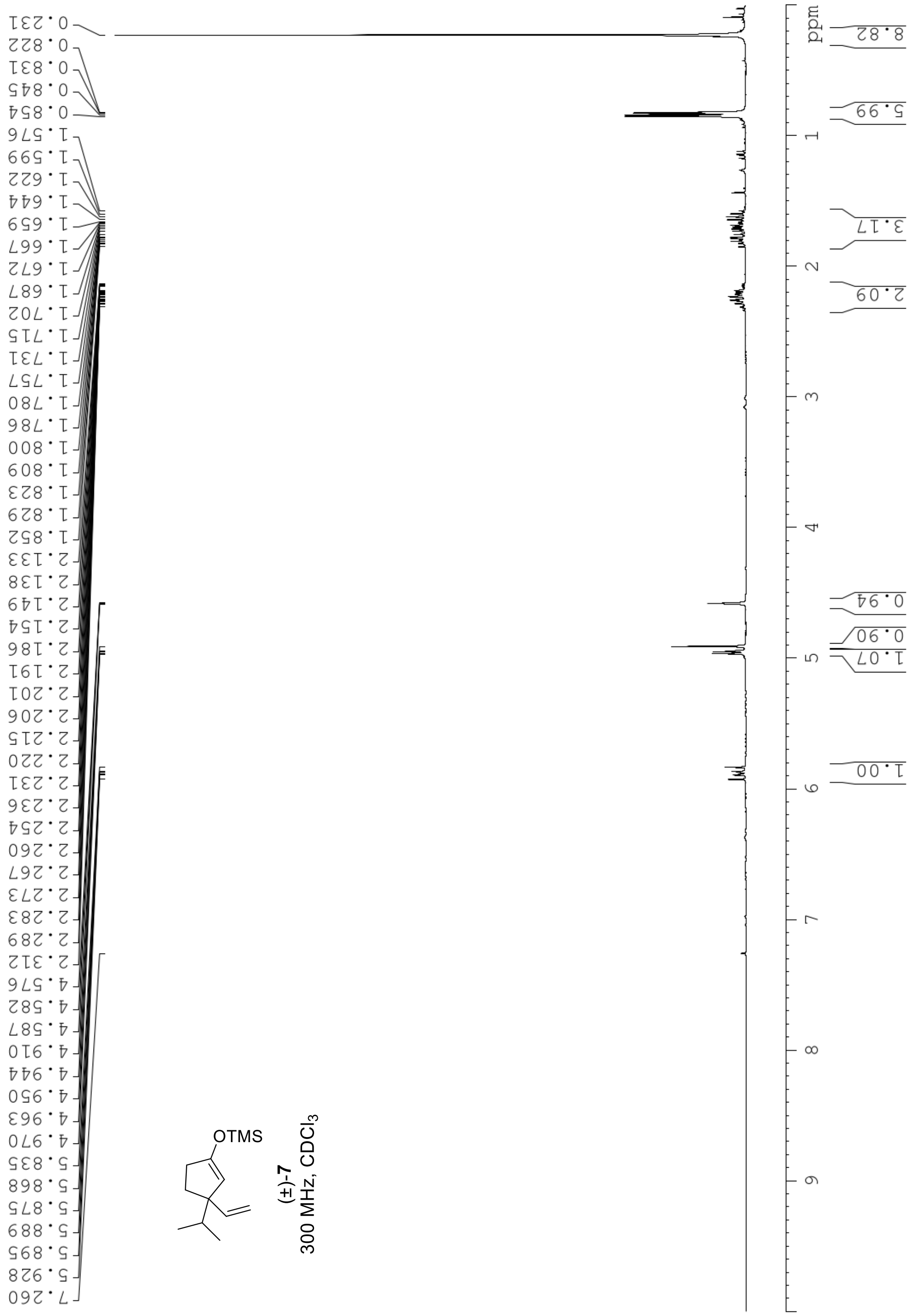

4.7 ^{1}H NMR Spectrum of (±)-7 (Expansion)

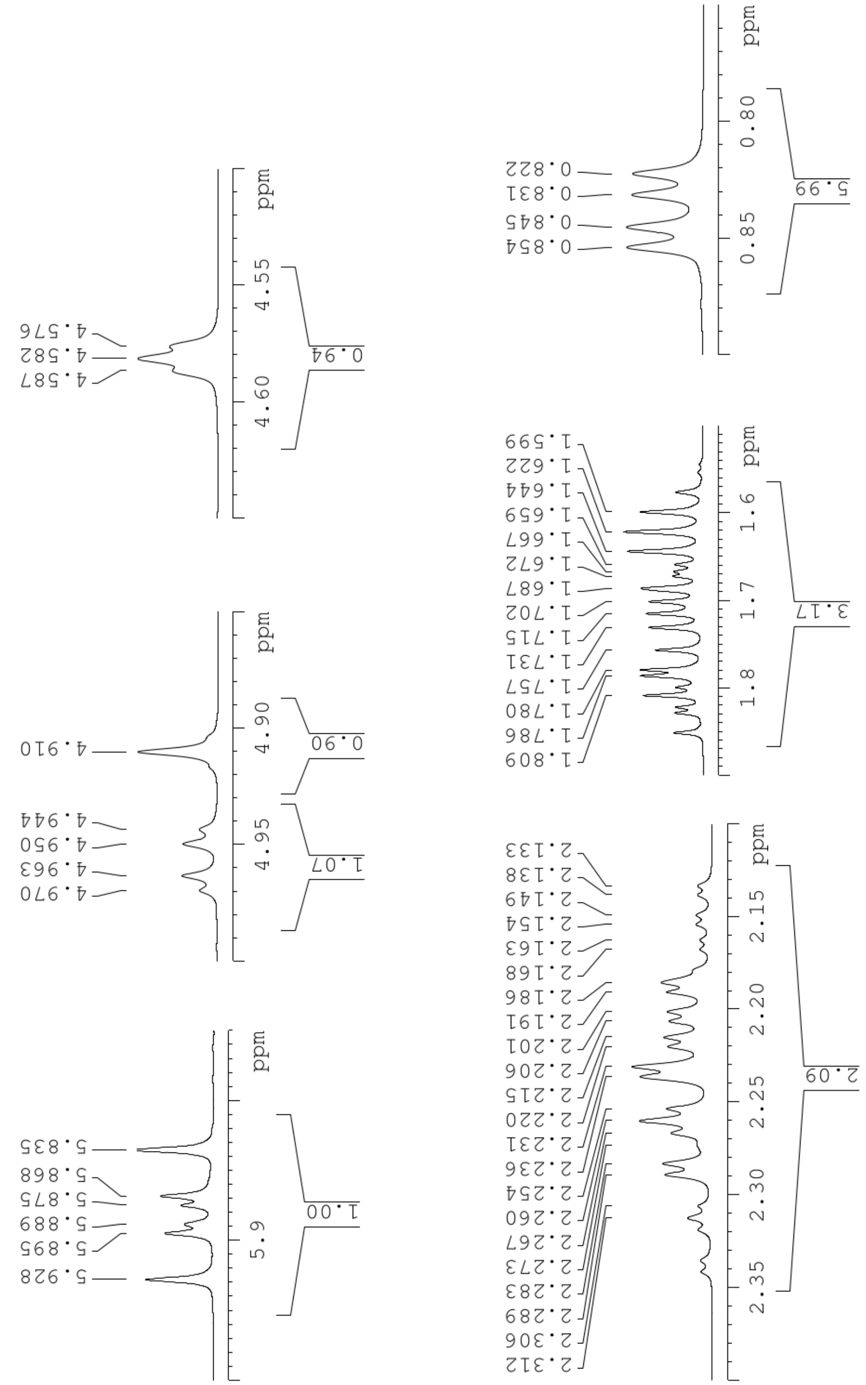

4.8 $^{13}C\{^{1}H\}$ NMR Spectrum of (±)-7

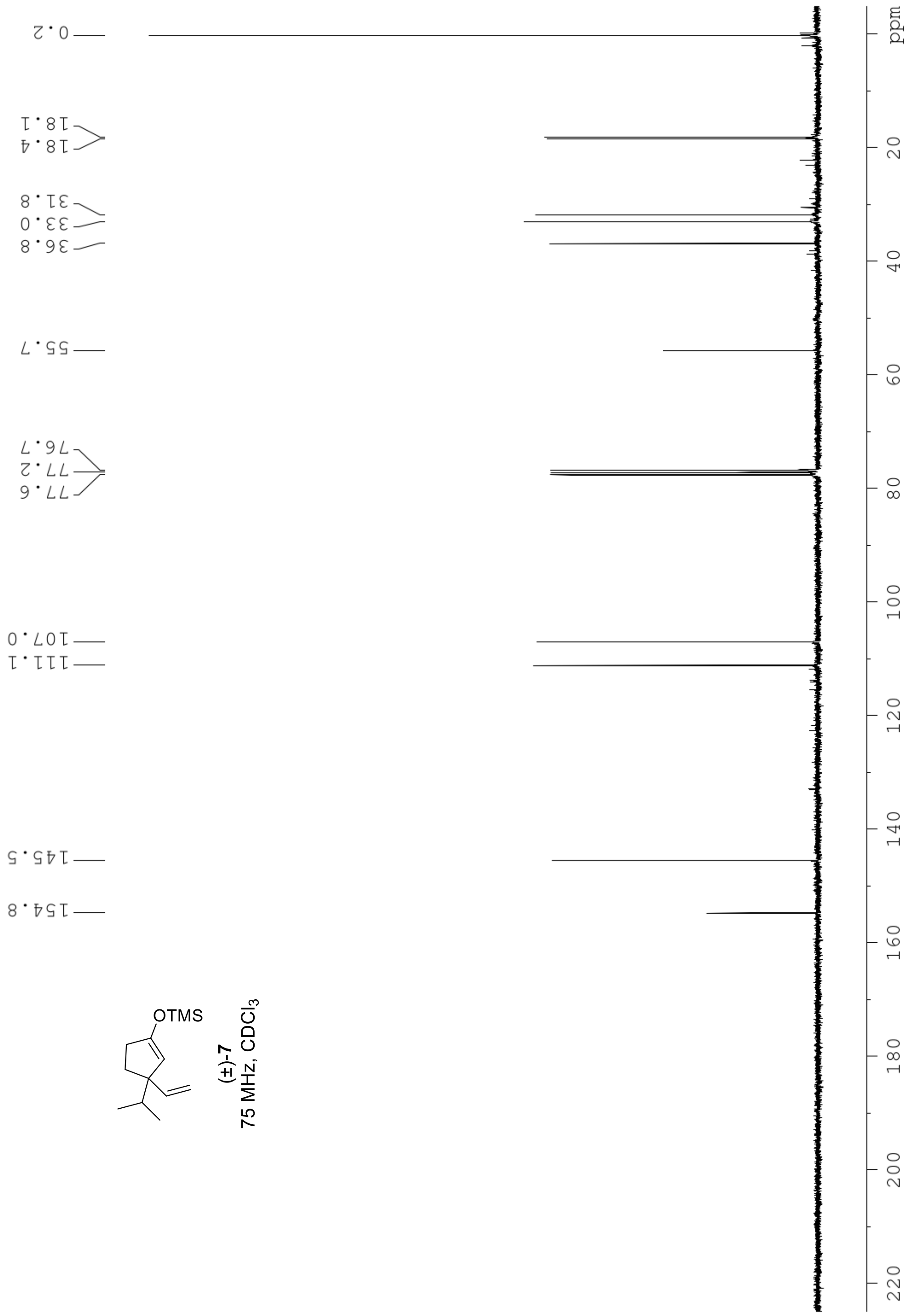

4.9 ^{1}H NMR Spectrum of (±)-8

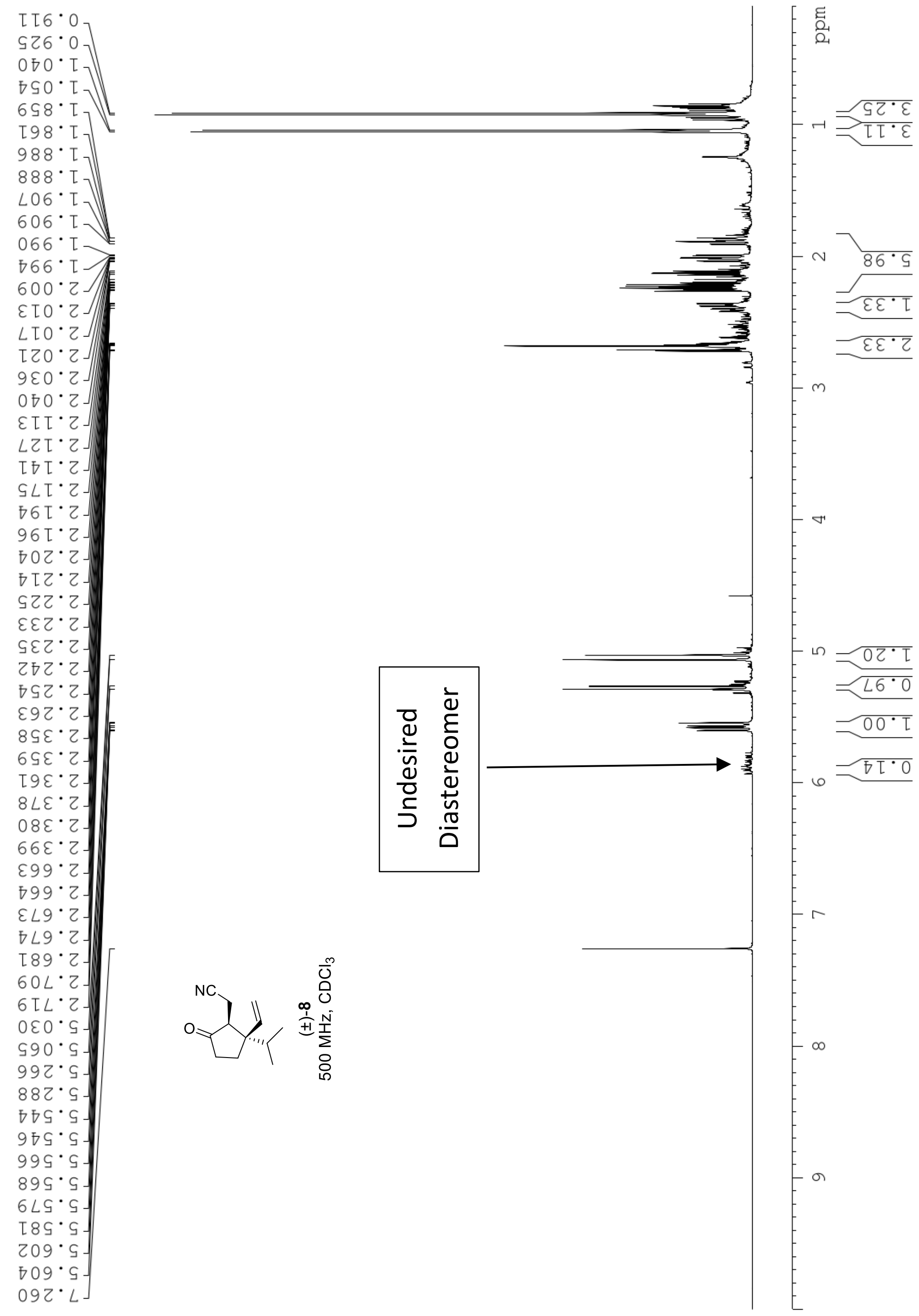

4.10 ^{1}H NMR Spectrum of (±)-8 (Expansion)

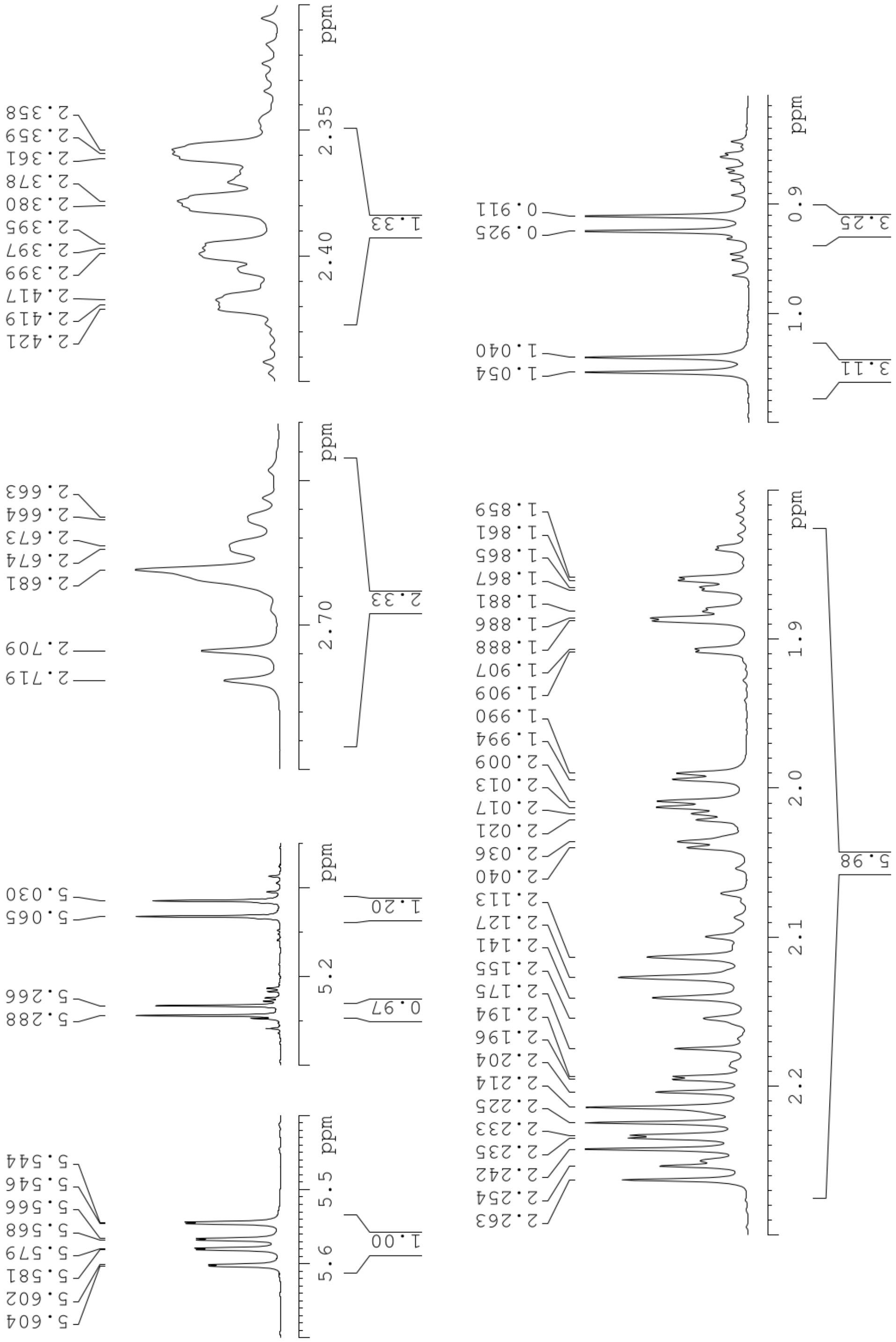

4.11 $^{13}C\{^1H\}$ NMR Spectrum of (±)-8

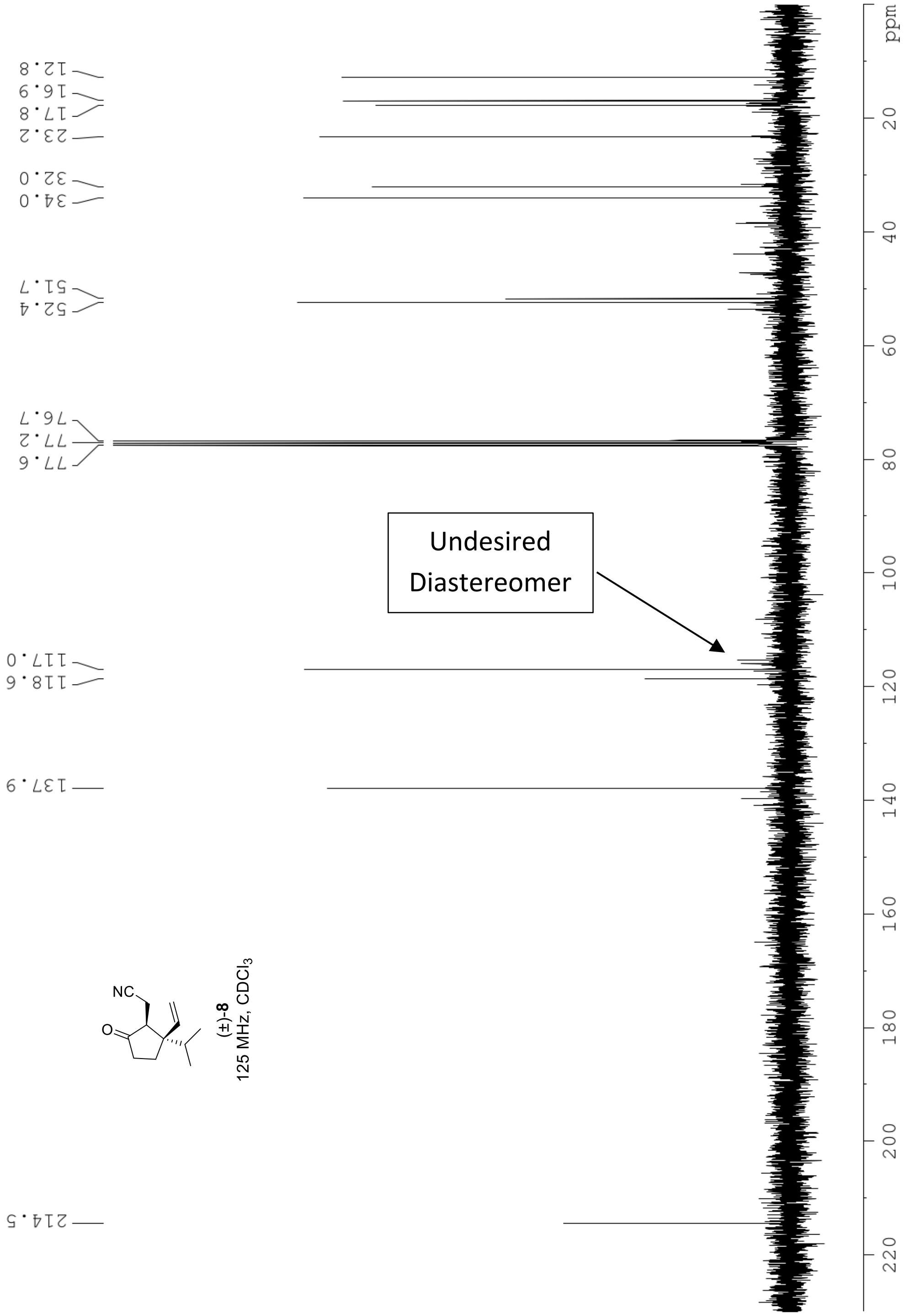

4.12 ^{1}H NMR Spectrum of (±)-9

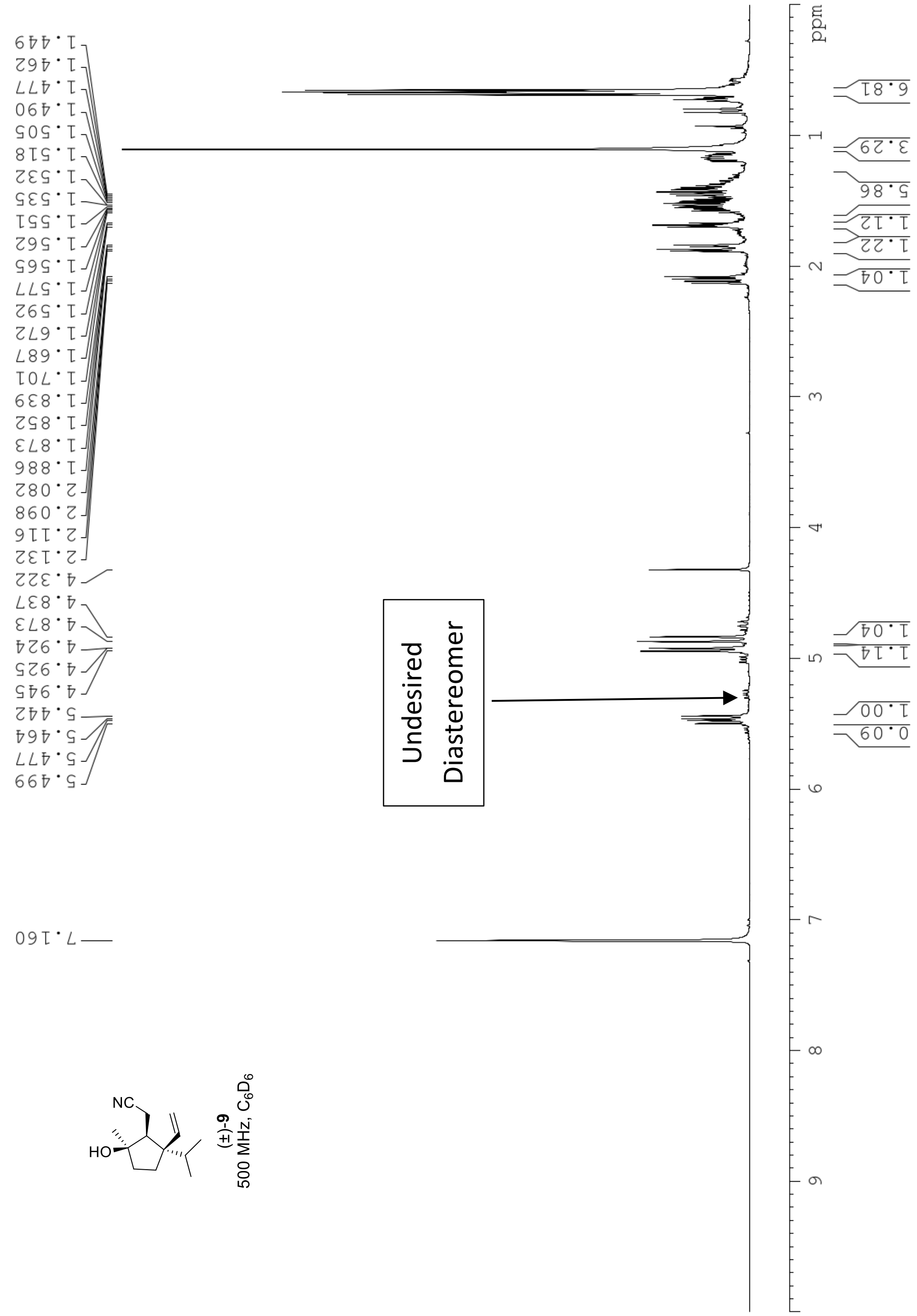

4.13 ^{1}H NMR Spectrum of (±)-9 (Expansion)

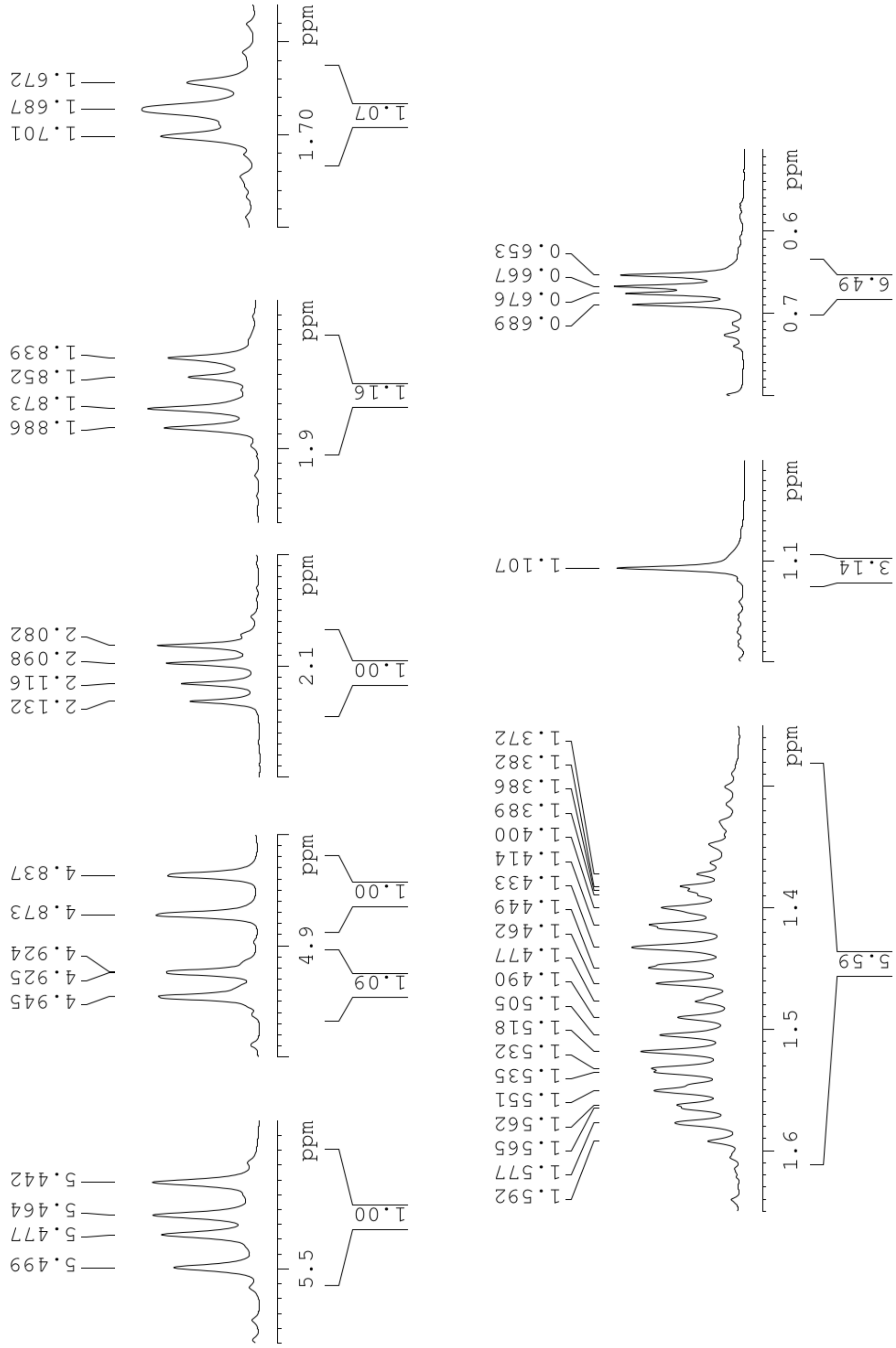

4.14 $^{13}C\{^{1}H\}$ NMR Spectrum of (±)-9

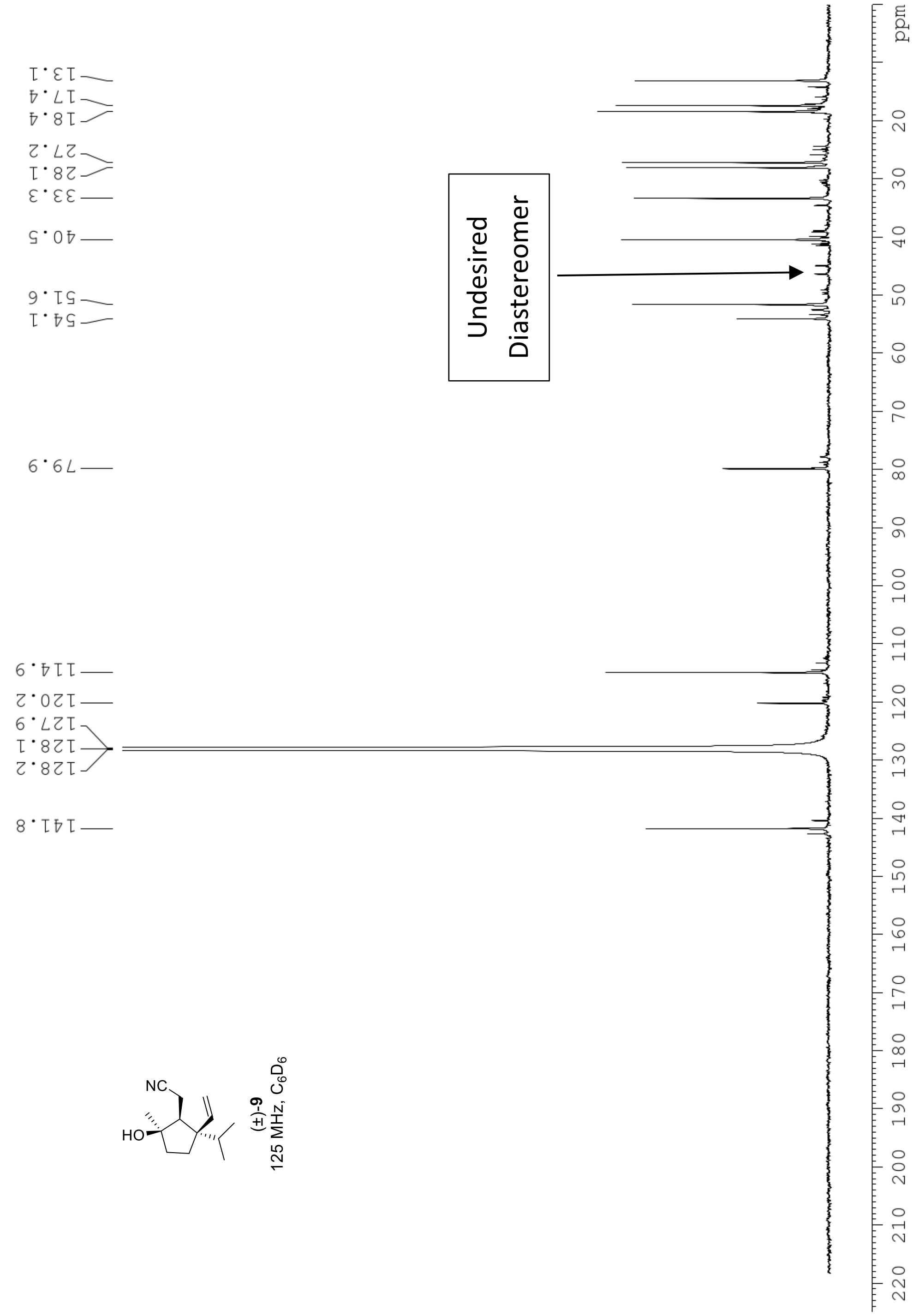

4.15 ¹H NMR Spectrum of (±)-10

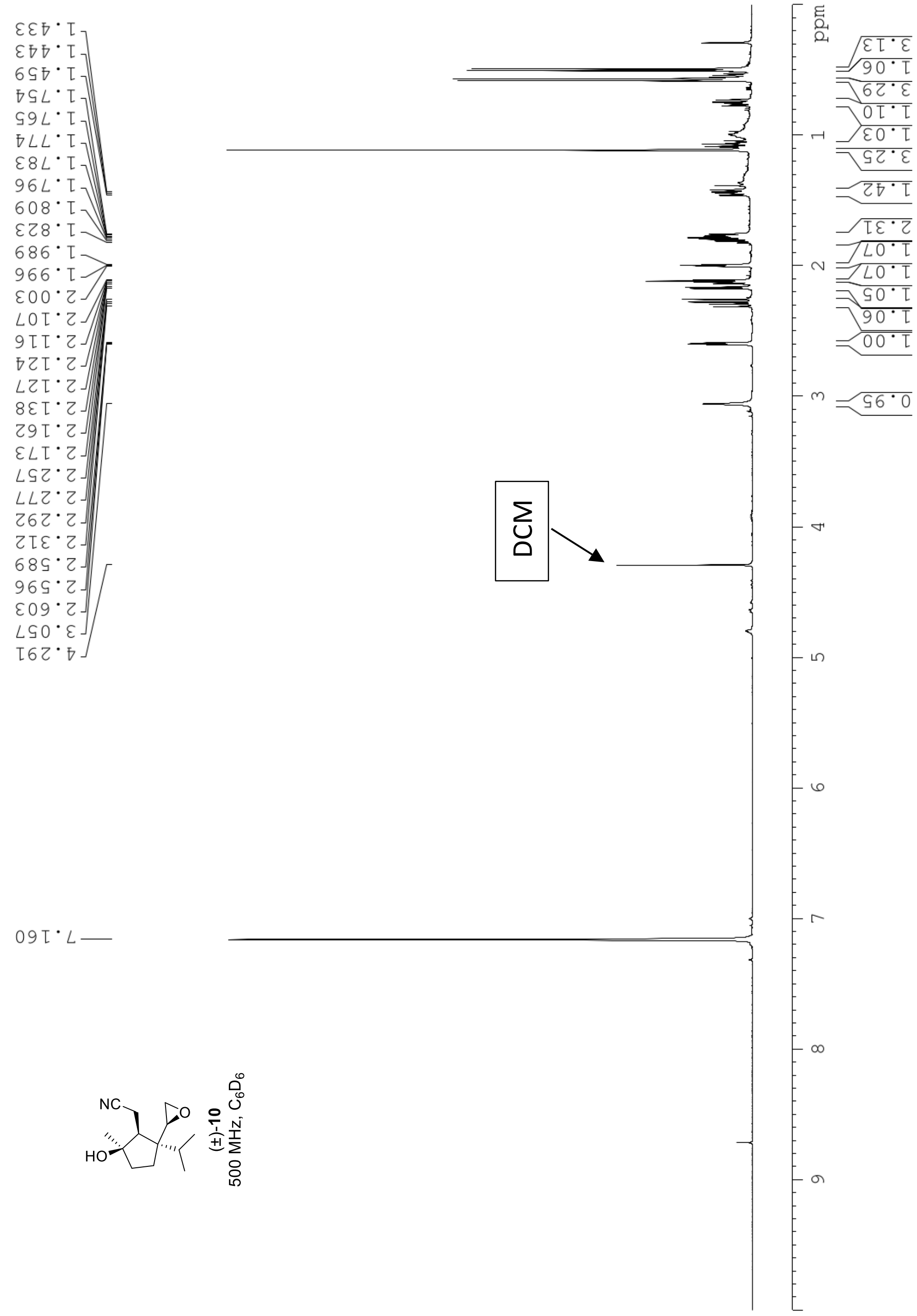

4.16 ^{1}H NMR Spectrum of (±)-10 (Expansion)

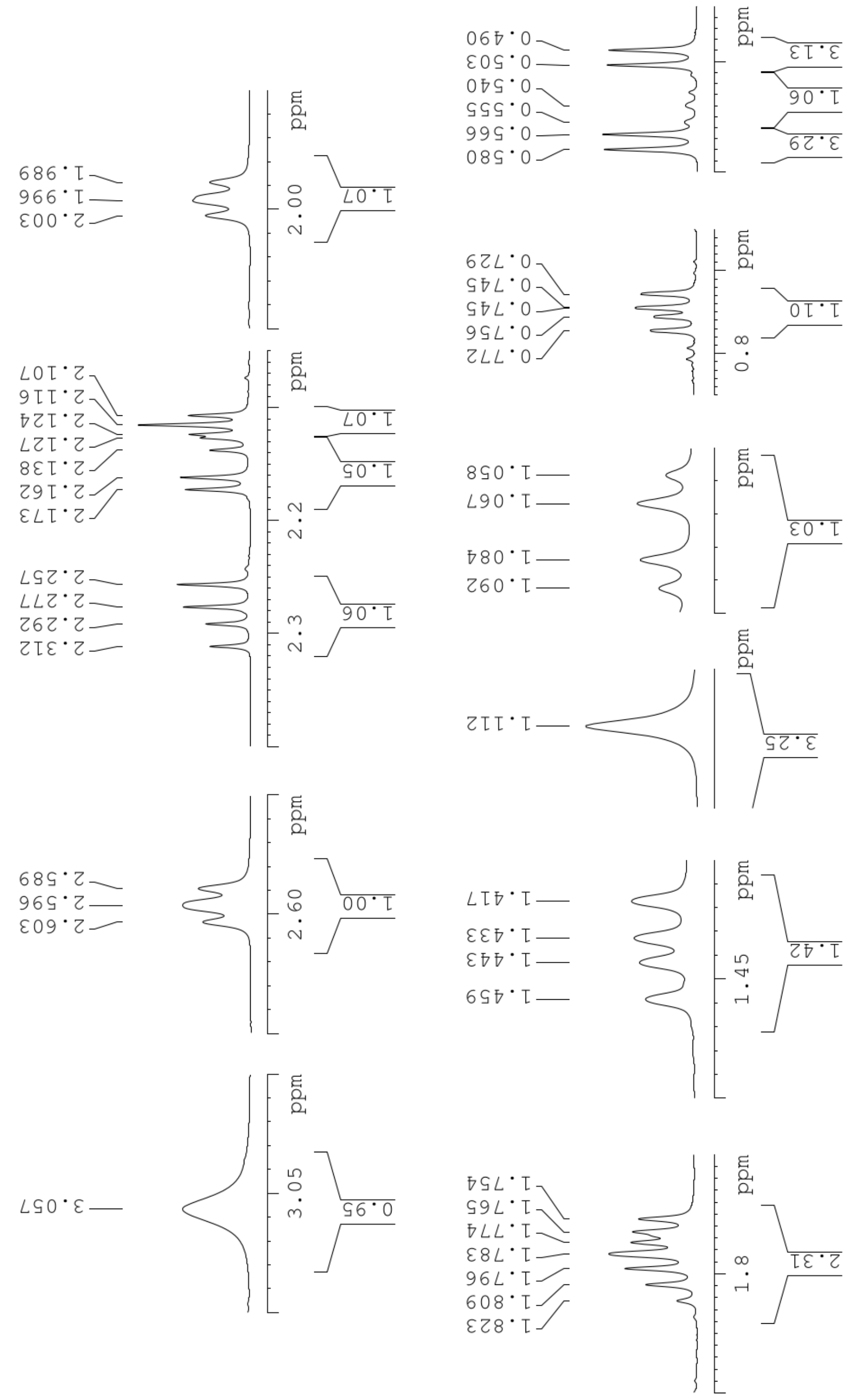

4.17 $^{13}C\{^1H\}$ NMR Spectrum of (±)-10

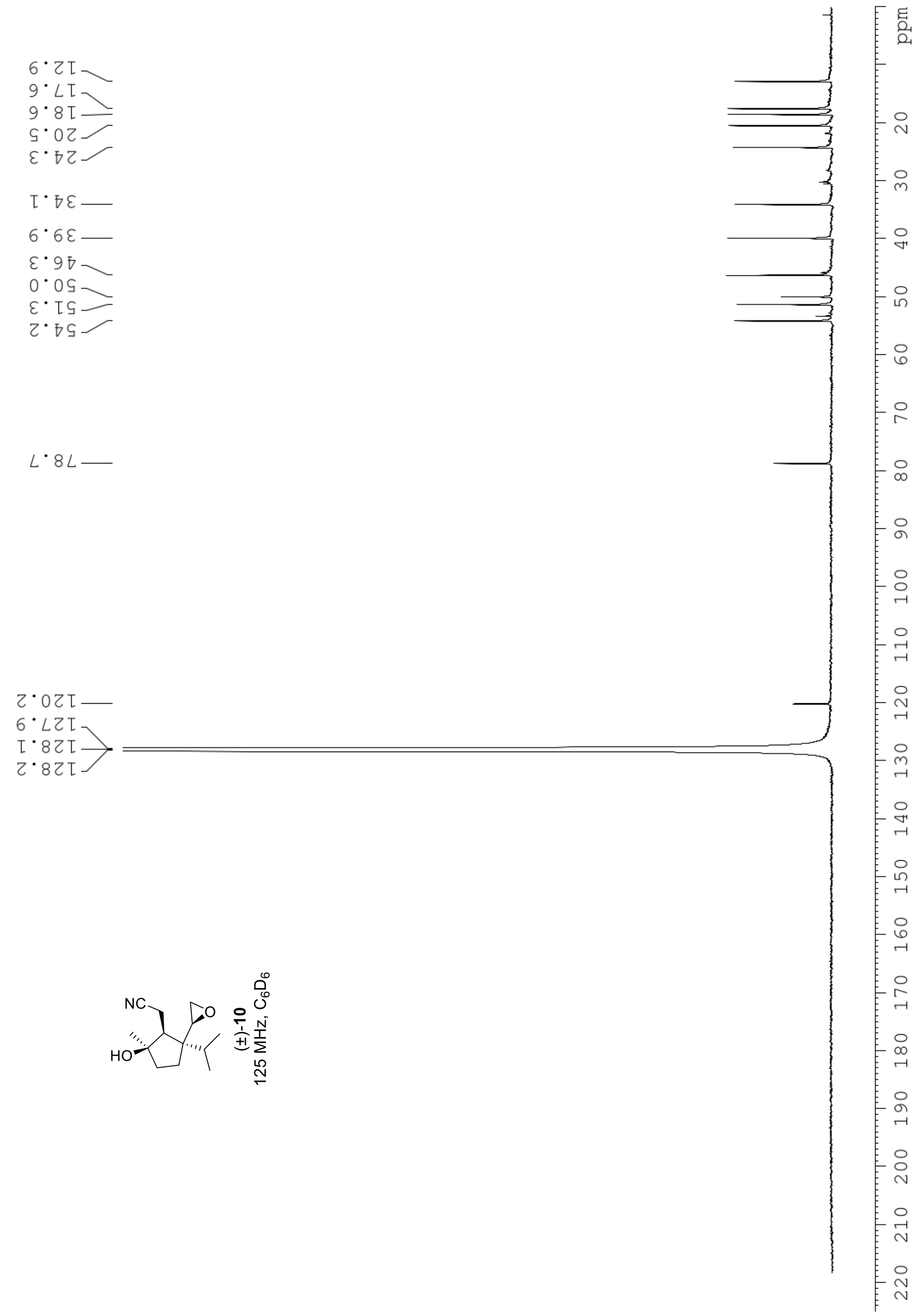

4.18 ^{1}H NMR Spectrum of (±)-11

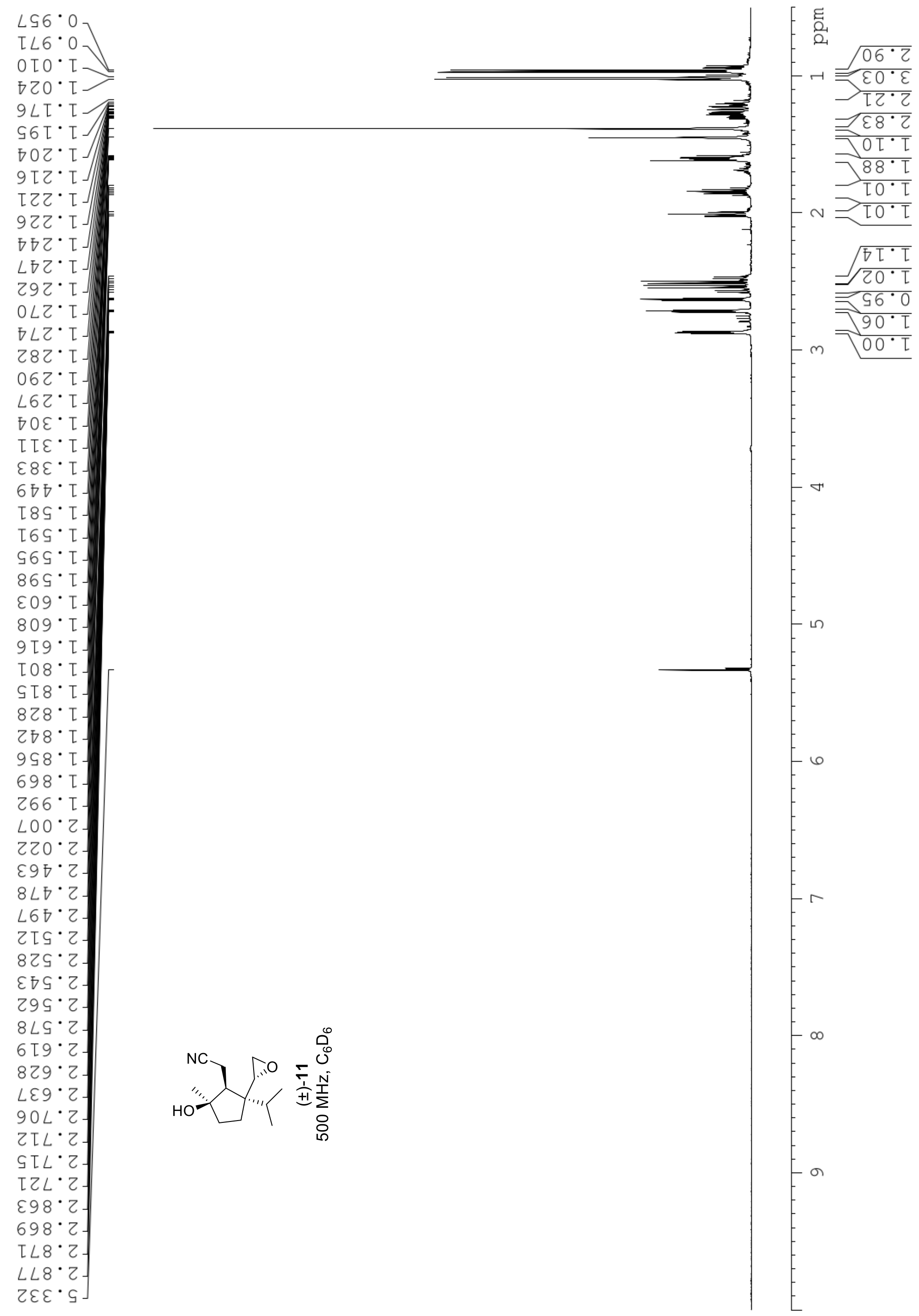

4.19 ^{1}H NMR Spectrum of (±)-11 (Expansion)

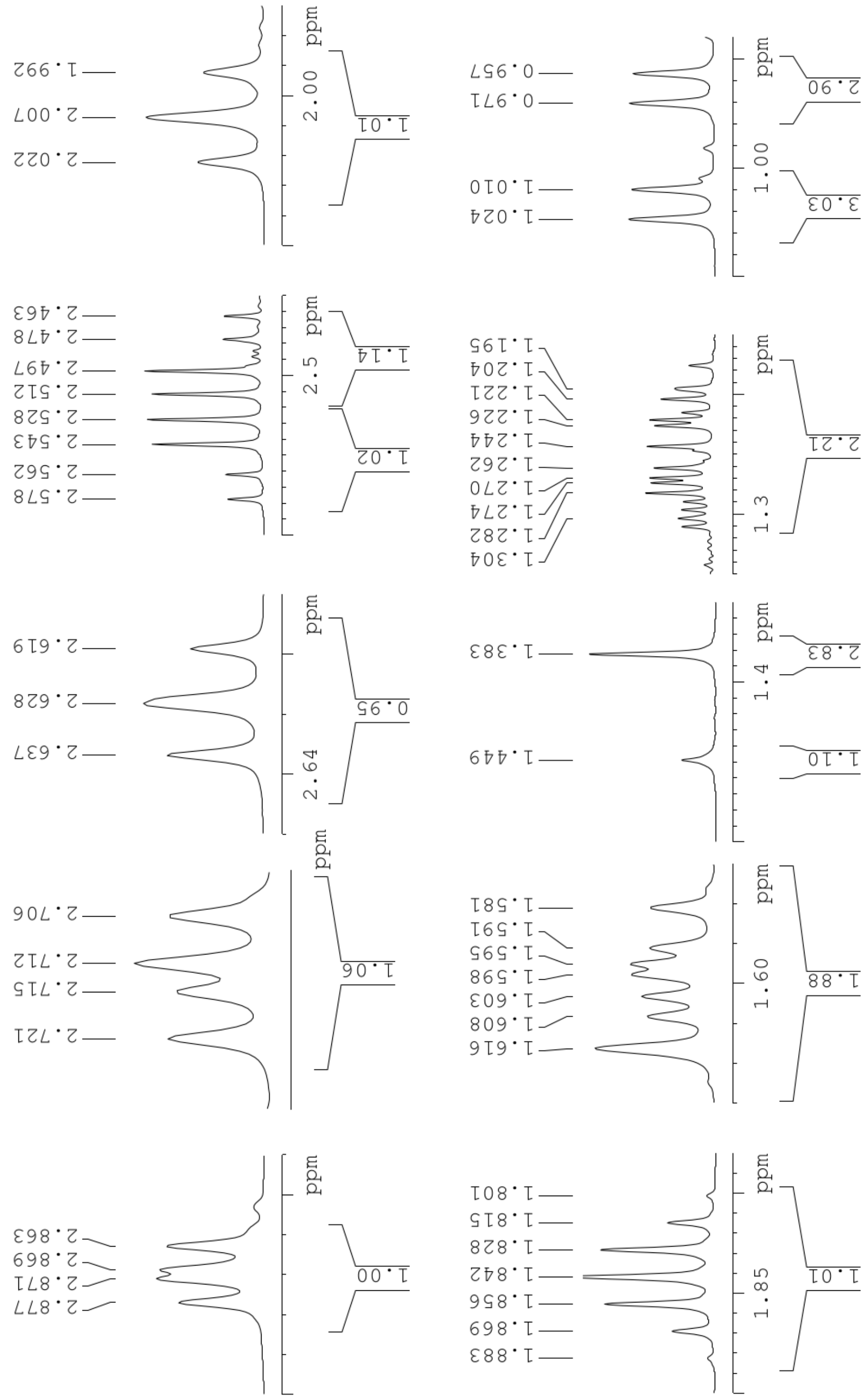

4.20 $^{13}C\{^1H\}$ NMR Spectrum of (±)-11

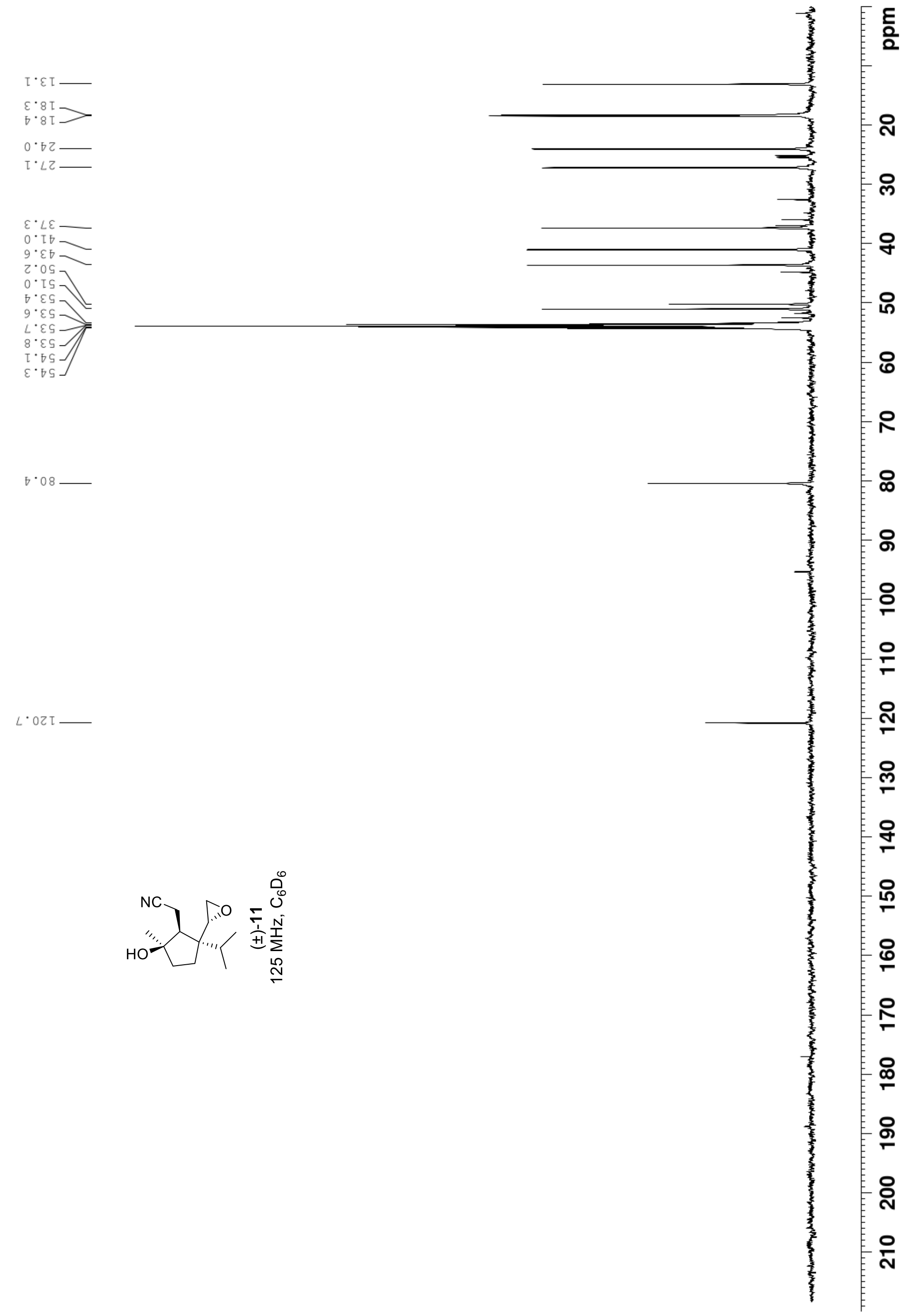

4.21 ^{1}H NMR Spectrum of (±)-4a

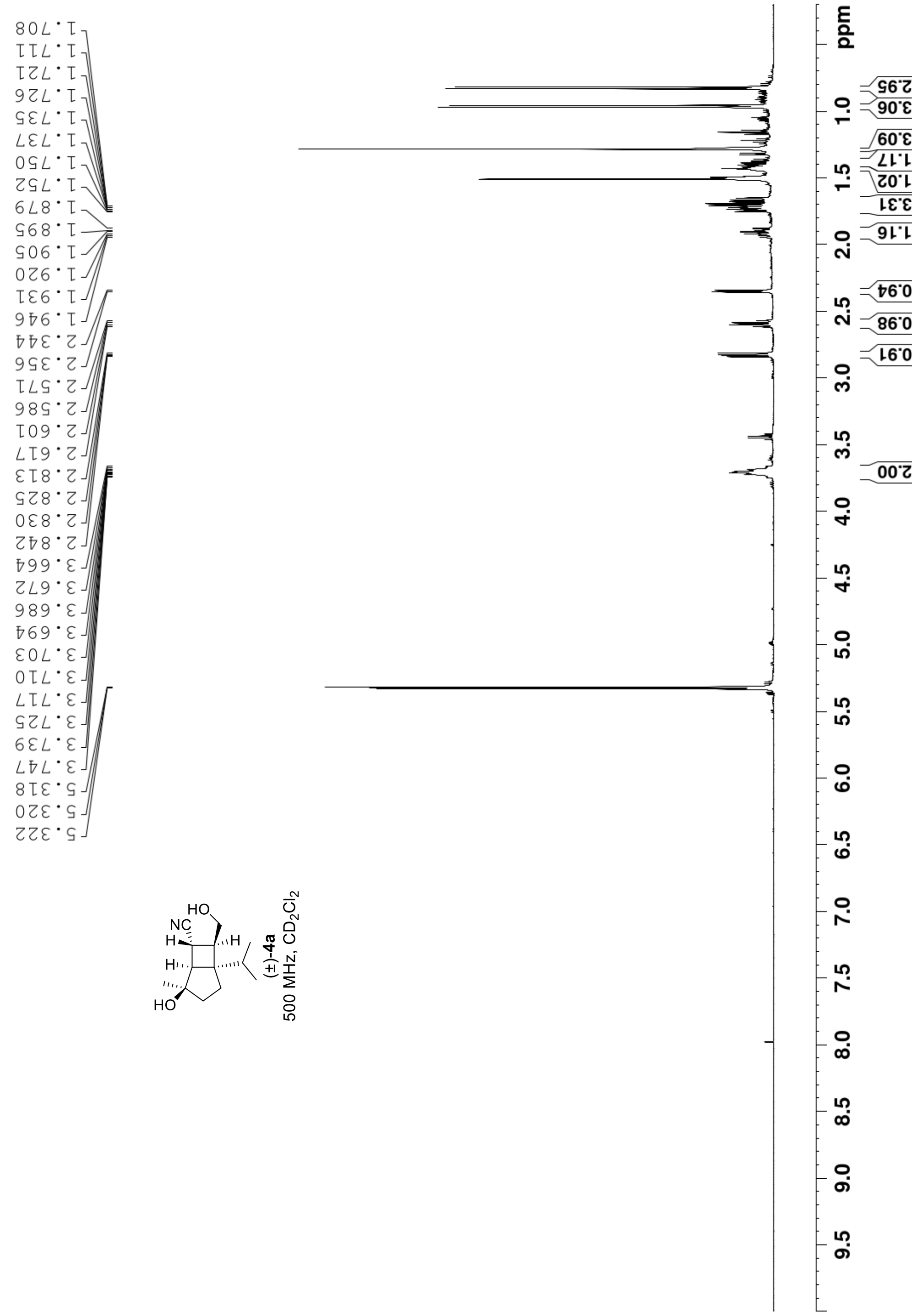

4.22 ^{1}H NMR Spectrum of (±)-4a (Expansion)

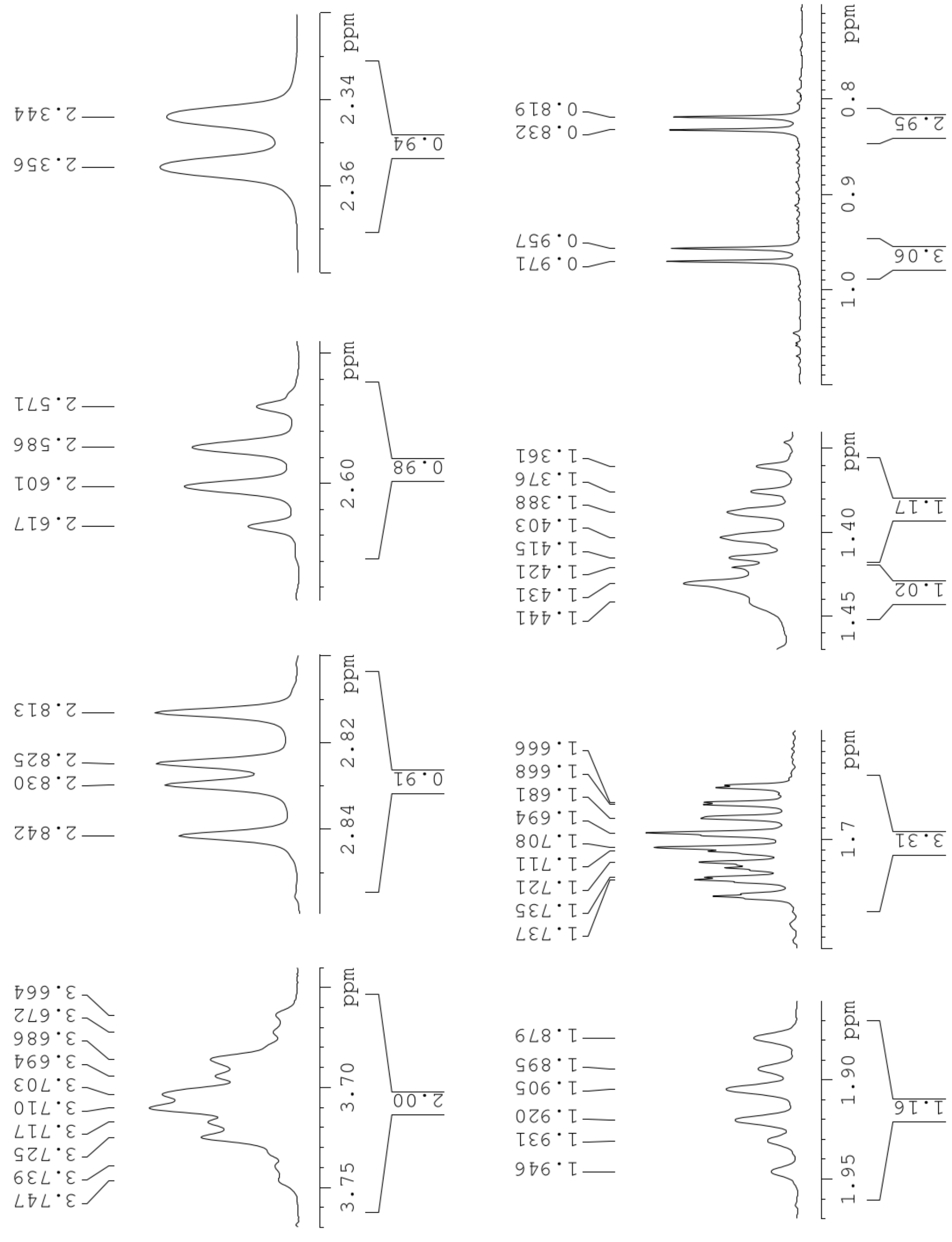

4.23 $^{13}C\{^{1}H\}$ NMR Spectrum of (±)-4a

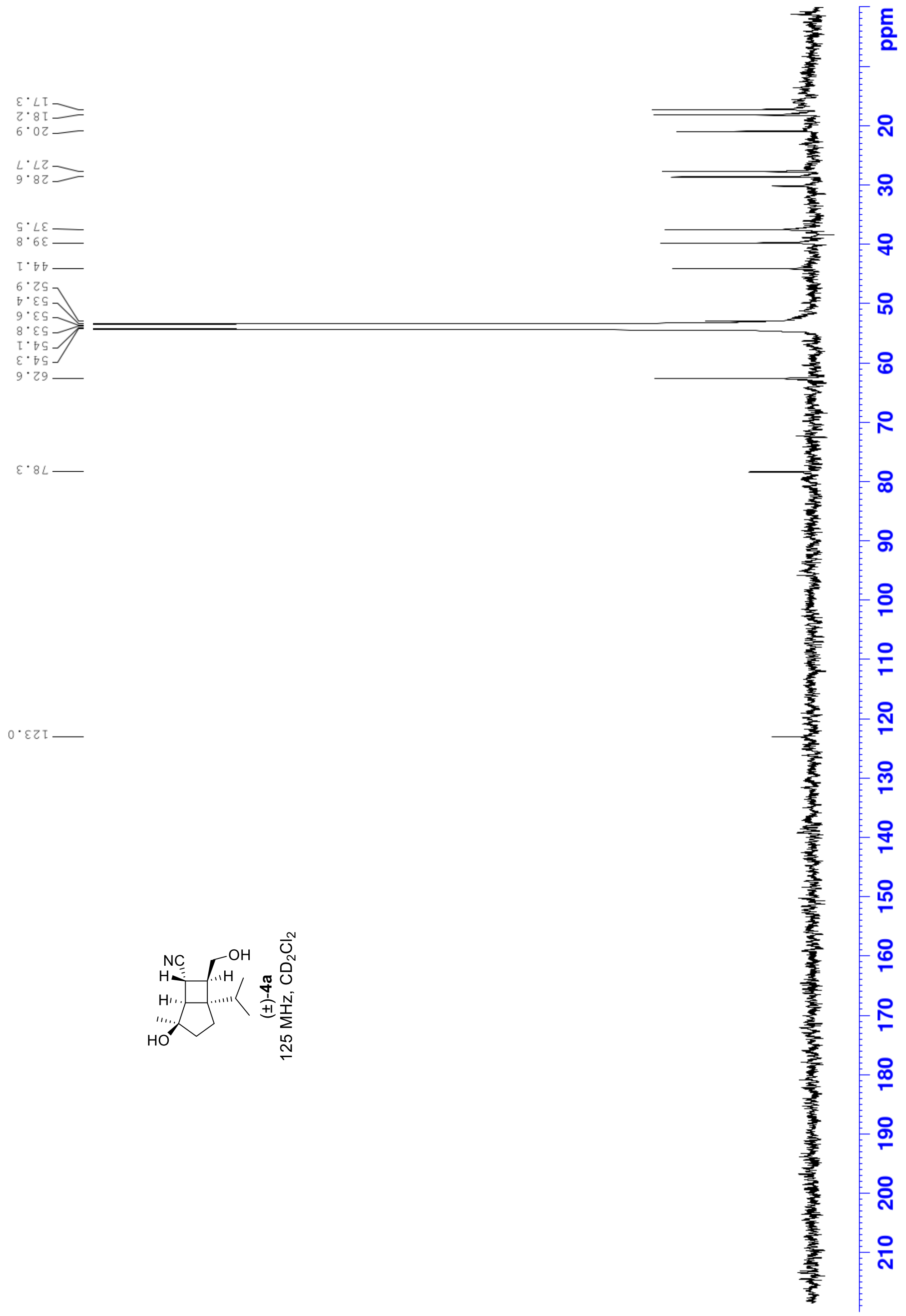

4.24 ^{1}H NMR Spectrum of 12

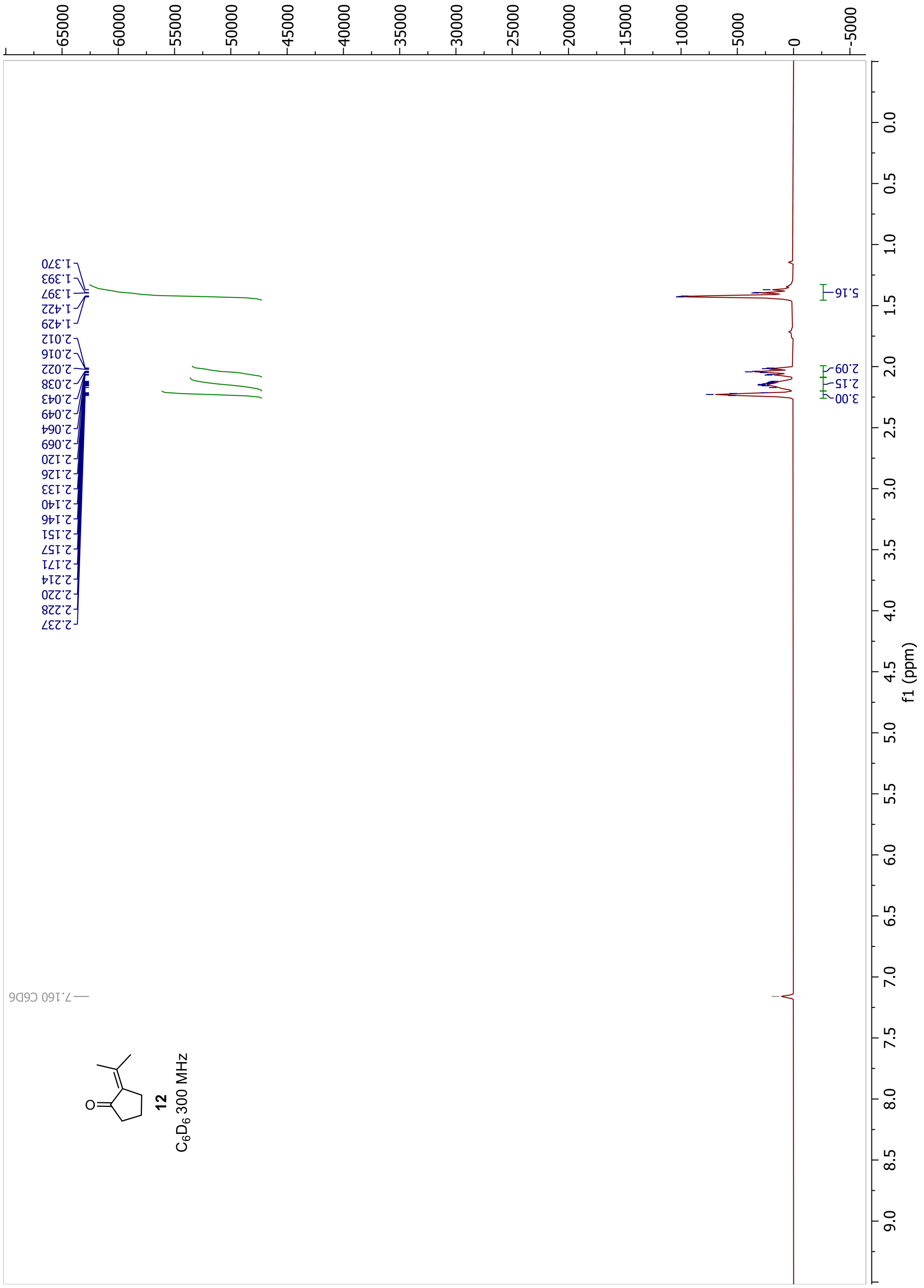

4.25 $^{13}C\{^1H\}$ NMR Spectrum of 12

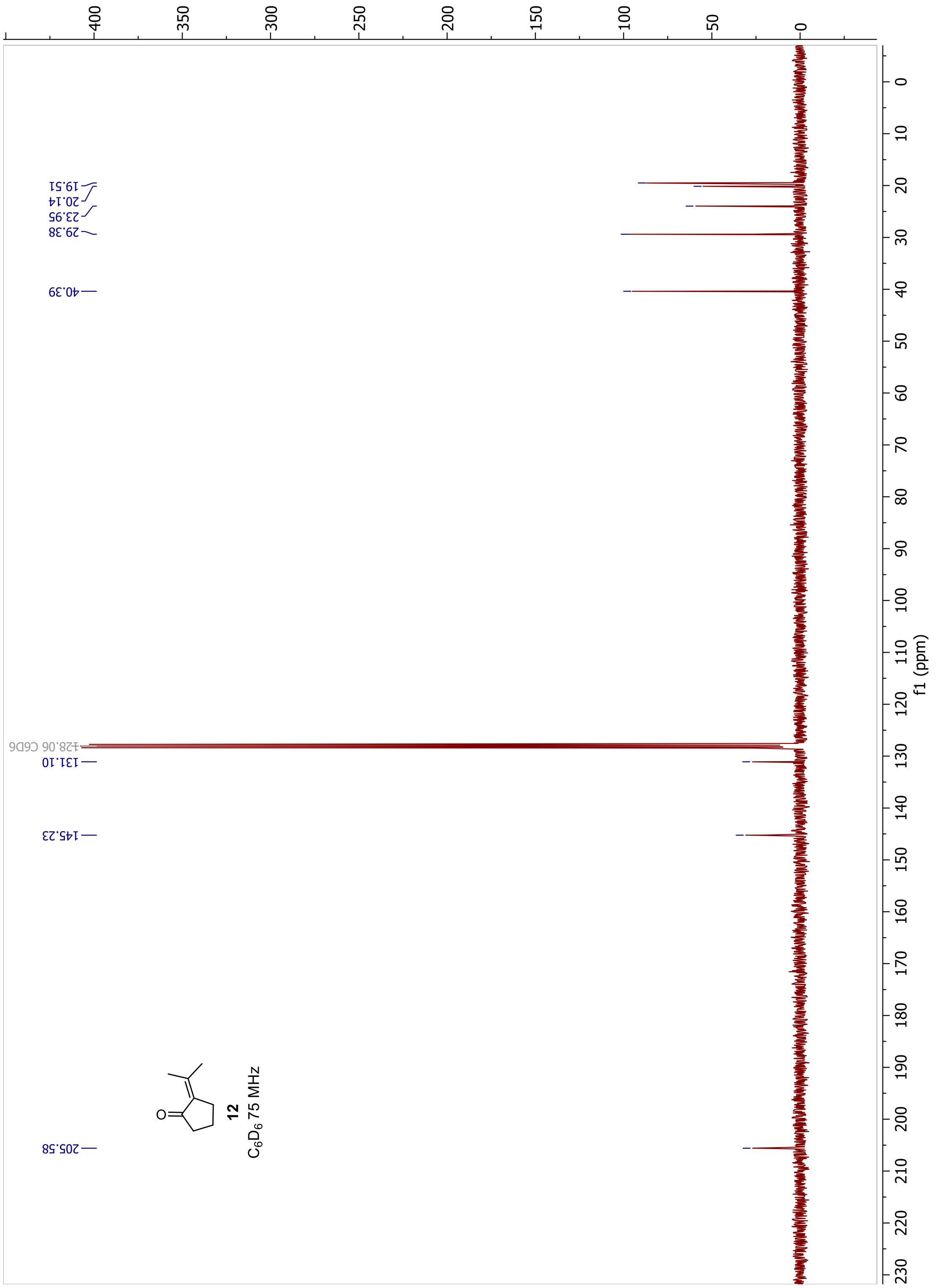

4.26 ^{1}H NMR Spectrum of 13

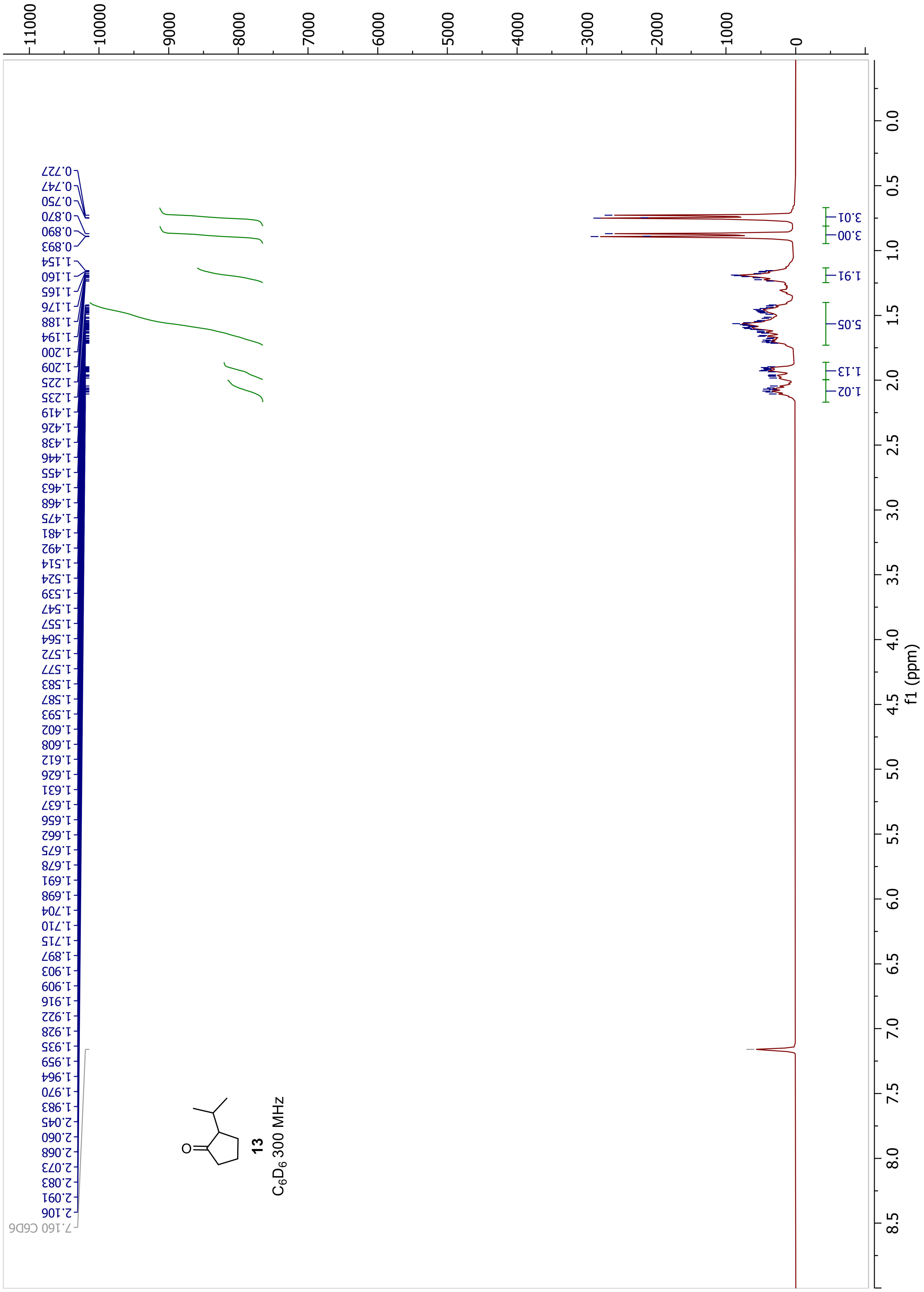

4.27 $^{13}C\{^{1}H\}$ NMR Spectrum of 13

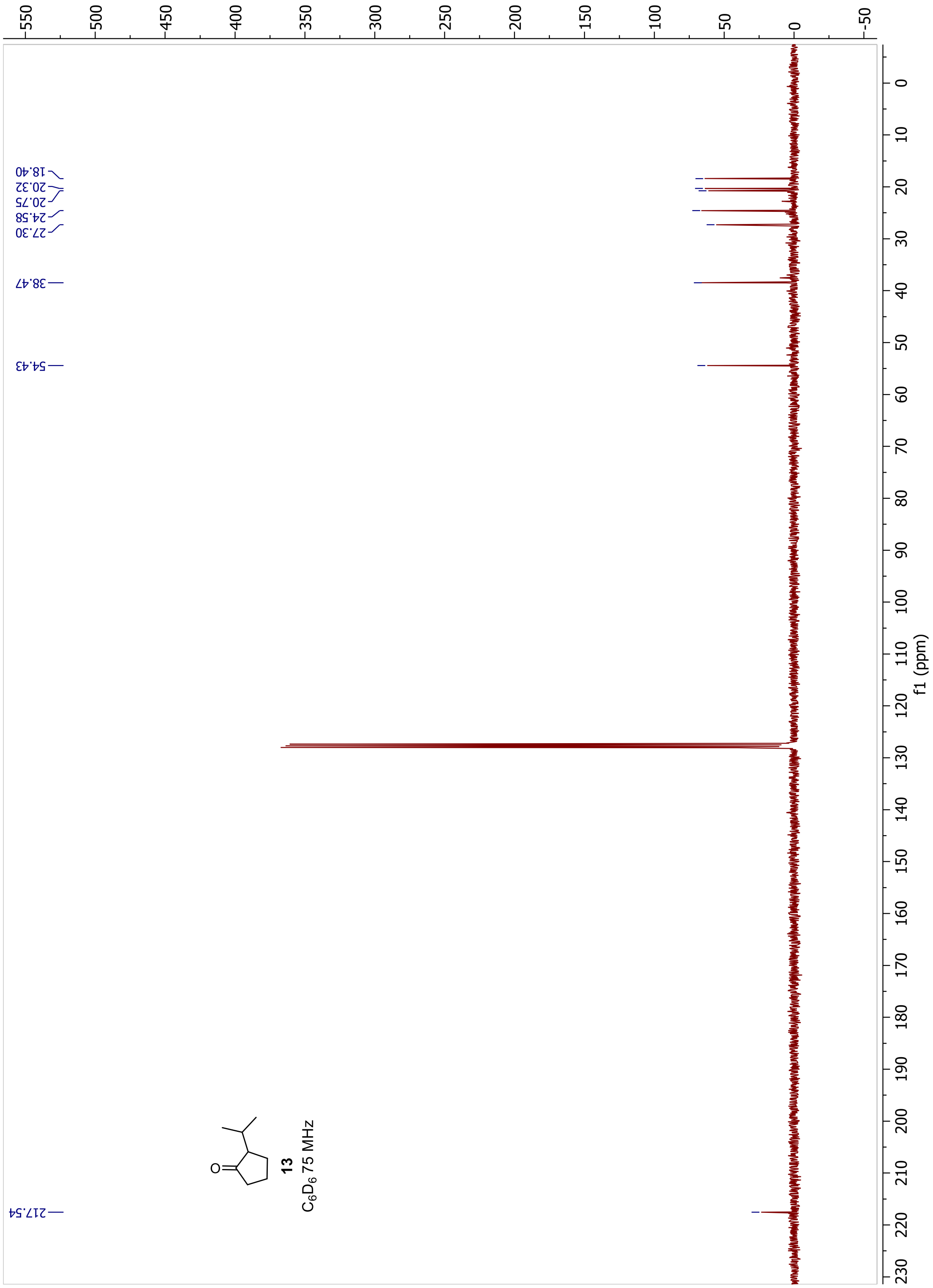

4.28 ^{1}H NMR Spectrum of (±)-15

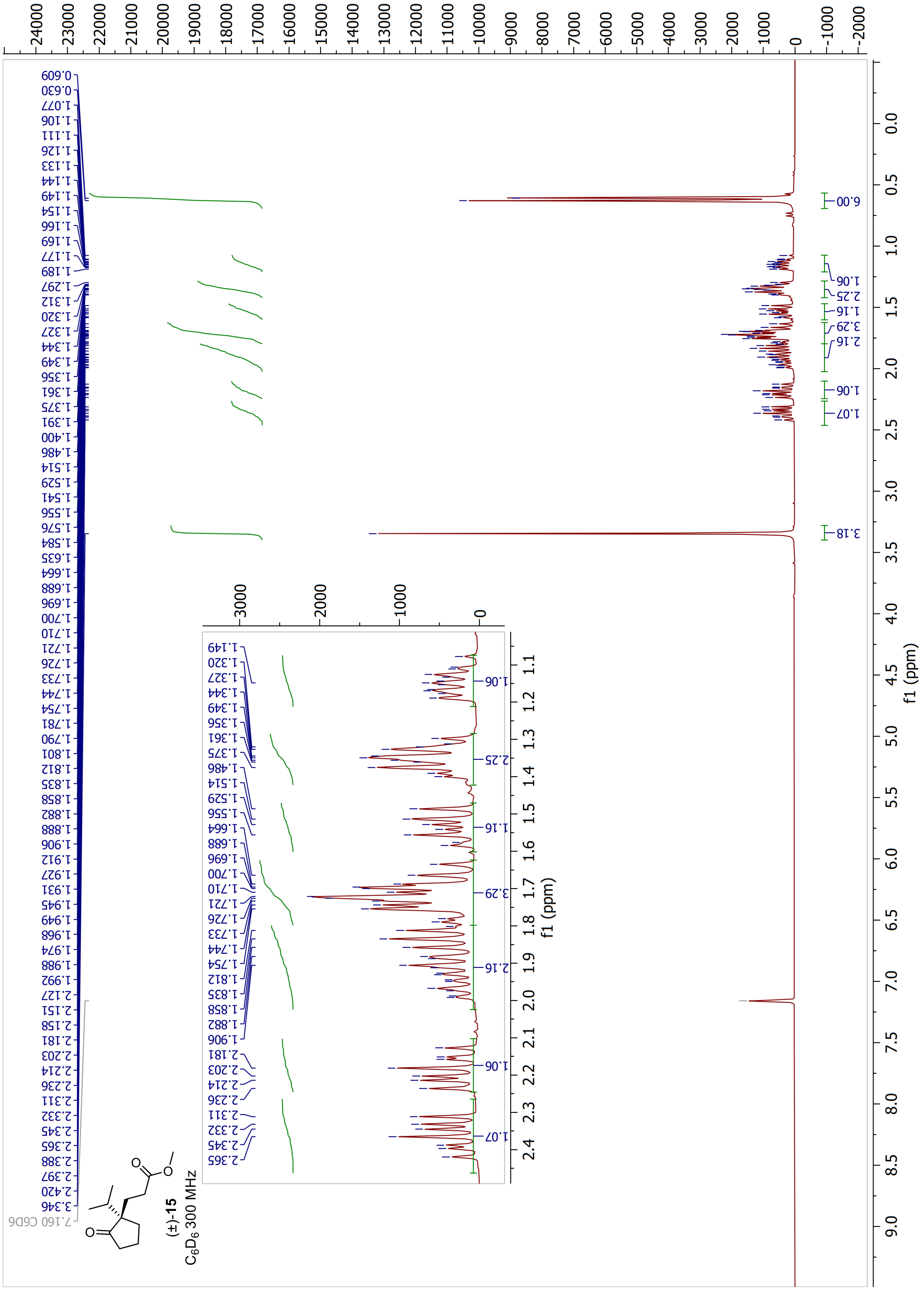

4.29 $^{13}C\{^{1}H\}$ NMR Spectrum of (±)-15

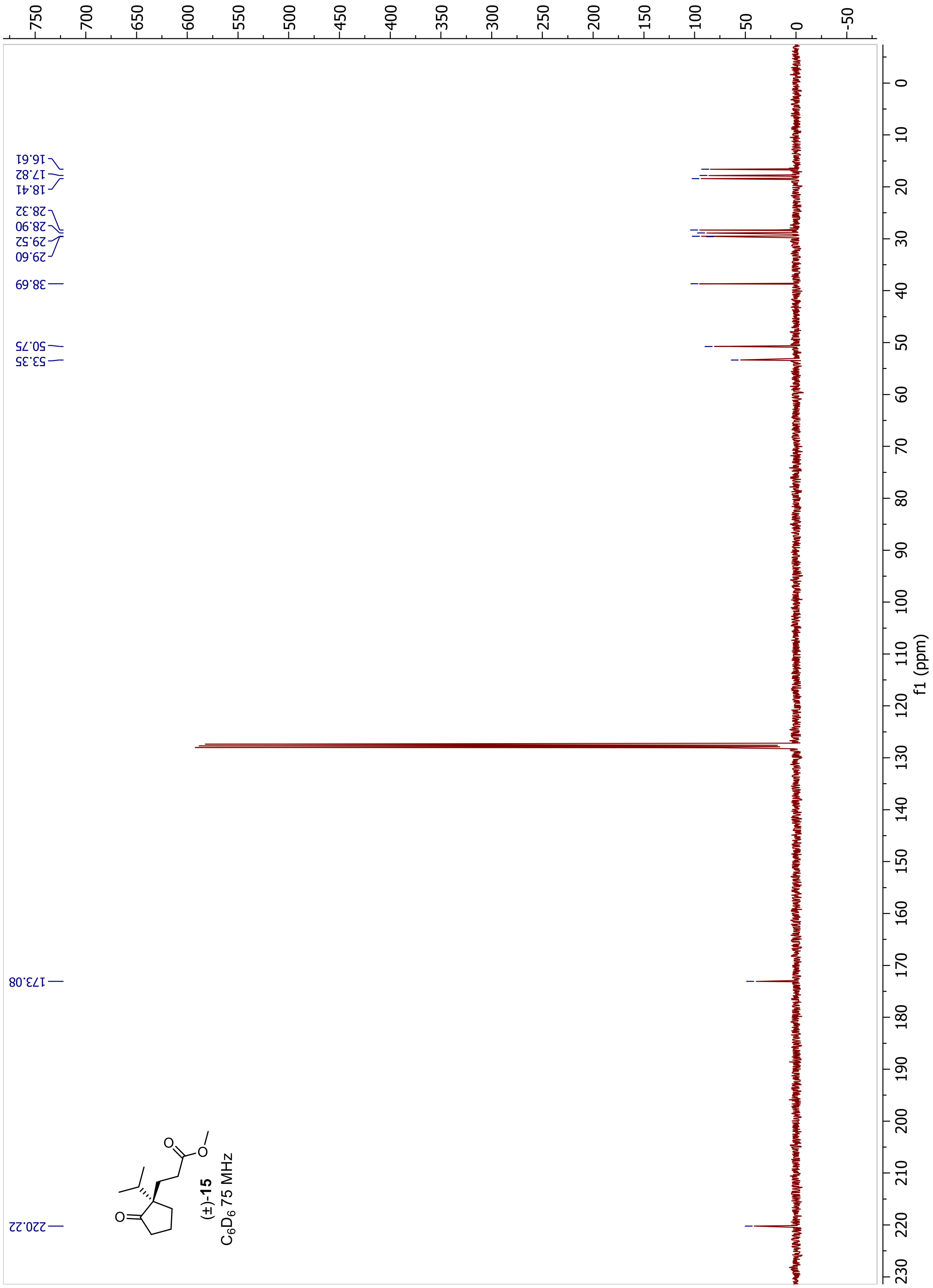

4.30 ^{1}H NMR Spectrum of (–)-15

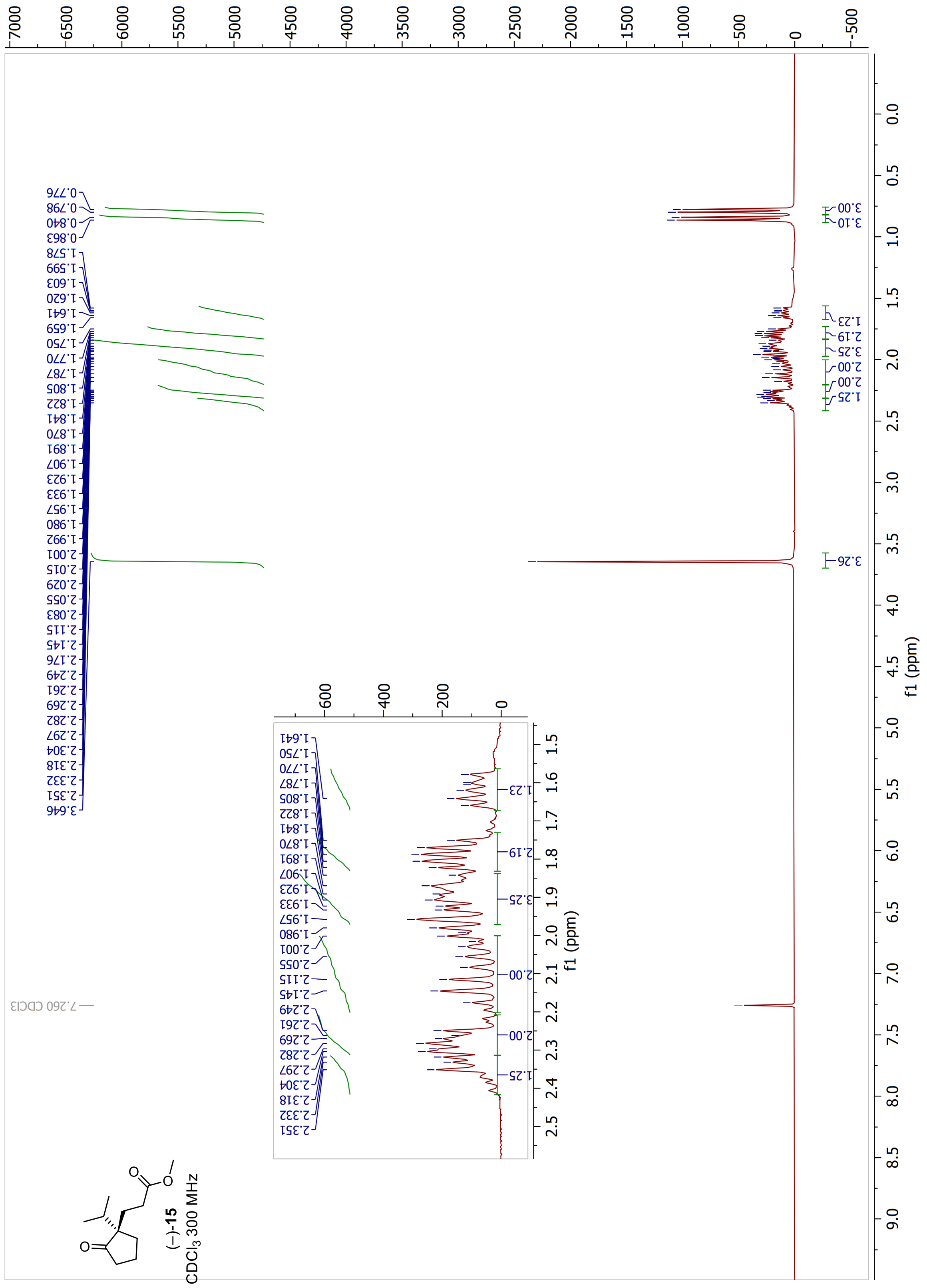

4.31 ^{1}H NMR Spectrum of (±)-16

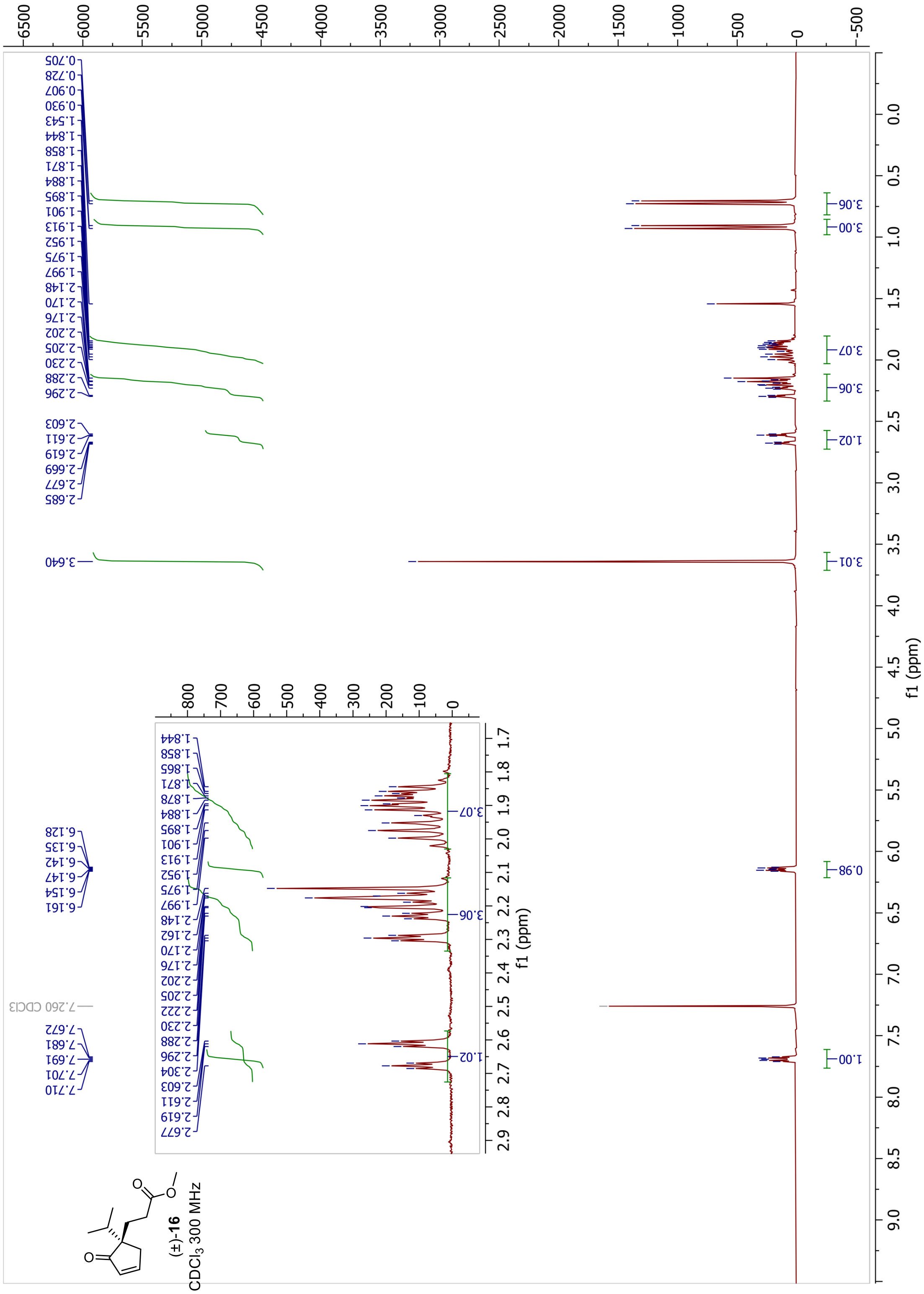

4.32 $^{13}C\{^{1}H\}$ NMR Spectrum of (±)-16

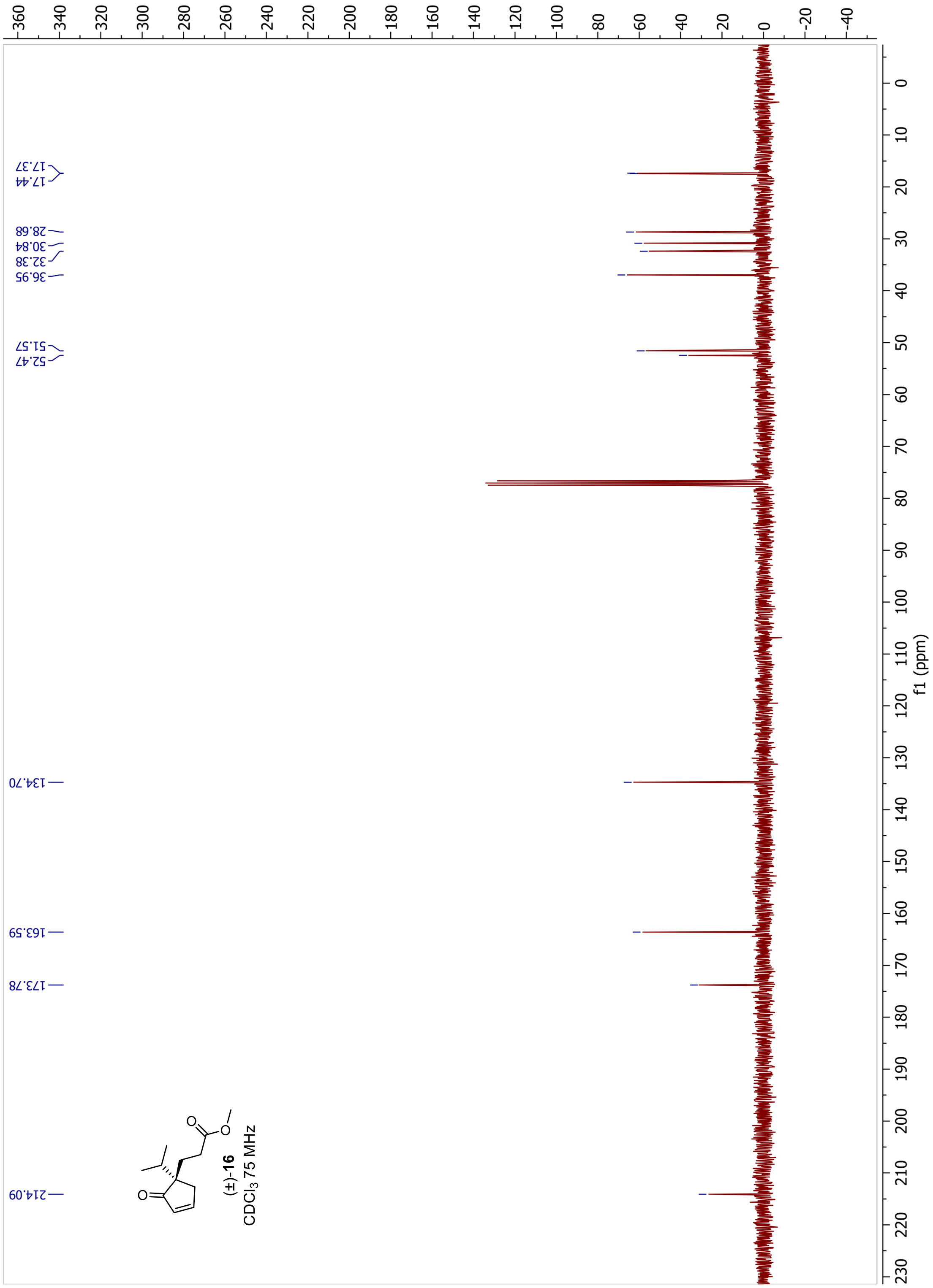

4.33 ¹H NMR Spectrum of (–)-16

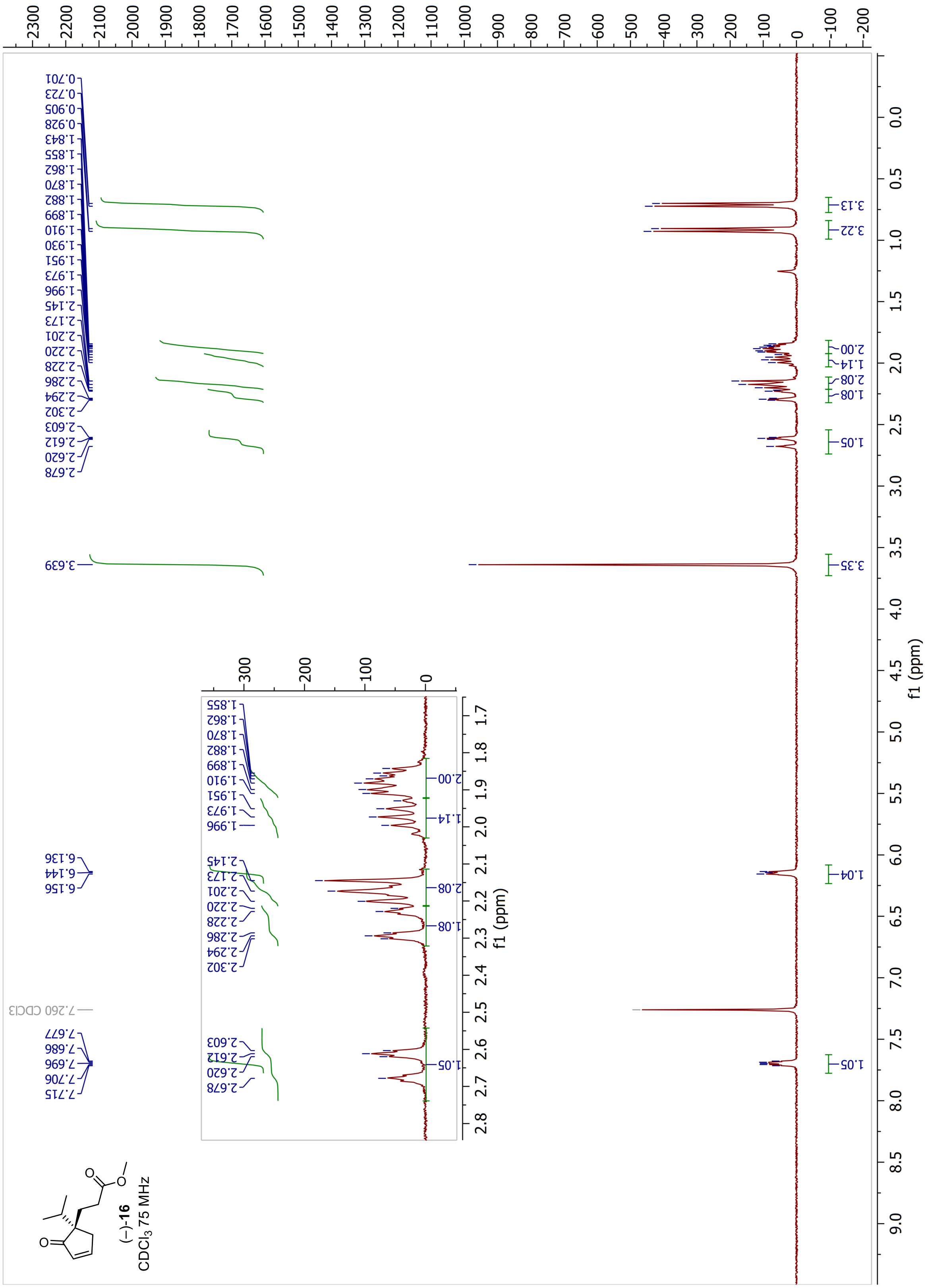

4.34 ^{1}H NMR Spectrum of (±)-18

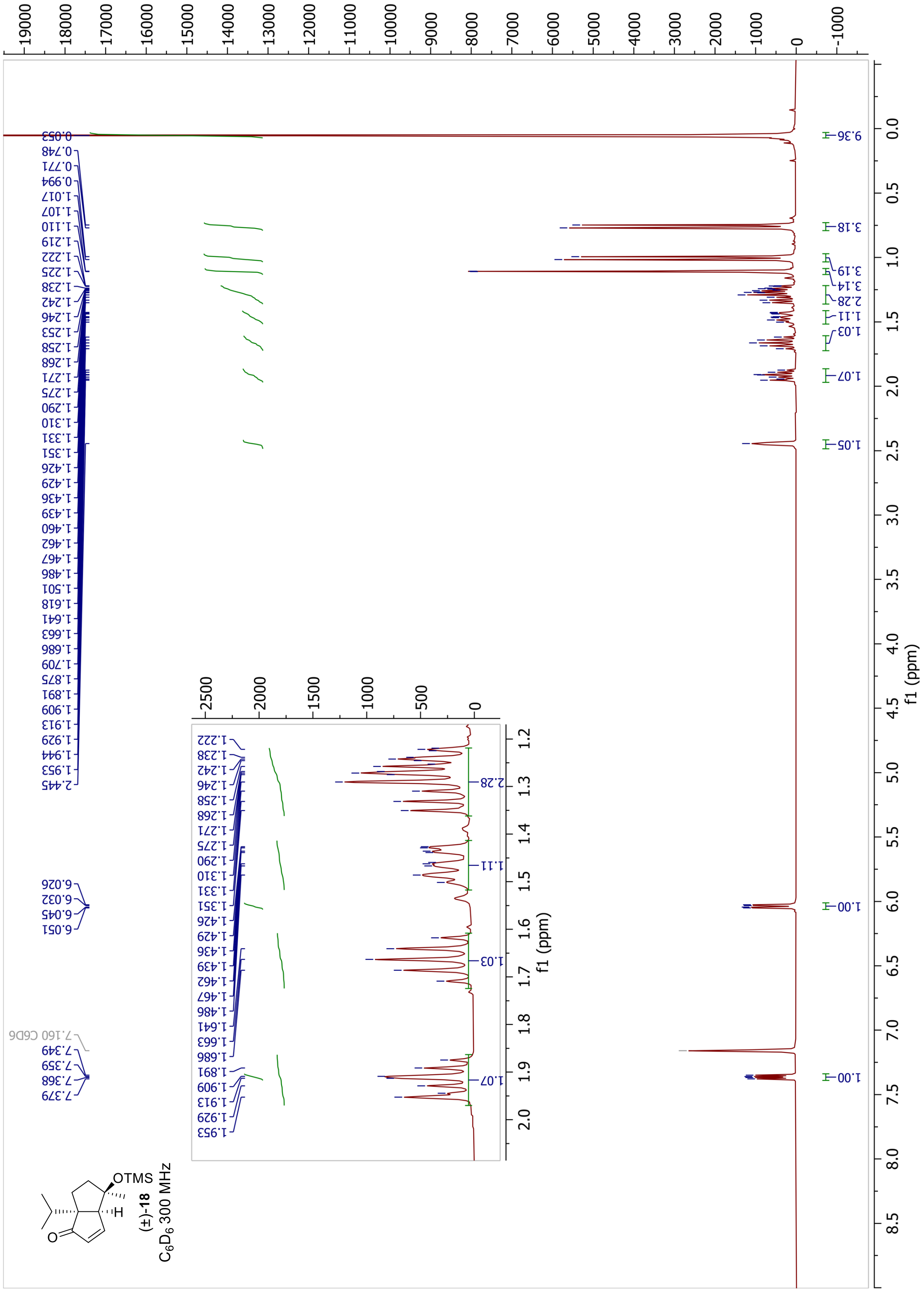

4.35 $^{13}C\{^{1}H\}$ NMR Spectrum of (±)-18

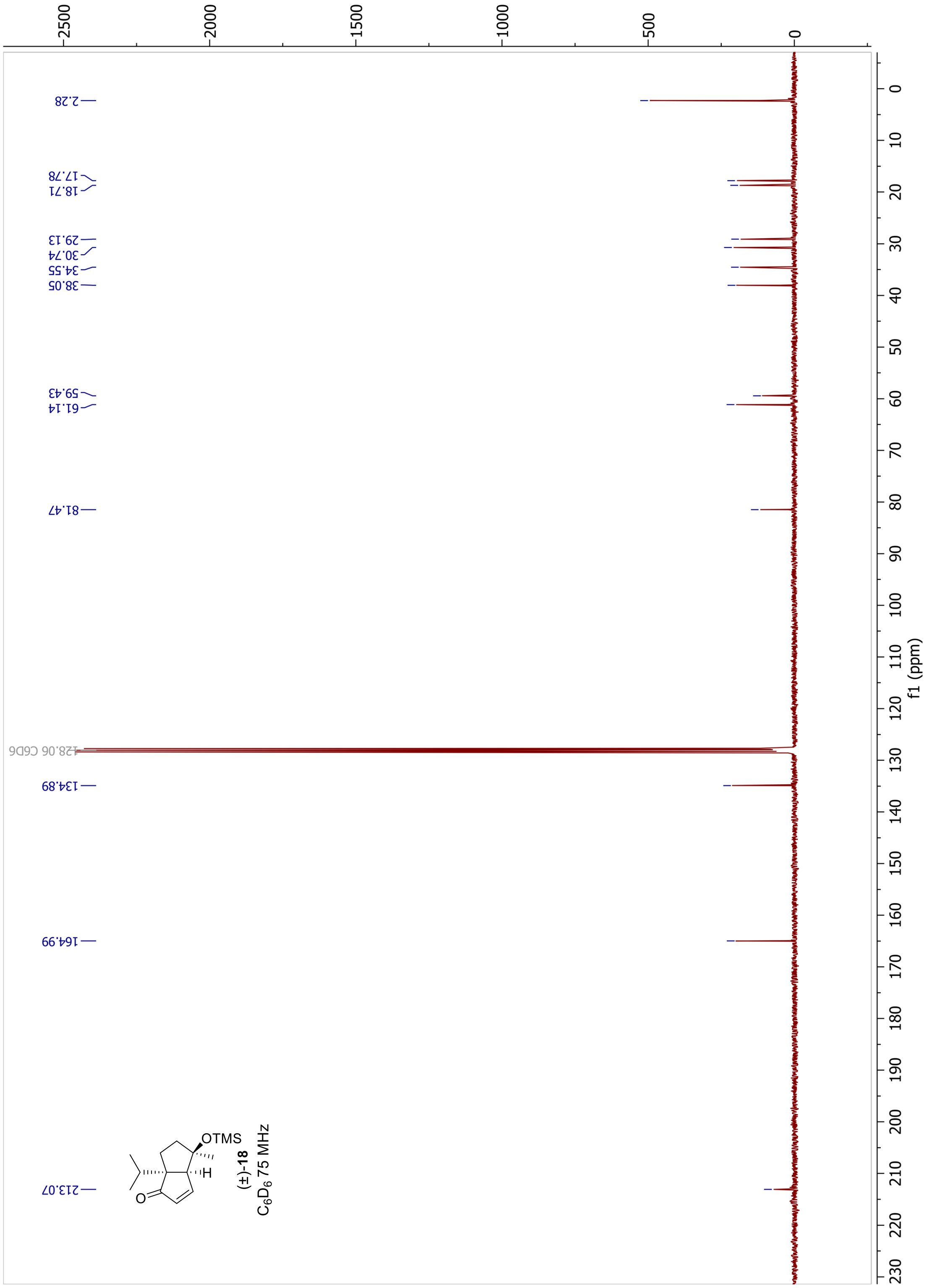

4.36 ¹H NMR Spectrum of (±)-20

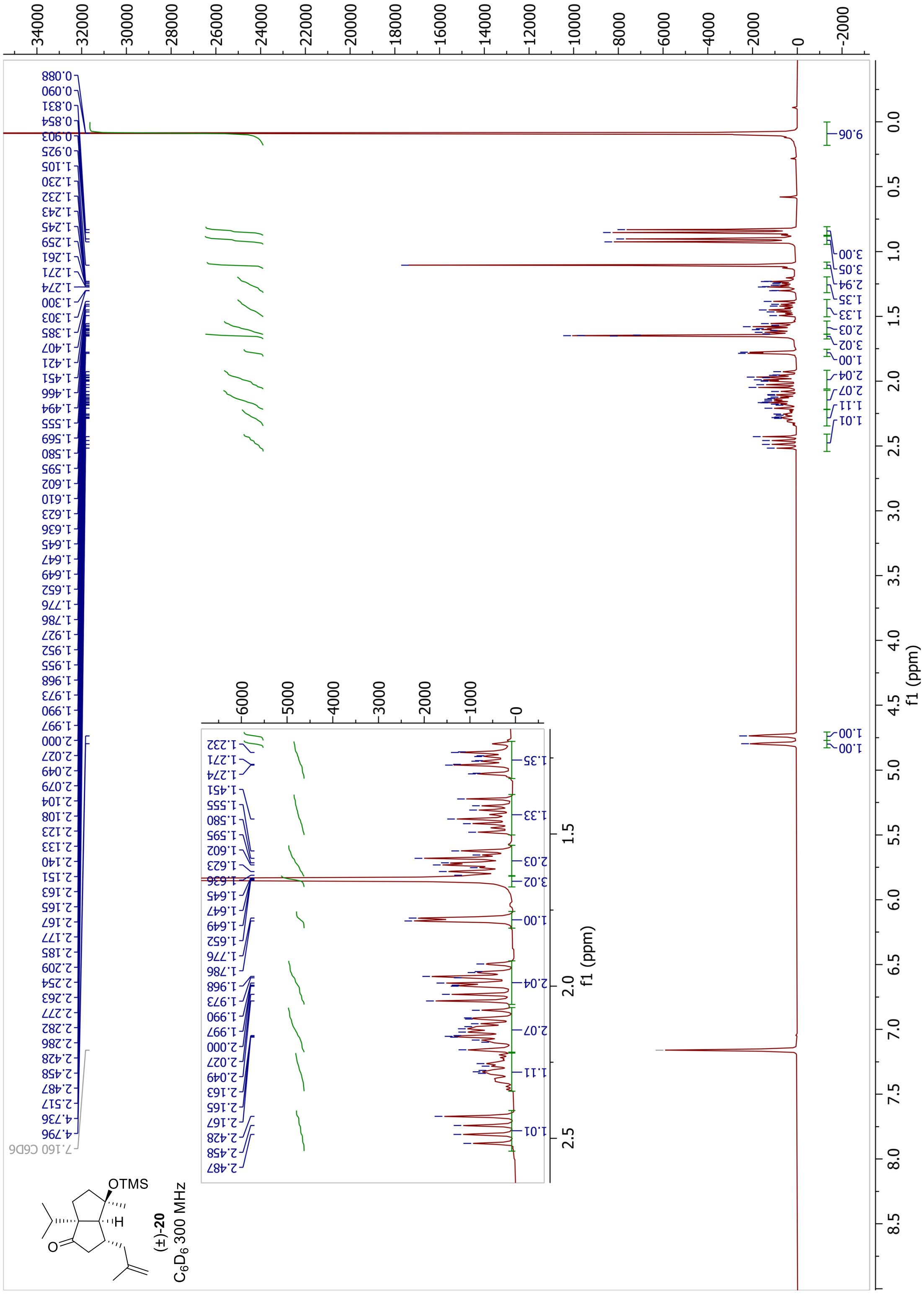

4.37 $^{13}C\{^{1}H\}$ NMR Spectrum of (±)-20

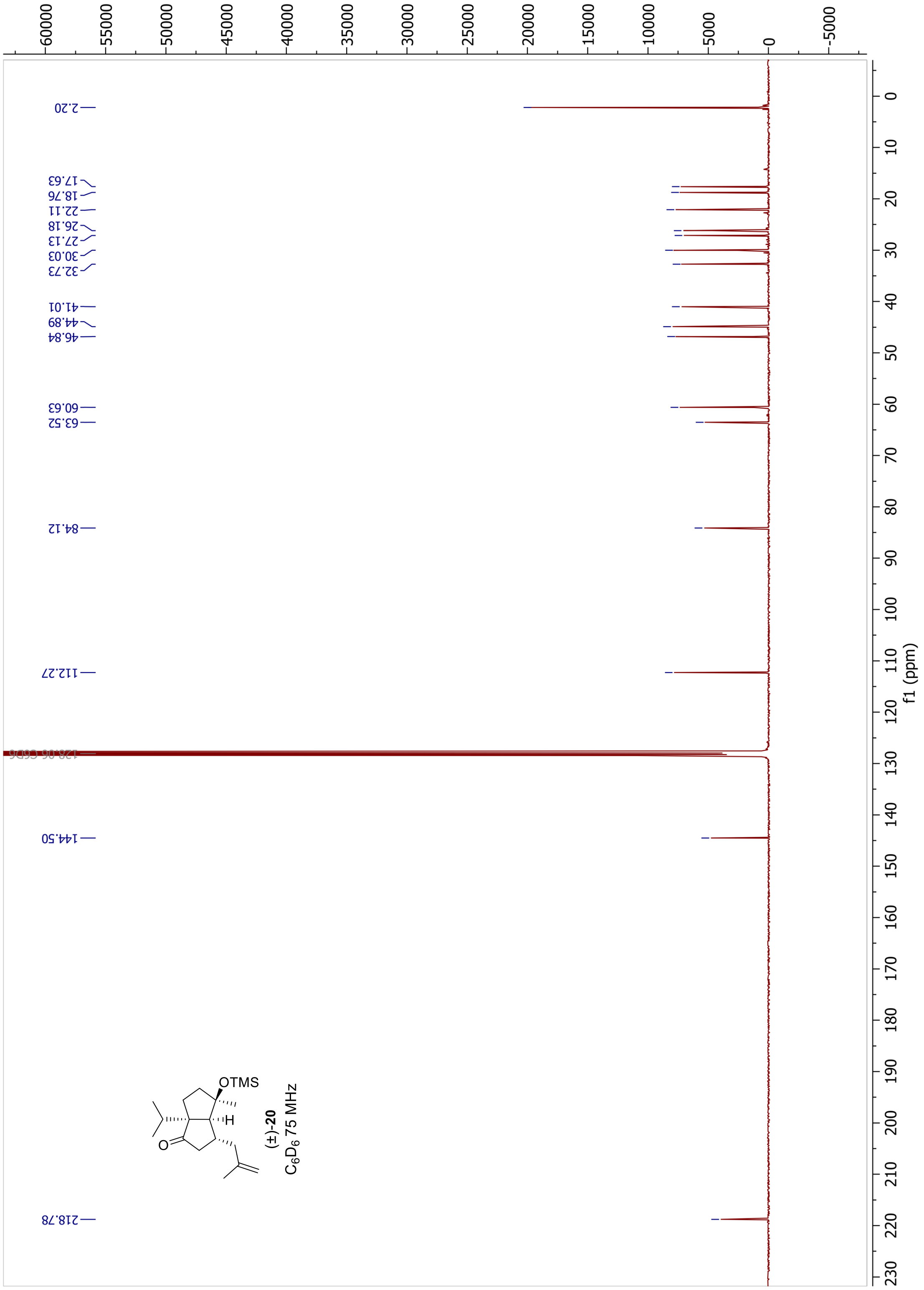

4.38 ^{1}H NMR Spectrum of (±)-21

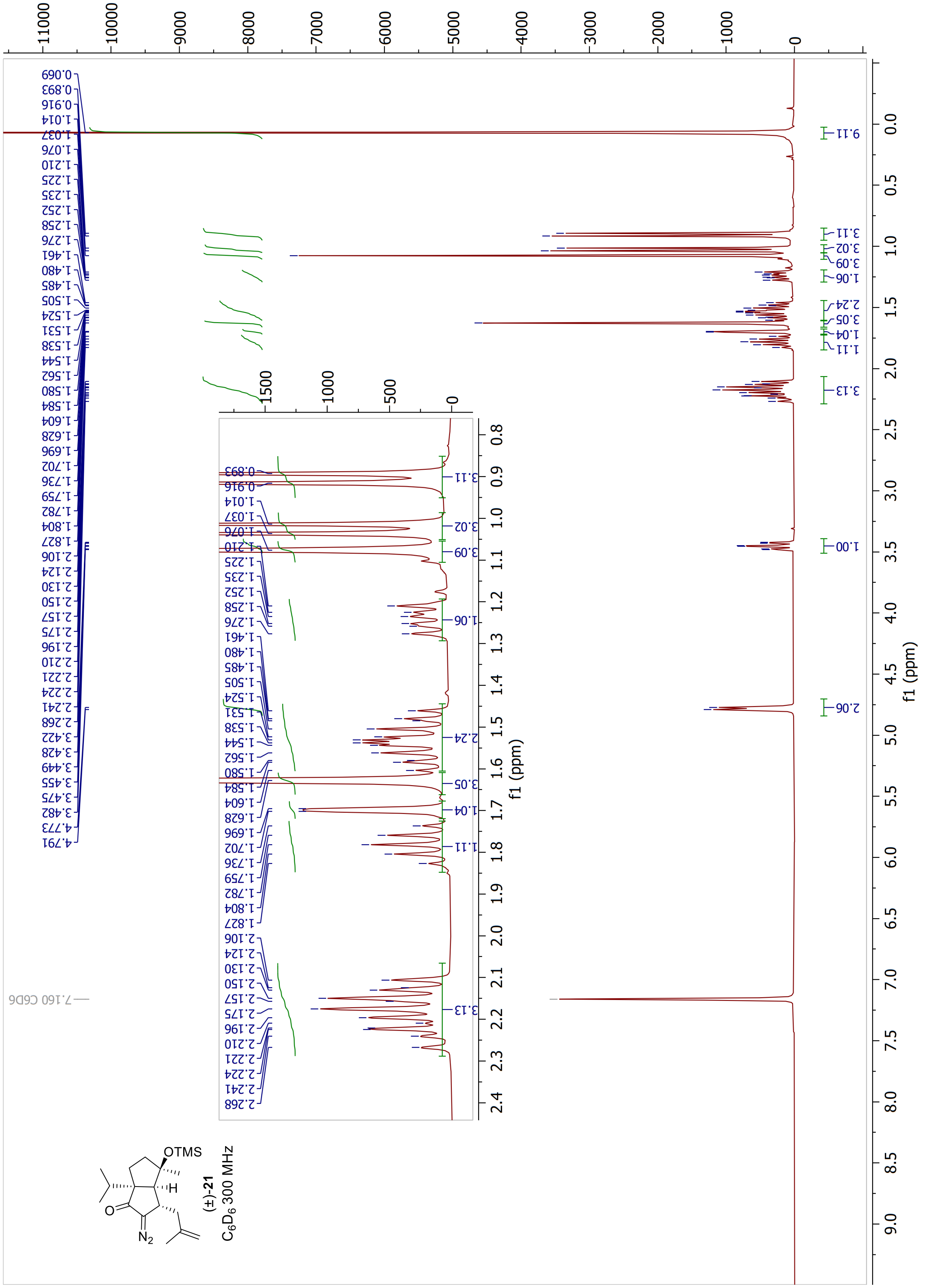

4.39 $^{13}C\{^{1}H\}$ NMR Spectrum of (±)-21

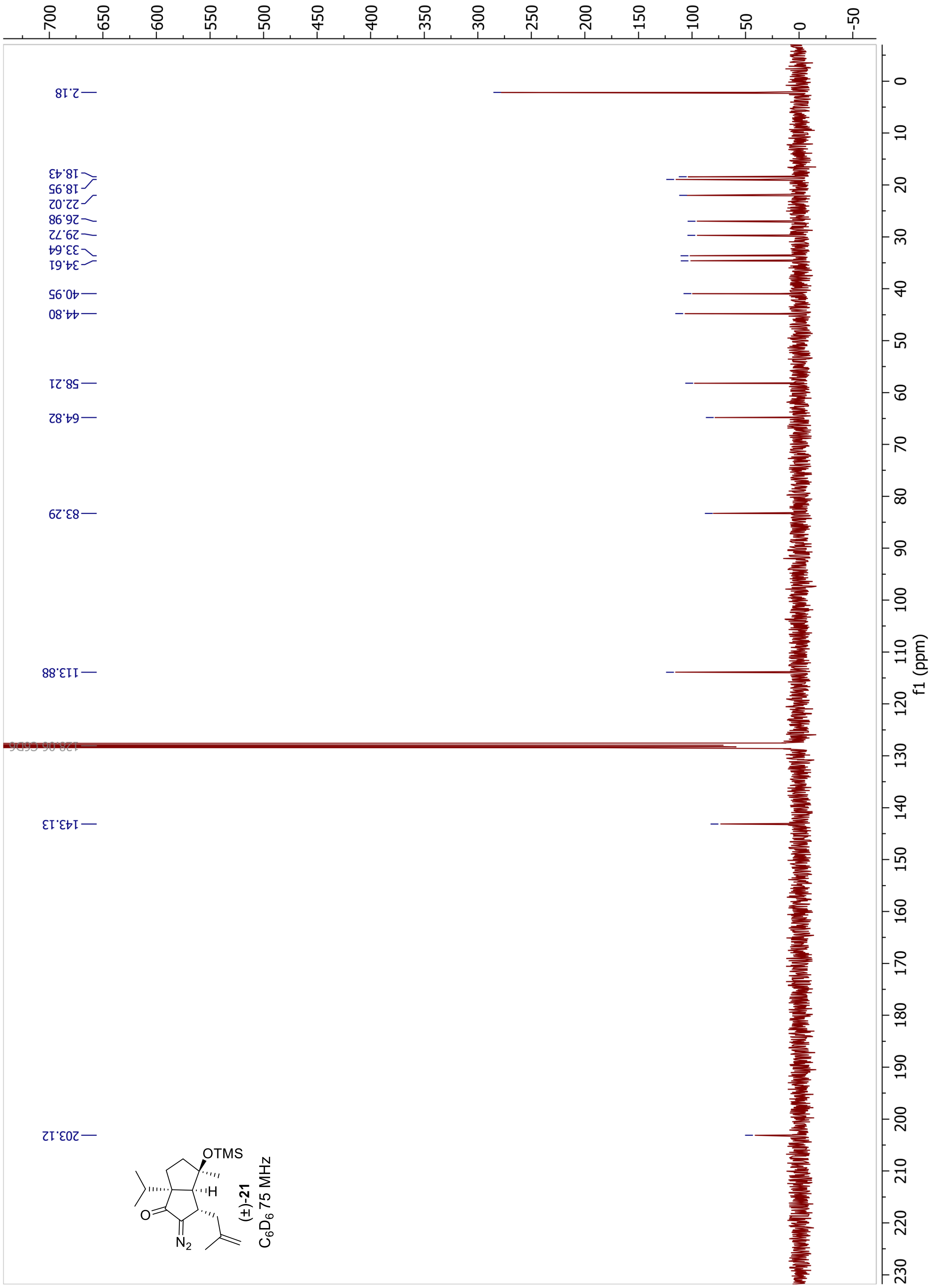

4.40 ^{1}H NMR Spectrum of (±)-4b

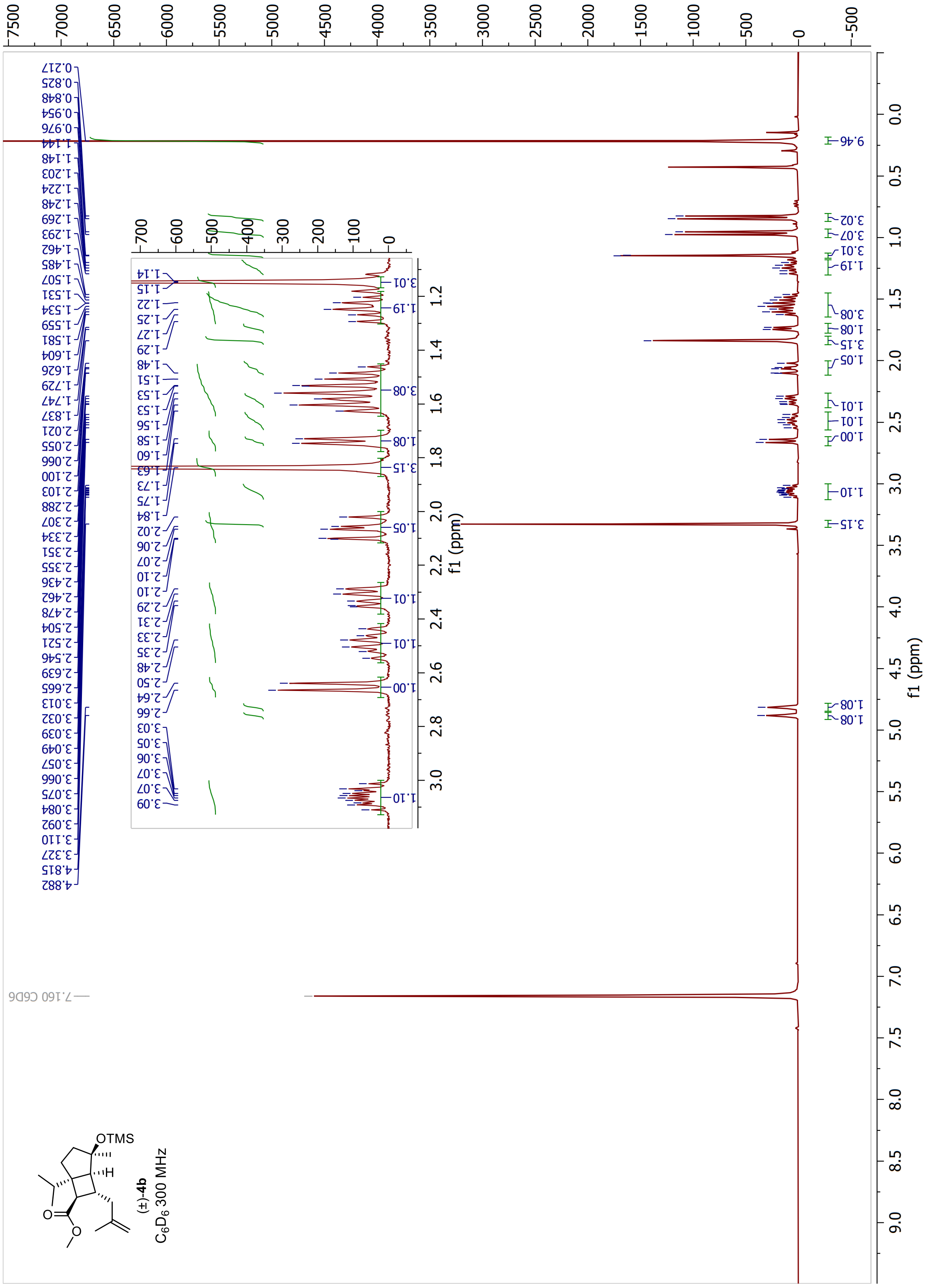

4.41 $^{13}C\{^{1}H\}$ NMR Spectrum of (±)-4b

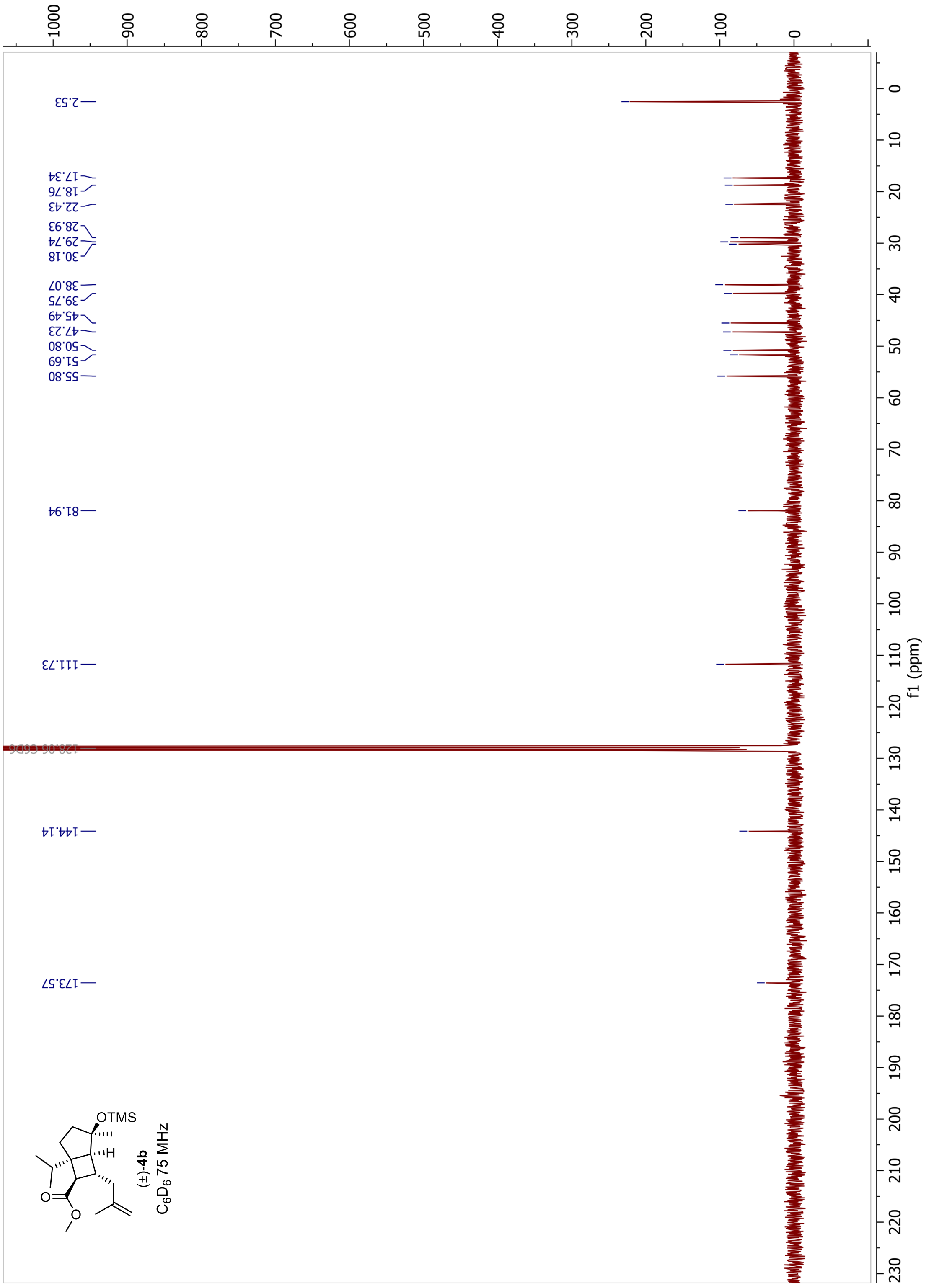

4.42 135DEPT NMR Spectrum of (±)-4b

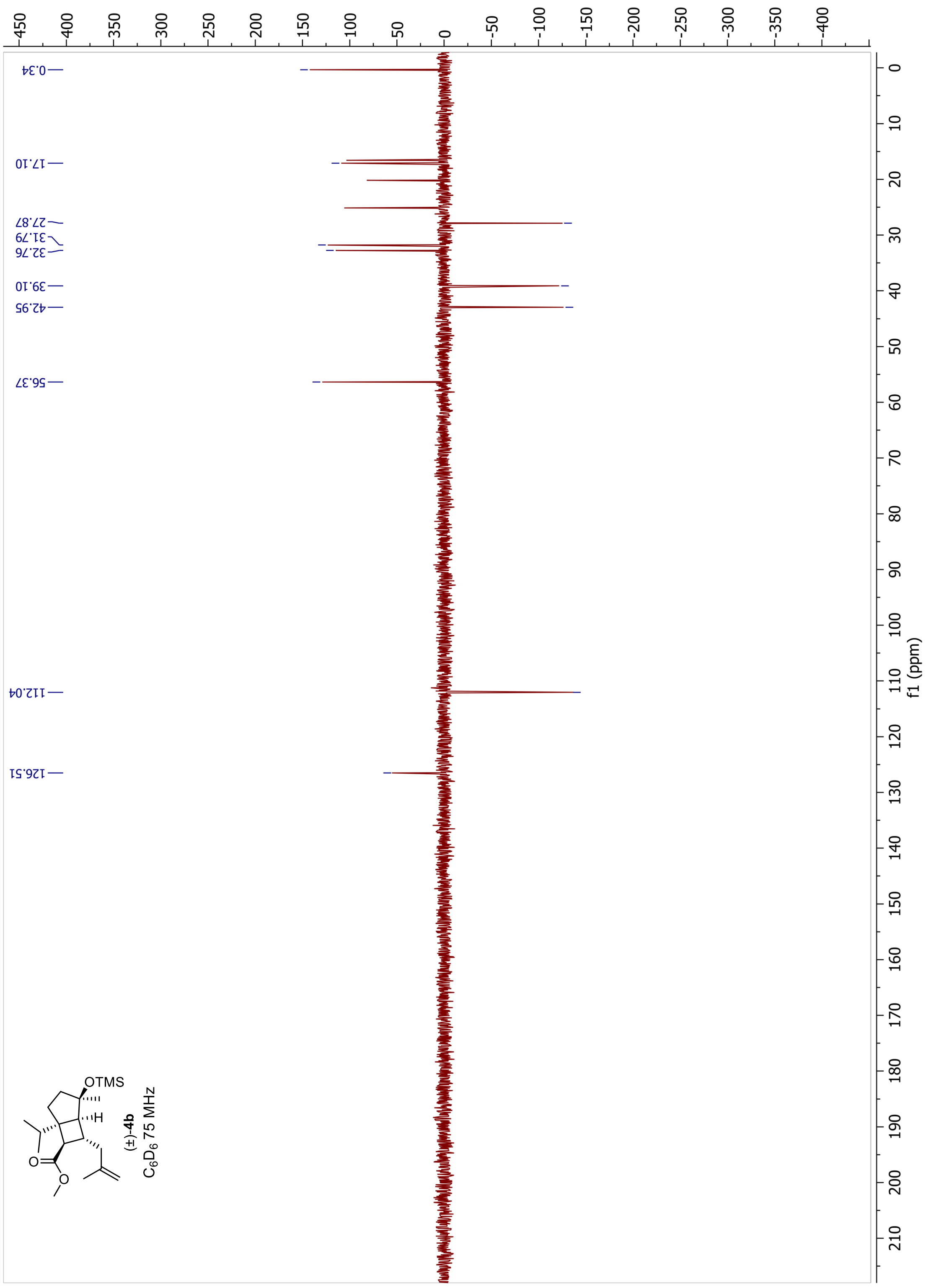

4.43 GC chromatogram for (±)-16

```
ata File C:\CHEM32\1\DATA\AINO\VEERA 2024-01-25 13-28-16\201B0101.D
ample Name: ENONE RACEMIC

=====================================================================
Acq. Operator    : AINO                              Seq. Line :    1
Acq. Instrument  : Instrument 1                       Location : Vial 201
Injection Date   : 1/25/2024 1:32:33 PM                    Inj :    1
                                                    Inj Volume : 0.2 µl
Acq. Method      : C:\CHEM32\1\DATA\AINO\VEERA 2024-01-25 13-28-16\AINO 120C 30MIN INL250
                   SPLIT50 B-DM.M
Last changed     : 1/24/2024 3:39:24 PM by VEERA
Analysis Method  : C:\CHEM32\1\METHODS\AINO 120C 30MIN INL250 SPLIT50 B-DM.M
Last changed     : 1/24/2024 3:39:24 PM by VEERA
Additional Info  : Peak(s) manually integrated
```

Current Chromatogram(s)

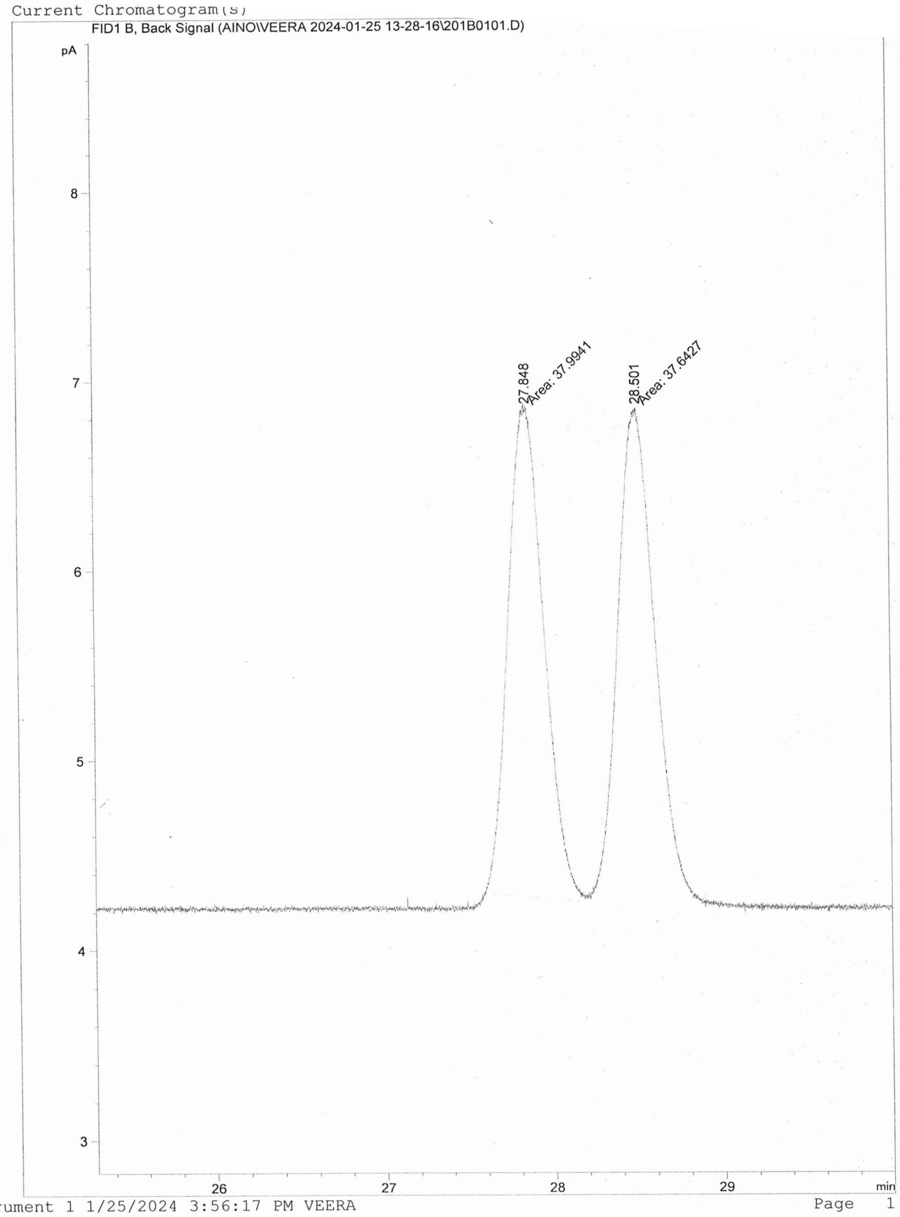

Instrument 1 1/25/2024 3:56:17 PM VEERA Page 1

```
=====================================================================
                        Area Percent Report
=====================================================================

Sorted By             :      Signal
Multiplier:                  :      1.0000
Dilution:                    :      1.0000
Do not use Multiplier & Dilution Factor with ISTDs


Signal 1: FID1 B, Back Signal

Peak RetTime Type  Width     Area      Height     Area
  #   [min]        [min]   [pA*s]      [pA]        %
----|-------|----|-------|----------|----------|--------|
   1   1.534 BB S  0.0170 2.92441e4  2.87020e4  99.74203
   2  27.848 MM    0.2454   37.99413    2.58076  0.12959
   3  28.501 MM    0.2453   37.64270    2.55726  0.12839

Totals :                  2.93198e4  2.87071e4

=====================================================================
```

4.44 GC chromatogram for (–)-16

```
Data File C:\CHEM32\1\DATA\VEERA\VEERA 2024-01-26 09-08-29\202B0401.D
Sample Name: CHIRAL ENONE

    =================================================================
    Acq. Operator    : VEERA                          Seq. Line :   4
    Acq. Instrument  : Instrument 1                    Location : Vial 202
    Injection Date   : 1/26/2024 10:11:46 AM                Inj :   1
                                                     Inj Volume : 0.2 µl
    Acq. Method      : C:\CHEM32\1\DATA\VEERA\VEERA 2024-01-26 09-08-29\AINO 120C 30MIN INL250
                       SPLIT50 B-DM.M
    Last changed     : 1/24/2024 3:39:24 PM by VEERA
    Analysis Method  : C:\CHEM32\1\METHODS\AINO 120C 30MIN INL250 SPLIT50 B-DM.M
    Last changed     : 1/24/2024 3:39:24 PM by VEERA
    Additional Info  : Peak(s) manually integrated
```

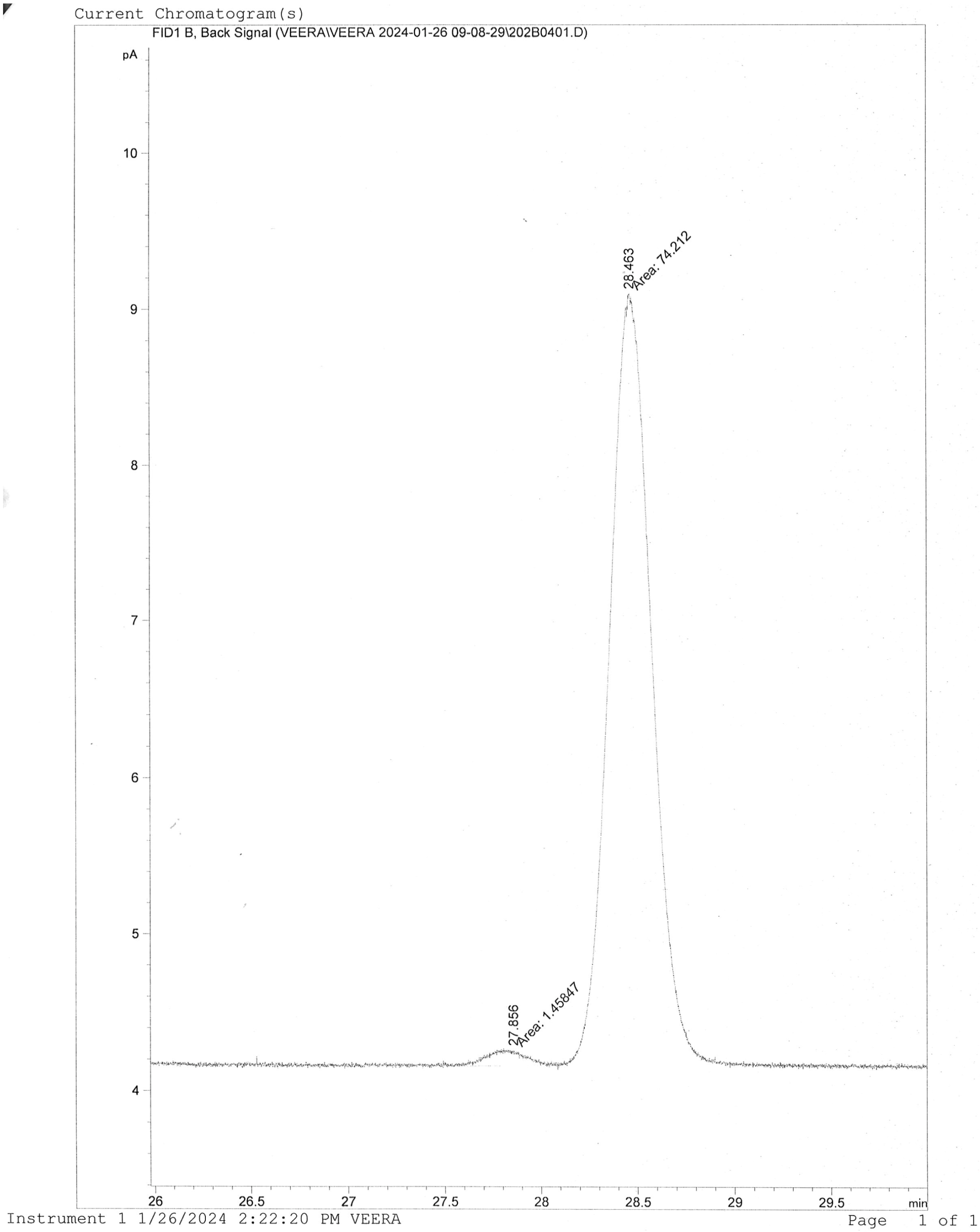
Current Chromatogram(s)
FID1 B, Back Signal (VEERA\VEERA 2024-01-26 09-08-29\202B0401.D)
pA
10
9
8
7
6
5
4
28.463
Area: 74.212
27.856
Area: 1.45847
26
26.5
27
27.5
28
28.5
29
29.5
min
Instrument 1 1/26/2024 2:22:20 PM VEERA
Page 1 of 1

Area Percent Report

Sorted By : Signal
Multiplier: : 1.0000
Dilution: : 1.0000
Do not use Multiplier & Dilution Factor with ISTDs

Signal 1: FID1 B, Back Signal

Peak #	RetTime [min]	Type	Width [min]	Area [pA*s]	Height [pA]	Area %
1	1.587	BB S	0.0397	5805.24512	4810.63623	98.71329
2	27.856	MM	0.2374	1.45847	1.02399e-1	0.02480
3	28.463	MM	0.2512	74.21202	4.92312	1.26191
Totals :				5880.91561	4815.66175	
